# Amphiphilic Protoporphyrin IX Derivatives as New Photosensitizing Agents for the Improvement of Photodynamic Therapy

**DOI:** 10.3390/biomedicines10020423

**Published:** 2022-02-10

**Authors:** Stéphane Desgranges, Petras Juzenas, Vlada Vasovic, Odrun Arna Gederaas, Mikael Lindgren, Trond Warloe, Qian Peng, Christiane Contino-Pépin

**Affiliations:** 1 Equipe Chimie Bioorganique et Systèmes Amphiphiles, Avignon Université, 84000 Avignon, France; stephane.desgranges@univ-avignon.fr; 2 Department of Pathology, Oslo University Hospital, 0379 Oslo, Norway; petras.juzenas@rr-research.no (P.J.); vlada.vasovic@rr-research.no (V.V.); trond.warloe@gmail.com (T.W.); 3 Department of Physics, Faculty of Natural Sciences, Norwegian University of Science and Technology, 7491 Trondheim, Norway; odrun.gederaas@ntnu.no (O.A.G.); mikael.lindgren@ntnu.no (M.L.); 4 Department of Optical Science and Engineering, School of Information Science and Technology, Fudan University, Shanghai 200433, China

**Keywords:** photodynamic therapy, protoporphyrin IX, amphiphiles, photochemical properties, photocytotoxicity

## Abstract

Photodynamic therapy (PDT) is a non-invasive therapeutic modality based on the interaction between a photosensitive molecule called photosensitizer (PS) and visible light irradiation in the presence of oxygen molecule. Protoporphyrin IX (PpIX), an efficient and widely used PS, is hampered in clinical PDT by its poor water-solubility and tendency to self-aggregate. These features are strongly related to the PS hydrophilic–lipophilic balance. In order to improve the chemical properties of PpIX, a series of amphiphilic PpIX derivatives endowed with PEG_550_ headgroups and hydrogenated or fluorinated tails was synthetized. Hydrophilic–lipophilic balance (HLB) and log *p*-values were computed for all of the prepared compounds. Their photochemical properties (spectroscopic characterization, photobleaching, and singlet oxygen quantum yield) were also evaluated followed by the in vitro studies of their cellular uptake, subcellular localization, and photocytotoxicity on three tumor cell lines (4T1, scc-U8, and WiDr cell lines). The results confirm the therapeutic potency of these new PpIX derivatives. Indeed, while all of the derivatives were perfectly water soluble, some of them exhibited an improved photodynamic effect compared to the parent PpIX.

## 1. Introduction

Photodynamic therapy (PDT) is an emerging relatively non-invasive clinical modality, which is increasingly used to treat malignant or non-malignant diseases [1,2,3]. PDT combines two individually non-toxic components of a photosensitive molecule called “photosensitizer” (PS) and light, that can induce tissue damage in the presence of molecular oxygen [4]. PDT requires light with appropriate wavelengths of 600–800 nm (absorbed by the photosensitizer) that undergoes an inter-crossing system from the excited singlet state to the excited triplet state [5]. This PS excited triplet state can exchange an electron or a hydrogen atom with a neighboring substrate, such as cell membrane or organic molecule (Type I photochemical reaction) or transfer energy to ground state molecular oxygen (Type II photochemical reaction). The type I reaction leads to dangerous reactive oxygen species (ROS), among which are superoxide anion radical, hydrogen peroxide, and hydroxyl radical. While the type II photochemical reaction generates the singlet molecular oxygen (^1^O_2_), which is a highly reactive form of oxygen that reacts with many biomolecules including lipids, proteins, and nucleic acids [6]. Both types of photochemical reactions can occur simultaneously in a ratio depending on the type of PS used, as well as the concentration of organic substrate or oxygen molecule. However, most of the PSs (especially porphyrins) are believed to induce cellular damages through a type II mechanism [2].

Since ^1^O_2_ and ROS can only diffuse less than 20 nm within their lifetimes of 10–300 ns in cells [7,8], only the molecules that are proximal to ^1^O_2_ and ROS, thus to the subcellular location of the PS, are directly damaged by PDT. Lipophilic PSs, essentially accumulating in lipid bilayers of membrane structures including mitochondria and endoplasmic reticulum, often cause the cell death towards apoptosis. Alternatively, hydrophilic PSs localized largely in endosomes and lysosomes, have been shown to mainly induce necrosis [9,10,11]. The majority of efficient PSs are hydrophobic, which limits their bioavailability and hampers their systemic administration in vivo with a long retention time in normal tissues of biological systems. For example, a skin accumulation of the FDA approved Photofrin is observed, which constrains patients to avoid sunlight exposure for a prolonged period (up to 2 months) after the PDT treatment. Moreover, hydrophobic PSs tend to aggregate in physiological fluids, which decreases their ^1^O_2_ quantum yield [12]. Other ways to reach deeper organs may be through the use of X-rays [13] and harder ionizing irradiation [14].

The effectiveness of PDT depends on the appropriate combination of several factors, such as the nature of the PS, its pharmacokinetics and tumor localization, photoirradiation parameters (wavelength of irradiation, fluence rate) as well as oxygen availability. The prerequisites for an optimal PS include chemical purity, high ^1^O_2_ quantum yield, selectivity for targeted tissue and rapid accumulation after administration, activation at wavelengths with optimal tissue penetration (near infrared photons are ideally suited for PS activation since they can penetrate deeply in biological tissues), and rapid clearance from the body [15,16].

To date, most of the PS improvements have focused on their purity profile or on photophysical properties, such as the extension of their wavelength absorption towards the near infrared (NIR) region, enhancement of their excitation coefficient or photochemical yield [15,17]. However, a few groups have dedicated their work to improve their biological properties and particularly their bioavailability [18], through water solubility, better cellular uptake, and devoid of aggregation property [19,20].

Several studies have shown that amphiphilic compounds [15] favor the tumor accumulation, probably due to their dual character, that allows for water solubilization. Therefore, this favors the tissue distribution as well as the capacity to interact with the cell membrane to increase their cellular uptake [21,22,23]. Moreover, depending on their self-assembling properties, amphiphilic PSs can minimally aggregate intracellularly or disaggregate upon entering the cell, thereby maximizing ^1^O_2_ production upon photoirradiation. Among the new generation of PSs we focused on protoporphyrin IX (PpIX) [24], a heme precursor which can be endogenously generated from 5-aminolevulinic acid (ALA). PpIX is a widely used PS exhibiting no dark toxicity and a high triplet state lifetime [25,26]. However, due to its hydrophobic porphyrin skeleton, it is poorly soluble in water and its PDT efficiency suffers from a tendency to form aggregates in vivo [3].

The porphyrin macrocycle itself constitutes an interesting scaffold to design Gemini surfactants with a symmetrical structure [27] that may ensure appropriate lipid-like properties [28,29]. In this study, we have synthetized a series of amphiphilic PpIX derivatives with a variable hydrophilic–lipophilic balance (HLB). In order to perform a well exemplified structure–activity relationship study, various hydrogenated or fluorinated hydrophobic tails of different lengths were conjugated to polyethylene glycol (PEG) hydrophilic moieties on the opposite side of the macrocycle core. The photophysical properties (spectroscopic characterization, photobleaching, and ^1^O_2_ quantum yield) of these new amphiphilic PpIX derivatives were evaluated. In addition, their dark toxicity, cellular uptake, subcellular localization, and photocytotoxicity on three different tumor cell lines were studied in vitro and compared to those of the parent PpIX. 

## 2. Materials and Methods

### 2.1. Chemistry

#### 2.1.1. Chemicals and Reagents

For synthesis, protoporphyrin IX was purchased from porphyrin-systems (Frontier scientific, Halstenbek, Pineberg, Germany). In addition, fluorinated alcohol from Fluorochem (Hadfield, United Kingdom) as well as 1H,1H,2H,2H-perfluorohexanethiol and 1H,1H,2H,2H-perfluorooctanethiol were graciously provided by Atochem (Colombes, Paris, France). All of the reagents were from commercial sources and used as received. 

#### 2.1.2. Synthesis and Characterization

The synthetic route leading to PpIX derivatives is a modified procedure, which is derived from the work of Lottner et al. [26].

Briefly, PpIX reacts with a mixture of HBr in acetic acid (33%). Following the removal of HBr and acetic acid, the resulting Bromo-PpIX analogue (**2**) is dissolved in the appropriate alcohol (hydrogenated or fluorinated alcohol of variable lengths) during 16 h, which simultaneously gives the corresponding ether (addition on the two allylic units)/ester (addition on the propionic acid moieties) derivatives **3a**–**h**. The two ester groups are subsequently saponified with 20 eq of LiOH in a mixture of THF and water (3/1). After the acidic treatment leading to compounds **4a**–**h**, 1-amino-ω-methoxy-PEG_550_ (compound **1**) is coupled to acid functions using DCC–HOBt as coupling reagents in DMF. Following the removal of the solvent, the final product is purified over LH20 in DCM–MeOH 1/1 to give the final amphiphilic PpIX derivatives **5a**–**h**. In parallel, the condensation of native PpIX with amino-PEG_550_ (compound **1**), in the presence of DCC–HOBt, led to the PEG_550_-conjugated PpIX (compound **6**).

The synthesis of non-PEGylated intermediates (compounds **2** and **3a**–**h** to **4a**–**h**) was confirmed by 1H and 13C-NMR coupled to HRMS analysis, and by 1H-NMR for polymeric derivatives (compounds **6** and **5a**–**h**) (see NMR spectra and detailed peak assignments in Supplementary Material Section).

### 2.2. Biological Testing

#### 2.2.1. PDT of Tumor Cells with PpIX Derivatives

Tumor cells of the three cell lines were cultured as described above. The cells (1.5 × 10^4^) in 100 μL of the medium were seeded in each well of 96-well plastic tissue-culture plates (Nunc, Thermo Fisher Scientific, Roskilde, Denmark) and left for 24 h for proper attachment to the substratum. Then, the cells were washed twice with PBS and incubated with the medium containing one of the PpIX derivatives, **5a**–**h** (amphiphilic derivatives), **6** (PEG_550_-conjugated PpIX) or the parent PpIX at the concentration of 2.5 μM for 24 h prior to irradiation with a blue lamp (fluence rate: 4.88 mW/cm^2^) for various exposure times. The lamp consisted of a bank of four fluorescent tubes (model 3026, Applied Photophysics, London, UK) emitting light mainly in the region of 410–500 nm with a maximum around 440 nm. The cell survivals were determined with the MTS cell proliferative assay, a method based on the cellular conversion of a tetrazolium compound by viable cells into a colored formazan product, which is soluble in a cell culture medium and can be detected by 492 nm absorbance. Twenty-four hours after light exposure, 20 μL of MTS (Promega Corporation, Madison, WI, USA) were added to each well and the absorbance of 492 nm was measured after 1-h incubation using a well plate reader (Multiskan Ex, Labsystems, Vantaa, Finland).

#### 2.2.2. Uptake of PpIX Derivatives by Cells In Vitro

Three tumor cells lines of 4T1 murine mammary carcinoma cell line, scc-U8 human head and neck squamous cell carcinoma, and WiDr human colon adenocarcinoma were subcultured in RPMI1640 medium (Gibco, Paisley, Scotland, UK) containing 10% fetal calf serum, 100 U/mL penicillin, 100 μg/mL streptomycin, and 1% glutamine at 37 °C in 5% CO_2_ humidified atmosphere. Tumor cells (9.5 × 10^4^) in 400 μL of the medium were seeded in each well of 24-well plastic tissue-culture plates (Nunc) and left for 24 h for proper attachment to the substratum. Then, the cells were washed twice with PBS and incubated with a serum-free medium containing one of the PpIX derivatives, **5a**–**d**, **5f** or parent PpIX at the concentration of 2.5 μM for 24 h in the dark. Thereafter, the cells were washed twice with PBS prior to their addition into a PBS solution by scraping off the cells from the substratum with a Costar cell scraper. The fluorescence of all cell suspensions was determined fluorometrically using a Perkin–Elmer LS50B spectrofluorometer. The excitation wavelength was set at 405 nm and the fluorescent emission was measured at 638 nm using a long-pass cut-off filter (530 nm) on the emission side.

#### 2.2.3. Subcellular Localization of PpIX Derivatives In Vitro

Cells of the three tumor cell lines were grown on Petri dishes with a glass bottom (MatTek Corp., Ashland, MA, USA). Then, they were incubated with the medium containing compounds **5a** and **5b** at the concentration of 2.5 μM for 24 h in the dark. The cells had been washed twice with PBS prior to imaging of the subcellular localization patterns of the fluorescent sensitizers with an Axiovert 40CFL microscope (Carl Zeiss, Jena, Germany) using an oil immersion objective (100 × NA 1.25). A band-pass of 300–400 nm excitation filter was used to detect the PpIX derivatives with a long-pass of 630 nm emission filter.

### 2.3. Photochemical Properties

#### 2.3.1. Spectroscopic Characterization and Singlet Oxygen Production of PDT Agents

For photophysical measurements, standard 1 cm UV quartz cuvettes (Hellma GmbH & Co. KG, Müllheim, Germany) were employed with Teflon caps that allow for flushing with Argon gas to remove oxygen from the solvent. Herein, THF was used as a solvent. Absorbance spectra were obtained using an U-3010 spectrophotometer (Hitachi, Japan) and the software UV solution. PTI Quantamaster 8075-22 equipped with Double Mono 300 spectrometer chambers for both excitation and emission (Horiba Scientific, Tokyo, Japan). The quantum efficiency (QE) was obtained by exciting samples in the solution (5 μM) at 425 nm and recording the emission spectrum. By dividing the integrated fluorescence signal with the absorbance at the excitation wavelength of 425 nm, one obtains the “single point” QE (Table 1).

The singlet oxygen production was demonstrated as the transient singlet oxygen luminescence (1275 nm) of THF solutions with the sample in a standard 90° configuration (excitation and emission path). A tunable OPO laser, NT 342A-SH-10-WW (Ekspla, Vilnius, Lithuania) was used for excitation. For transient recording, a PMT (R5509, Hamamatsu Photonics K.K., Shizuoka, Japan) and interference filter with maximum transmission at 1272.5 nm, as well as a long-pass filter transmitting above 780 nm, were used. An Infiniium BDSU Oscilloscope (Keysight, Santa Rosa, CA, USA) was used to collect the data. Time-gated electronics were used to control the time between laser excitation and the recording of the luminescence transient. The transients were background corrected by subtracting with a signal of the same sample, which was flushed with Argon gas for 10 min. A similar procedure to confirm the singlet oxygen production of Ruthenium complexes for PDT was recently presented in Bogoeva et al. [30].

Shortly, the singlet oxygen yield was estimated by fitting the transient singlet oxygen luminescence to the following expression:

(1) $$I\left(t\right)=\frac{C\cdot k_{PS}}{k_{SO}-k_{PS}}\left[e^{-k_{PS}t}-e^{-k_{SO}t}\right]$$

where $k_{SO}$ is the decay rate of the singlet oxygen luminescence and $k_{PS}$ is the decay rate of the photosensitizer triplet. *C* is a constant proportional to the yield of excited singlet oxygen including instrumental settings, laser power, and number of absorbed *PS* molecules. Using a set of samples under the same experimental conditions and solvent, it can be taken as proportional to the relative singlet oxygen yield. For more details on the theory and related measurements, see, e.g., Snyder et al. [31] and more recently, Nishimura et al. [32].

The excited triplet state absorbance was measured in samples evacuated from oxygen, using an NT 342B-SH-10-WW laser (Ekspla, Vilnius, Lithuania), array detector (Applied Photophysics, Leatherhead, United Kingdom) and a Xenon flash lamp module (Model L9456-01, 5W, Hamamatsu, Japan) with BWSpec software (B&W Tek, Newark, DE, USA). Transient absorption was obtained by firing the excitation OPO laser and flashlamp using a time-gated electronic accessory for the control of triggers and detectors. Prior to the measurement, the sample was flushed with Argon gas for 10 min in order to remove the oxygen that quenched the triplet signal. The recorded spectra were background corrected by subtracting the spectrum of pure solvent. Since the fluorescence was negligible, the fluorescence emission correction was not required (for details of the set-up and the procedure of further analyzing triplet state absorption data, see Glimsdal et al.) [33].

#### 2.3.2. Photobleaching of PpIX Derivatives, **5b** and **5c**, in WiDr Cells after Blue Light Exposure

##### Light Source for In Vitro Cell Experiments

For blue light exposure, the culture dishes (Ø = 6 cm, Nunc, Roskilde, Denmark) were illuminated (from below, at room temperature) using a LumiSource^®^ blue light box (PCI Biotech AS, Oslo, Norway), consisting of four Osram tubes (18 W, peak wavelength 435 nm). The light intensity at the level of the cells was 13 mW/cm^2^, measured with an Optometer UDT model 161, radiometer-photometer (united Detector Technology, Culver City, CA, USA) giving a total light dose of 3.1 J/cm^2^ at the cell level during a 4 min illumination period.

##### Cell Culture

The cell line WiDr was cultured in RPMI 1640 medium containing 10% (*v*/*v*) FCS, L-glutamine (80 mg/L), streptomycin (100 U/mL), and grown in an atmosphere of 95% air and 5% CO_2_ at 37 °C, subcultured approximately twice a week.

##### Fluorescence Measurements and Photobleaching Experiments

WiDr cells were seeded in culture dishes (2.0 × 10^6^ cells per dish) and grown for 24 h before incubation with compound **5b**, **5c** or native protoporphyrin IX (2.5 μM, 24 h). Following incubation, the cells were washed three times (PBS) and blue light illuminated (in PBS) by LumiSource^®^ (435 nm, 0–7.74 J/cm^2^). The cells were detached by accutase (1 mL, 500–720 U/mL, Sigma-Aldrich, 37 °C) for 3–10 min (depending on the light dose) prior to centrifugation (1500 rpm, 5 min) and resuspension in PBS. By manual counting (Bürcher chamber, Merck, Darmstadt, Germany), a final cell concentration of 1 million/mL in PBS was prepared. The cell suspensions (2 mL) were immediately transferred to quartz cuvettes for fluorescence measurements recorded employing a PTI Quantamaster as described in 2.3.1 at Ex: 410 nm and Em: 550–780 nm. As a control, the fluorescence spectra of three different cell samples incubated with PpIX (2.5 μM, 24 h) were analyzed using Ex: 405 nm. All of the samples including the control cells (“no light” and “no photosensitizer”) were protected from light by aluminum foil during the experimental set-up.

### 2.4. Software Employed

The software MarvinSketch (version 21.9.0, calculation module developed by ChemAxon, http://www.chemaxon.com/marvin/sketch/index.php, accessed on 13 December 2021), using the VG function based on the atomic log *p* increments method, was used to predict the log *p*-values [34]. The log *p*-value is a quantitative descriptor of lipophilicity or hydrophobicity. The same MarvinSketch software was also used to assess the HLB of PpIX derivatives by applying the Davies’ method [35]. The latter method, with a scale from 0 to 20, is based on the chemical groups of the molecule, where 0 indicates a completely hydrophobic molecule, while 20 indicates a completely hydrophilic molecule. According to Fung, a value from 8–12 indicates an oil–water emulsifier, a value from 13–16 is typical of detergents, and a value from 15–20 indicates a hydrotropic behavior [36].

## 3. Results and Discussion

### 3.1. Synthesis and Characterization

**Scheme 1:**
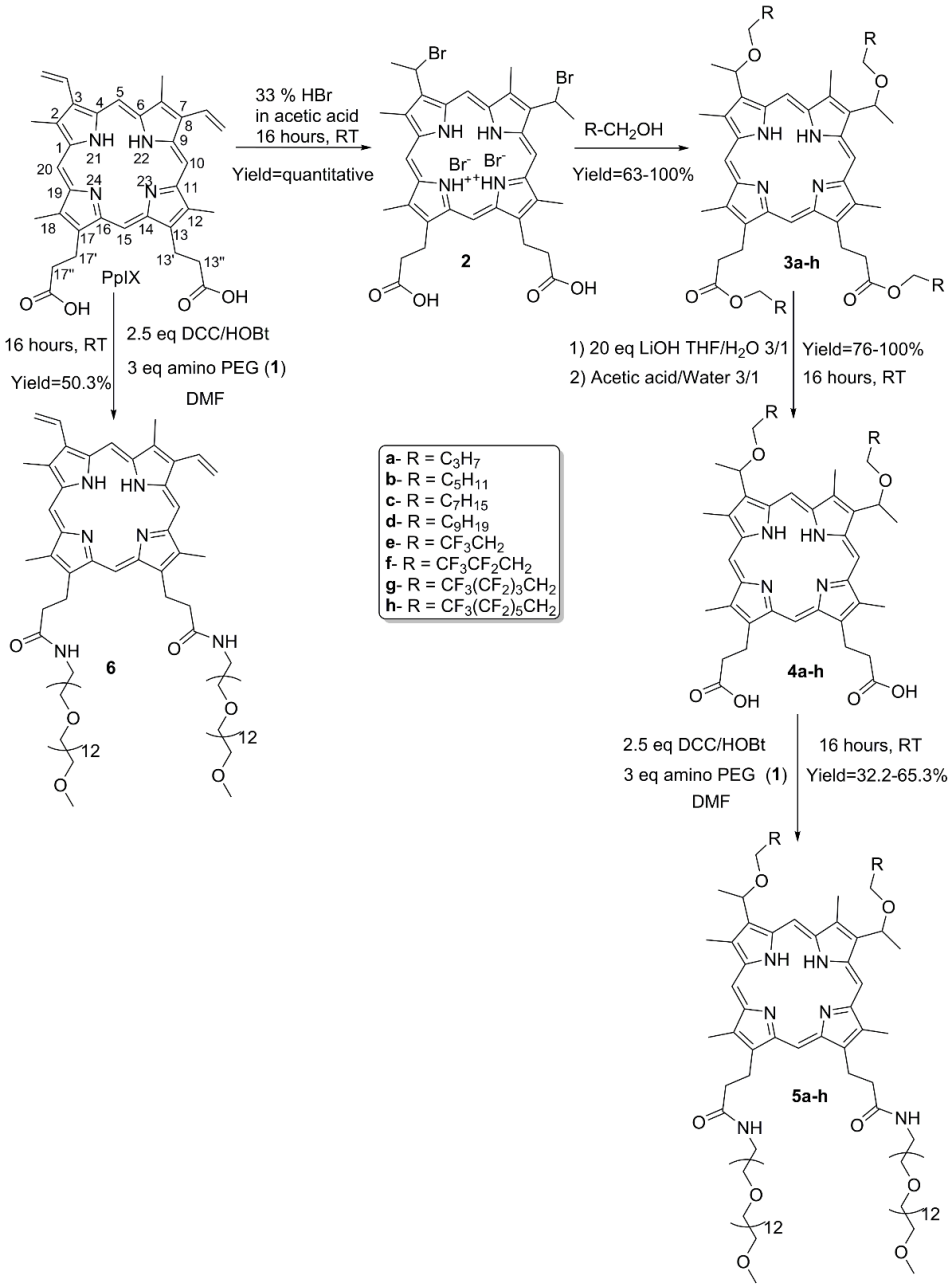
Synthesis of PpIX derivatives.

Porphyrins belong to the tetrapyrrole family, whose chemistry, photo-properties, and supra-molecular properties are extensively reviewed [24,37]. In our effort to produce an efficient synthesis of amphiphilic PSs, PpIX was chosen as a starting material to prepare the Gemini-like analogues. Indeed, PpIX exhibits two different functions on opposite sides of the porphyrin macrocycle, namely two propionic acid moieties at positions 13 and 17 and two vinyl groups at positions 3 and 8 (Scheme 1). A non-ionic polar headgroup was grafted to the acidic functions through an amide bond, more specifically a flexible PEG chain with a number of 12 repeating ethylene glycol units (i.e., PEG_550_ moiety). The PEG chain length remained constant, as it was long enough to ensure the final water solubility of the macromolecule. Hydrophobic chains of variable lengths, hydrogenated or fluorinated, were incorporated at the vinyl site to study the impact of hydrophobicity on subcellular localization and cytotoxic efficiency. Fluorinated tails were introduced to limit the detergency of the resulting amphiphilic PpIX analogues due to the dual hydrophobic and lipophobic nature of fluoroalkyl chains. The length of hydrocarbon chains varies from 4 to 10 carbons (compounds **5a**–**5d**), while the fluorinated tails length varies from 1 to 6 fluorinated carbons (compounds **5e**–**5h**). In addition, an ethylene spacer group is only located near the alcohol function to avoid synthetic issues related to the CF_2_ electron-withdrawing effect. Moreover, as the tetrapyrrole core is hydrophobic itself, we also synthetized a PEG_550_-conjugated PpIX analogue (compound **6**) bearing only two PEG_550_ moieties, in order to evaluate the impact of hydrophobic tails on the photophysical properties, as well on the cellular uptake and cytotoxicity of PpIX derivatives.

The synthetic pathway was efficient and straight forward, since the final products were obtained in four steps with overall yields ranging from 32.2% to 65.3%. Our approach was to use native PpIX as a central core and to modify the propionic and vinylic groups in an orthogonal manner. The amino-PEG_550_ (**1**) used in the synthesis was easily obtained in three steps from the commercially available monomethyl-PEG_550_ alcohol with an overall yield of 62%. Briefly, the PEG-alcohol was converted into its mesylate derivative, which then underwent a nucleophilic substitution with NaN_3_ to give the corresponding azido-PEG_550_ derivative. The latter was reduced by LiALH_4_ to give the final amino-PEG_550_ compound (**1**).

The vinyl groups can undergo several types of reaction, such as reduction, oxidation, substitution, elimination, electrocyclic reactions, and olefin metathesis [2,37]. The well-known hydrobromination procedure for the functionalization of porphyrin vinyl groups was applied. In addition, the reaction was performed using the HBr–AcOH system to quantitatively yield the corresponding dibromo analogue (**2**). Then, the introduction of hydrophobic chains through ether bonds by the reacting compound (**2**) with the suitable alcohol [2,23] led to the expected ethers. In the meantime, esterification of the two carboxylic acid groups afforded the tetra-substituted PpIX series (**3a**–**h**) with yields ranging from 63% to 100%. The simultaneous esterification is due to the presence of the two protonated pyrrole rings, which act as catalysts [38]. The hydrolysis of the ester bond of compounds **3a**–**h** with LiOH in a mixture of water and THF, followed by an acidic treatment, led to compounds 4a–h, with yields ranging from 76% to 100%. The amino-PEG_550_ hydrophilic moiety 1 was finally introduced via the conventional peptidic coupling method using DCC–HOBt as a coupling reagent to yield the amphiphilic PpIX analogues, **5a**–**h**. The final compounds were purified by LH20 in MeOH with yields ranging from 32% to 65%. The PEG_550_-conjugated PpIX (6) was readily obtained by coupling native PpIX with amino-PEG_550_ (1) in the presence of DCC–HOBt in 50.3% yield.

The octanol–water partition coefficient (log *p*) is a suitable descriptor to predict the intracellular localization of compounds, according to their hydrophilic–lipophilic properties. For example, a better affinity towards biological membranes is correlated with the compound’s higher hydrophobicity [7,39]. The MarvinSketch software of ChemAxon was used to calculate the HLB and log *p* of all final PpIX derivatives (listed in Table 1) in order to compare them in terms of hydrophobicity. With regards to all of the series, except for compound **6** which lacks the hydrophobic tail, the HLB and log *p* are inversely correlated. The compounds have calculated HLB values ranging from 12.14 to 18.75, indicating that they are all perfectly water soluble. Most of the PpIX derivatives possess an HLB between 13 and 16 relative to the detergency properties, according to Fung [36]. In parallel, their log *p* ranges from 3.3 for compound **6**, which lacks hydrophobic chains to 12.6 for compound **5h**, which comprises two perfluorinated chains of six carbons. As expected, for the same tail length, fluorinated derivatives exhibit higher log *p*-values than the hydrogenated analogues [40]. For example, compound **5c** endowed with two C_8_ hydrocarbon chains (C_8_H_17_) has a log *p* of 7.4, while for the same tail length, compound **5h** endowed with two C_8_ hemifluorinated chains (C_2_H_4_C_6_F_13_) has a log *p* of 12.6.

### 3.2. Biological Assays

#### 3.2.1. PDT of Tumor Cells with PpIX Derivatives

**Figure 1:**
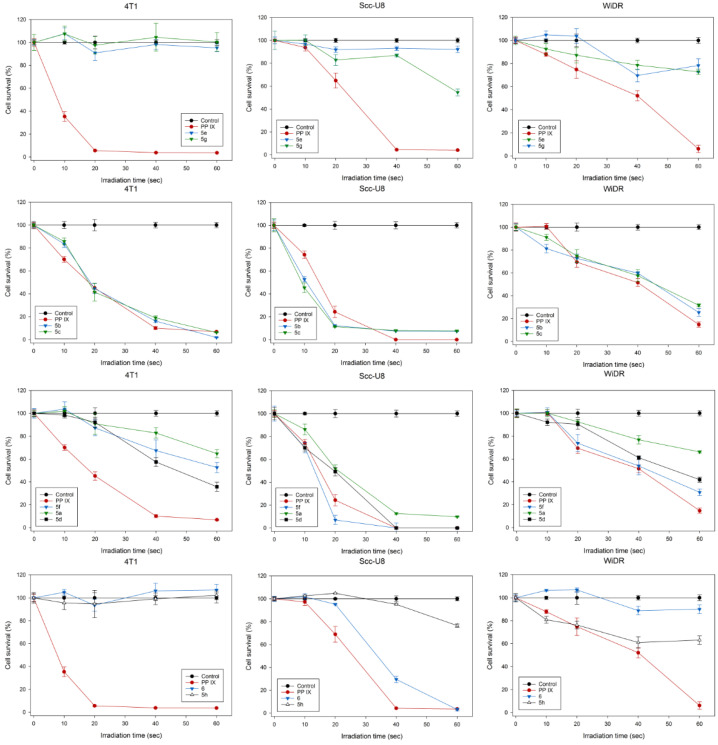
PDT of tumor cells with native PpIX and PpIX derivatives, **5a**–**h** and **6**.

Figure 1 shows the cell survivals after PDT with PpIX or PpIX derivatives **5a**–**h** (amphiphilic derivatives) and 6 (PEG_550_-PpIX) as a function of light exposure times in the tumor cell lines of 4T1, scc-U8, and WiDr. The cell survivals were decreased with increasing light exposure times during PDT with PpIX, as well as compounds **5b** and **5c** in all of the three cell lines studied. With a 60-s light exposure, the PDT killed almost all of the 4T1 and scc-U8 cells and about 70% of the WiDr cells. Both compounds **5b** and **5c** had a considerable PDT efficiency to the parent PpIX in all of the three cell lines. Similarly, the compound **5f**-mediated PDT killed 70–100% of the cells in both scc-U8 and WiDr cell lines, but significantly less than compounds **5b** and **5c**, as well as PpIX in the 4T1 cells. The PDT killing effect of the other PpIX derivatives was lower than PpIX in all of the cell lines. In general, lipophilic photosensitizers have a stronger photodynamic effect than the hydrophilic dyes on killing cells in vitro due to the localization of cell membranous structures [7]. However, the hydrophobic dyes are not water soluble and cannot be used for the drug administration in the in vivo biological models, due to the fact that they are easily aggregated in water with reduced photodynamic efficiency. Water soluble amphiphilic sensitizers with a relatively long C-chain hydrophobic tail may be suitable for use in biological systems in vivo. Among the currently proposed PpIX derivatives, it seems that the “limit” forms of the series, compounds **5h** (endowed with the longer fluorinated chains, log *p* = 12.6) and **6** (lacking hydrophobic tails, log *p* = 3.3) are indeed totally inactive (both are inactive on 4T1 cells or compound **6** is inactive on the WiDr cell line) or exhibit a low cell killing effect (for compound **5h** on scc-U8 and WiDr cells). The stronger photodynamic effects were obtained by compounds **5b** and **5c** with log *p*-values between 5.82 and 7.4 and endowed with C6- and C8-chains, respectively. Of note, compound **5f** bearing two C4 hemifluorinated chains (C_2_H_4_C_2_F_5_) but with a log *p*-value of 5.92, has a photodynamic effect which is comparable to compounds **5b** and **5c** on the scc-U8 and WiDr cell lines. While compound **5a**, which is endowed with two C4 hydrogenated chains (C_4_H_9_) and a log *p*-value of 4.23, has a low killing effect on the 4T1 and WiDr cells. These results show that a fine-tuning of the amphiphilic coating of PpIX might improve its photodynamic effect independently of the cell line, although the mechanism of action of each resulting PpIX derivative is probably complex and cell specific [41]. This may be due to the fact that amphiphilic dyes are localized in multiple cellular structures, and thus target several cellular structures to generate an effective damage to the cells after light irradiation.

#### 3.2.2. Uptake of PpIX Derivatives by Cells In Vitro

**Figure 2:**
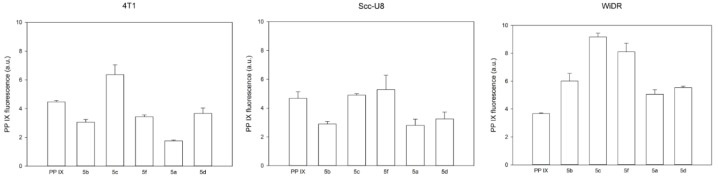
Cellular uptake of PpIX and PpIX derivatives, **5a**–**d** and **5f**.

Figure 2 shows the uptake of **5a**–d, **5f**, and parent PpIX (both at 2.5 μM) by the tumor cell lines of 4T1, scc-U8, and WiDr in vitro. The compounds were studied at very low concentrations (2.5 μM) to avoid aggregation. Of note, a dynamic light scattering (DLS) analysis showed that at this concentration, all of the PpIX derivatives were under a monomeric form (see DLS data in Supplementary Material) [42]. In general, the results show that there is an individual variation of cellular uptake of the five PpIX derivatives within a single cell line and also among the three cell lines. The cellular uptake of the PpIX derivatives is similar to the parent PpIX in the 4T1 and scc-U8 cell lines, while significantly more than the parent PpIX in the WiDr cells (*p*-values = 0.013 for **5b**, <0.001 for **5c**, 0.002 for **5f**, 0.015 for **5a**, and <0.001 for **5d**). Compounds **5c** and **5f** appeared to be taken up significantly more by the cell lines than the other PpIX derivatives, except for the 4T1 cells where only compound **5c** was more internalized. Of note, the two PpIX derivatives, **5c** and **5f**, have very close HLB values of 14.83 and 14.81, respectively. The WiDr cells of the five PpIX derivatives were taken up more than the other two cell lines. These results show that according to the cell line, the uptake can be significantly different. Moreover, it should be mentioned that PBS was used to measure the amount of PpIX and its derivatives, **5a**–**d** and **5f**, which are all water soluble in the cells. Furthermore, due to the limited solubility of the parent PpIX in the buffer, its cellular uptake was probably underestimated.

#### 3.2.3. Subcellular Localization of PpIX Derivatives In Vitro

**Figure 3:**
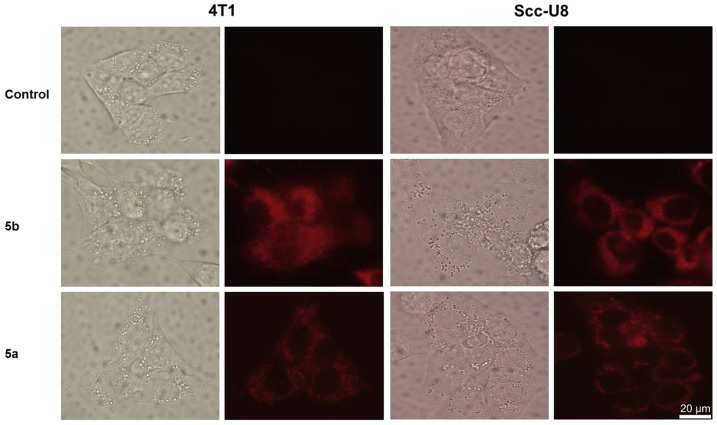
Cellular localization of PpIX derivatives, **5a** and **5b**, in 4T1 and Scc-U8 cells. Scale bar = 20 μm.

**Figure 4:**
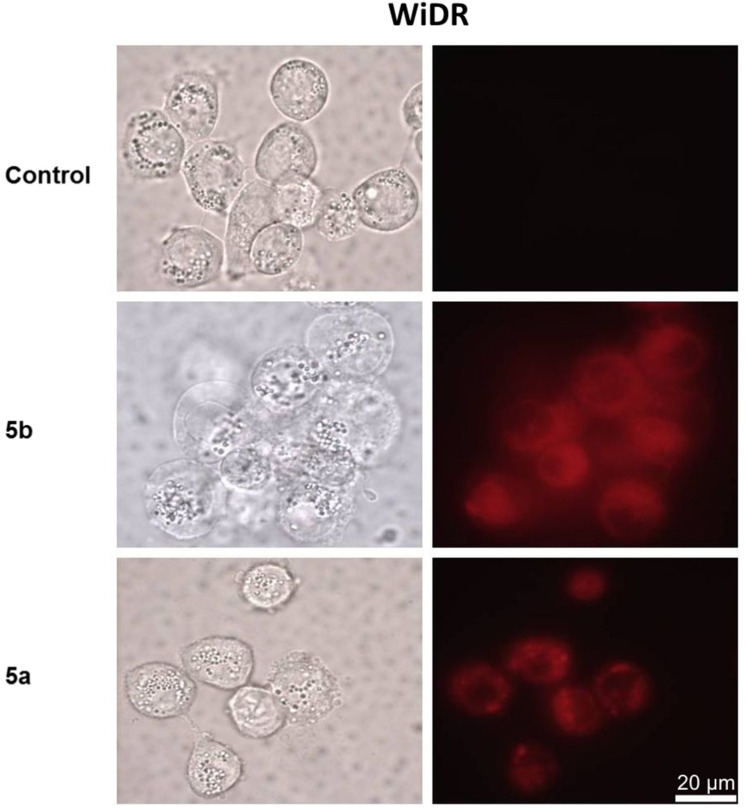
Cellular localization of PpIX derivatives, **5a** and **5b**, in WiDR cells. Scale bar = 20 μm.

Two PpIX derivatives, **5a** and **5b**, with different lipophilic properties (log *p*-values of 4.23 and 5.82, respectively) were chosen to study the subcellular localization in the three cell lines of 4T1, Scc-U8, and WiDr. Compound **5b**, with a relatively higher lipophilicity, demonstrated both diffuse and granular patterns in all of the three cell lines studied (Figure 3 and Figure 4), suggesting that it was localized in both biomembrane structures and lysosomes. Compound **5a**, with a lower lipophilic character, showed only the lysosomal localization as a granular pattern (Figure 3 and Figure 4). In general, these results are consistent with previous reports that hydrophilic dyes in the lysosomes and amphiphilic are located in both membranous structures and lysosomes, while hydrophobic sensitizers are mainly localized in the cellular membranous structures [7]. Compound **5b** is esterified with a C6-chain aliphatic alcohol, while compound **5a** is esterified with a C4-chain aliphatic alcohol. A longer C-chain anchored to the porphyrin core displays a relatively more hydrophobic behavior, as illustrated by the log *p*-values. Therefore, this favors a subcellular localization in the cell membranous structures.

### 3.3. Photochemical Properties

#### 3.3.1. Results of Photophysical Properties

**Figure 5:**
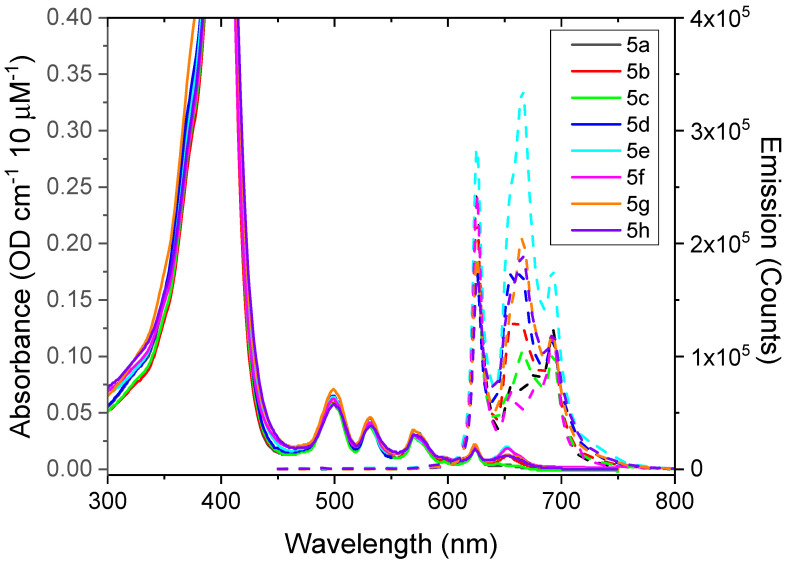
Absorbance (solid lines) and emission (dashed) spectra of compounds dissolved in H_2_O (10 μM). Herein, the vertical scale of the absorbance is adjusted to focus on the Q-bands in the 500–650 nm range. The dashed curves are fluorescence emission spectra upon excitation at 425 nm.

The absorbance and fluorescence spectra of **5a**–**h** compounds, which are recorded using water as a solvent, are displayed in Figure 5. The absorption shows the characteristic Q-bands in the wavelength range of 490–650 nm, which is indeed very similar for all of the compounds studied. Similar splitting of the Q-bands can be seen in most free-base porphyrin forms, such as in dendrimer substituted tetra-phenyl porphyrins [43]. The splittings are caused by a vibrational substructure of several vibrational modes of the ring structure around 1600 cm^−1^, and can be assigned to 1-0, 0-0 (Q_y_) and 1-0, 0-0 (Q_x_) vibrational substructure for the transitions at approximately at 500, 535, 570, and 620 nm (Figure 5 and Figure S3), where x is taken as the direction along the N–H bond [44]. Interestingly, for the porphyrins investigated here, there are also some additional bands resolved towards the IR side. The quantum efficiencies (QE) relative to PpIX were obtained using THF as a solvent and are presented in Table 1.

It is logical to observe similar spectral characteristics in all of the amphiphilic compounds since they all have undergone the same chemical reaction. Moreover, only the periphery of PpIX was modified. It is well known that the modification of the PpIX core significantly affects its photophysical properties [45]. However, the spectral properties are slightly affected, which seems to be associated with the anchoring of the PEG moiety through the amide bond, since similar spectra are observed between the PEG_550_-conjugated PpIX and amphiphilic PpIX derivatives, which are presented here.

It was not possible to directly detect a singlet oxygen from the water solvents of these compounds, owing to the low solubility of oxygen in water in combination with the strong quenching of triplet states that usually occur in water. On the other hand, organic solvents are known to have approximately an order of magnitude higher oxygen solubility than more polar solvents, such as water, methanol, and DMSO [46,47]. There are also less strong vibrations from the solvent molecules that can quench the intermediate triplet state. Therefore, THF was used for the demonstration of triplet state formation and energy transfer to form a singlet oxygen in these compounds. In order to confirm the production of singlet oxygen of PpIX derivatives, the triplet excited state absorption (TESA) recorded for UV excitation (lex = 355 nm), as well as the direct transient singlet oxygen luminescence at 1275 nm upon excitation at 425 nm, were investigated (Figure S3 of the Supplementary Materials).

The linear absorption spectra recorded for the 5 μM THF solutions used for TESA all have a ground state absorbance in the range between OD 0.1–0.2 at 355 and 425 nm (data not shown). Therefore, there are no issues with self-absorption at these wavelengths. In order to prove the existence of triplet states, the excited state absorption was measured by exciting an Argon gas flushed sample with a ns laser pulse at 355 nm, and recording the full spectrum with a broad band flash lamp and a gated detector, a few microseconds after the excitation pulse. In Figure S3 (left panel), these spectra are shown as broad features covering the region in the range 300–480 nm. In addition, there are sharper peaks, showing up as depleted (negative) absorption bands, overlayed onto the broad triplet absorption, notably the Q-bands in the range 490–650 nm (for details on how to interpret these spectra, see Glimsdal et al. [33]). By repeating the procedure for a sample that contains oxygen, the triplet absorption signal is strongly quenched and diminished (data not shown).

**Figure 6:**
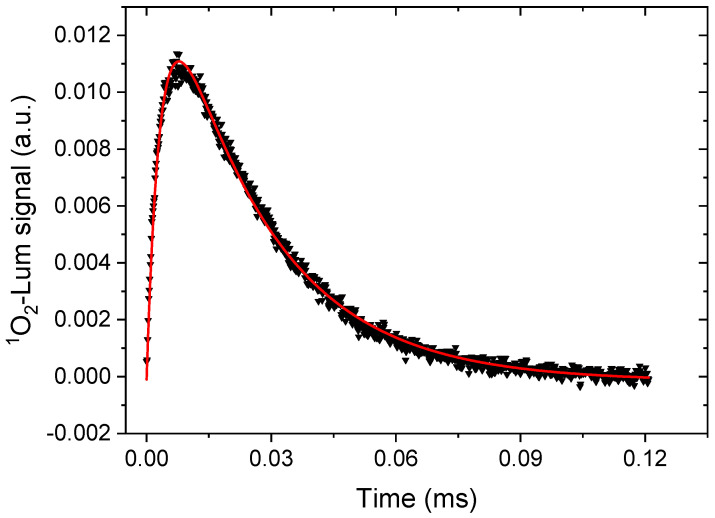
Transient singlet oxygen luminescence at 1275 nm upon excitation of compound **5d** at 425 nm in THF. The solid red curve is fit to a double exponential, as described in the text.

A singlet oxygen yield is difficult to quantify accurately. Moreover, it strongly depends on the solvent, solvent oxygen content, and many factors (see [32,48] for detailed studies of substituted PpIX variants). Here, it was estimated from the transient luminescence of singlet oxygen that has a characteristic line-shape, as shown in Figure 6. For clarity, we only show the results for compound **5d** using air-saturated THF as a solvent. Herein, the transient signal has been background corrected with the signal from the same sample bubbled with Argon gas for 10 min, to remove the oxygen dissolved in the solvent. The time-trace was fitted to a double exponential function giving two characteristic time decays. In this case, the rise time was determined as 3.0 ± 0.02 μs and the decay as 24.4 ± 0.08 μs using Equation (1) (Experimental Section). The former represents the decay of the triplet state of the sensitizer, whereas the longer decay is very characteristic for the singlet oxygen itself, strongly depending on the solvent, e.g., [31,49]. The results of six representative compounds along with the fitted parameters are shown in Supplementary Material (Figure S3 (right panel, Table S1)). The substituted derivatives of PpIX all show a similar or slightly higher singlet oxygen yield than the bare original PpIX molecule. The latter points to an efficient inter-system that crosses to triplet states. However, these triplet states are dark and did not appear as phosphorescence in the oxygen evacuated samples. Taken together, the transient absorption and singlet oxygen luminescence experiments confirmed the energy transfer from the ground state absorption via the triplet state to the proximate dissolved oxygen molecules in order to obtain the singlet oxygen. The singlet oxygen yield is similar to or better than the PpIX itself.

#### 3.3.2. Photobleaching of PpIX Derivatives, **5b** and **5c**, in WiDr Cells after Blue Light Exposure

Based on the promising in vitro results (especially to the phototoxicity assays) on the three lines of human cancer cells, the more potent PpIX derivatives, **5b** and **5c**, were studied with respect to the photobleaching processes, as described by Gederaas et al. [50] using four different blue light doses (0.39–7.74 J/cm^2^) on WiDr human colon adenocarcinoma cells. All of the bleaching experiments were performed in parallel with the native PpIX (2.5 μM, 24 h) under similar instrumental settings. Control cells as “no light” and “no photosensitizer” were also included.

**Figure 7:**
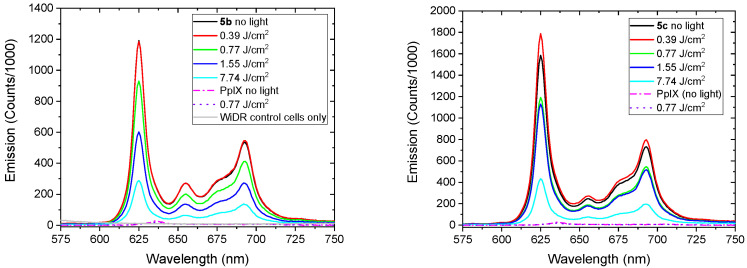
Representative fluorescence spectra of compounds **5b** (**Left**) and **5c** (**Right**) blue light doses. Spectra are averages of 3–5 experiments except for control experiments with PpIX and cells only.

**Figure 8:**
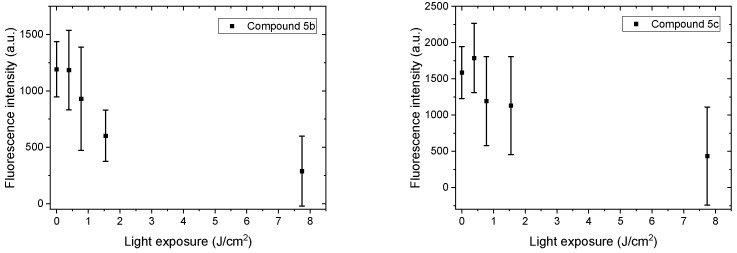
Bleaching data of compounds **5b**- (**Left**) and **5c**- (**Right**) incubated WiDr cells. The standard deviations are based on 3–5 experiments from data presented in Figure 7.

The fluorescence spectra from representative experiments for compounds **5b** and **5c** are shown in Figure 7. In addition, the bleaching spectra including all of the measurements from 4–5 independent experiments are shown in Figure 8. The photobleaching properties of **5b**- and **5c**-incubated WiDr cells illustrate a reduction in the photobleaching of **5b**, to a large extent as 81.2% and 74.2% for compound **5c** after blue light exposure, from 0–7.74 J/cm^2^ (0–600 s). As a control, the fluorescence of PpIX cell suspensions (n = 3) was compared to the fluorescence measurements using Ex: 405 nm [51], which results in a reduction of 13.3% compared to the excitation at 410 nm. From these data, it was concluded that fluorescence measurements at Ex: 410 nm were sufficient for compounds **5b**, **5c**, and native PpIX-incubated WiDr cells in the same experiment. Of note, the fluorescence of PpIX-incubated cells without light was about 50 times less compared to the fluorescence of **5b**- and **5c**-incubated cells without light, using an excitation wavelength of 410 nm.

The bleaching curves were given by the generic formula:

F(t) = A ∗ exp(−k ∗ t)(2)

then, the fitted rates (k) and amplitude factors A were stated for each case.

By comparing the photobleaching data in this study with the published results on the tetrapyrrole photosensitizer TPCS_2a_ (Amphinex^R^), on the same cell line [50], a relatively fast photobleaching process was observed for both compounds **5b** and **5c**, and possibly faster by compound **5b**. Moreover, based on the 5-aminolevulinic-based photobleaching studies, using confocal scanning microscopy on single rat bladder cancer cells (AY27), a dramatic decrease in PpIX fluorescence was observed the first 20 s of continuous light exposure. The fluence rate in the AY27 study corresponded to a light dose of 45 J/cm^2^ after 0.5 s [52]. Depending on the photosensitizer and actual light dose, the rapid photobleaching may cause incomplete tumor destruction. For an optimizing illumination with respect to the cell type, light intensity, and exposure time, further experiments are necessary.

The photobleaching process of protoporphyrin IX derivatives, **5b** and **5c**, were studied in WiDr cells using four different blue light doses and compared with the bleaching of native protoporphyrin IX in the same experiments. The present results document a greater photobleaching in WiDr cells of the less lipophilic PpIX derivative, compound **5b** (log *p* = 5.82) compared to compound **5c** (log *p* = 7.4).

## 4. Conclusions

A novel class of amphiphilic PpIX derivatives was synthesized, bearing two PEG_550_ headgroups (ensuring hydrophilicity) and two hydrogenated or hemifluorinated tails (ensuring hydrophobicity) of different lengths (e.g., containing 4 to 10 carbons). Their synthesis was straight forward by taking advantage of the four anchoring points of the porphyrin core to afford Gemini-like surfactants with a PpIX central scaffold. The resulting amphiphilic PpIX derivatives were fully characterized by NMR analysis, and their hydrophilic–lipophilic balance (HLB) and partition coefficient (log *p*) were computed using the MarvinSketch software of ChemAxon. The photochemical properties (spectroscopic characterization, photobleaching, and singlet oxygen quantum yield) were evaluated followed by in vitro assays to assess their cellular uptake, subcellular localization, and phototoxic efficiency on three tumor cell lines (4T1, scc-U8, and WiDr cell lines). Although further investigation is needed to build a structure–activity relationship, our results confirm the therapeutic potency of this new family of PpIX derivatives. Furthermore, all of these derivatives are not only water soluble, but some of them also exhibit a higher photodynamic effect than the native PpIX.

## Figures and Tables

**Table 1:** Octanol–water partition coefficient (log *p*) and hydrophilic–lipophilic balance (HLB) of porphyrins 6 and **5a**–h computed by the MarvinSketch software. Additionally, the fluorescence quantum efficiencies (QE) of the compounds in THF relative to PpIX (set to 1.0 in column for PpIX) are included (n.d. means ‘not determined’).

	5a	5b	5c	5d	5e	5f	5g	5h	6	PpIX
Log P	4.23	5.82	7.4	8.99	4.25	5.92	9.26	12.6	3.3	
HLB	18.75	15.28	14.83	14.41	15.34	14.81	13.89	12.14	16.13	
QE	1.2	1.4	1.1	n.d	1.2	1.1	1.1	0.9	n.d.	1.0

## Data Availability

The authors agree with MDPI Research Data Policies.

## Supplementary Materials

The following supporting information can be downloaded at: https://www.mdpi.com/article/10.3390/biomedicines10020423/s1. Figures S1 and S2: DLS data. Figure S3: Spectral data of triplet excited state absorption (left) and transient singlet oxygen luminescence (right); Table S1: Summary of fitting parameters to transient singlet oxygen luminescence; Figures S4–S71: 1H and 13C-NMR spectra of all PpIX derivatives.
