# Supplementary material for: Amphiphilic Protoporphyrin IX Derivatives as New Photosensitizing Agents for the Improvement of Photodynamic Therapy
Source: Biomedicines. 2022 Feb 10;10(2):423. doi: 10.3390/biomedicines10020423 (PMC8962274; doi:10.3390/biomedicines10020423)
Supplement: Supplementary file 1 [file biomedicines-10-00423-s001.zip › biomedicines-1557766-supplementary.pdf]

Supplementary material

Amphiphilic Protoporphyrin IX Derivatives as New Photosensitizing Agents for the Improvement of Photodynamic Therapy

Stéphane Desgranges [1], Petras Juzenas [2], Vlada Vasovic [2], Odrun Arna Gederaas [3], Mikael Lindgren [3], Trond Warloe [2], Qian Peng [2,*] and Christiane Contino-Pépin [1,*]

[1]*Equipe Chimie Bioorganique et Systèmes Amphiphiles, Institut des Biomolécules Max Mousseron, UMR 5247, Avignon Université, 84911 Avignon, France; christine.pepin@univ-avignon.fr*

[2]*Department of Pathology, Oslo University Hospital, Oslo, Norway; qian.peng@rr-research.no*

[3]*NTNU, Trondheim, Norway;*

* Corresponding authors: christine.pepin@univ-avignon.fr and qian.peng@rr-research.no

Table S1: Results of fits to transient singlet oxygen luminescence 13

Figure S1: Intensity of scattered light (kcps) as a function of PpIX derivative concentration (µM). Intensity plotted for concentrations ranging from 500 µM to 0.5 µM. 12
Figure S2 : Example of size distribution (DLS analysis in volume-weighted distribution CONTIN) of compound 5d at 500 µM (at this concentration the compound is self-assembled within micelles). 13
Figure S3: 13
Figure S4: Figure S3: ^{1}H NMR spectrum of compound PEG-OMs in $CDCl_3$ 14
Figure S5: ^{13}C NMR spectrum of compound PEG-OMs in $CDCl_3$ 14
Figure S6: ^{1}H NMR spectrum of compound PEG-N3 in $CDCl_3$ 15
Figure S7: ^{13}C NMR spectrum of compound PEG-N3 in $CDCl_3$ 15
Figure S8: ^{1}H NMR spectrum of compound 1 in $CDCl_3$ 16
Figure S9: ^{13}C NMR spectrum of compound 1 in $CDCl_3$ 16
Figure S10: ^{1}H NMR spectrum of compound 3a in $CDCl_3$ 17
Figure S11: ^{13}C NMR spectrum of compound 3a in $CDCl_3$ 17
Figure S12: ^{1}H NMR spectrum of compound 3b in $CDCl_3$ 18
Figure S13: ^{13}C NMR spectrum of compound 3b in $CDCl_3$ 18
Figure S14: ^{1}H NMR spectrum of compound 3c in $CDCl_3$ 19
Figure S15: ^{13}C NMR spectrum of compound 3c in $CDCl_3$ 19
Figure S16: ^{1}H NMR spectrum of compound 3d in $CDCl_3$ 20
Figure S17: ^{13}C NMR spectrum of compound 3d in $CDCl_3$ 20
Figure S18: ^{1}H NMR spectrum of compound 3e in $CDCl_3$ 21
Figure S19: ^{13}C NMR spectrum of compound 3e in $CDCl_3$ 21
Figure S20: ^{19}F NMR spectrum of compound 3e in $CDCl_3$ 22
Figure S21: ^{1}H NMR spectrum of compound 3f in $CDCl_3$ 22

Figure S22: ^{13}C NMR spectrum of compound 3f in $CDCl_3$ 23
Figure S23: ^{19}F NMR spectrum of compound 3f in $CDCl_3$ 23
Figure S24: ^{1}H NMR spectrum of compound 3g in $CDCl_3$ 24
Figure S25: ^{13}C NMR spectrum of compound 3g in $CDCl_3$ 24
Figure S26: ^{19}F NMR spectrum of compound 3g in $CDCl_3$ 25
Figure S27: ^{1}H NMR spectrum of compound 3h in $CDCl_3$ 25
Figure S28: ^{13}C NMR spectrum of compound 3h in $CDCl_3$ 26
Figure S29: ^{19}F NMR spectrum of compound 3h in $CDCl_3$ 26
Figure S30: ^{1}H NMR spectrum of compound 4a in $CDCl_3$ 27
Figure S31: ^{13}C NMR spectrum of compound 4a in $CDCl_3$ 27
Figure S32: ^{1}H NMR spectrum of compound 4b in $CDCl_3$ 28
Figure S33: ^{13}C NMR spectrum of compound 4b in $CDCl_3$ 28
Figure S34: ^{1}H NMR spectrum of compound 4c in $CDCl_3$ 29
Figure S35: ^{13}C NMR spectrum of compound 4c in $CDCl_3$ 29
Figure S36: ^{1}H NMR spectrum of compound 4d in $CDCl_3$ 30
Figure S37: ^{13}C NMR spectrum of compound 4d in $CDCl_3$ 30
Figure S38: ^{1}H NMR spectrum of compound 4e in $CDCl_3$ 31
Figure S39: ^{13}C NMR spectrum of compound 4e in $CDCl_3$ 31
Figure S40: ^{19}F NMR spectrum of compound 4e in $CDCl_3$ 32
Figure S41: ^{1}H NMR spectrum of compound 4f in $CDCl_3$ 32
Figure S42: ^{13}C NMR spectrum of compound 4f in $CDCl_3$ 33
Figure S43 : ^{19}F NMR spectrum of compound 4f in $CDCl_3$ 33
Figure S44: ^{1}H NMR spectrum of compound 4g in $CDCl_3$ 34
Figure S45: ^{13}C NMR spectrum of compound 4ga in $CDCl_3$ 34
Figure S46: ^{13}C NMR spectrum of compound 4g in $CDCl_3$ 35
Figure S47: ^{1}H NMR spectrum of compound 4h in $CDCl_3$ 35
Figure S48: ^{13}C NMR spectrum of compound 4h in $CDCl_3$ 36
Figure S49: ^{13}C NMR spectrum of compound 4h in $CDCl_3$ 36
Figure S50: ^{1}H NMR spectrum of compound 5a in $CDCl_3$ 37
Figure S51: ^{13}C NMR spectrum of compound 5a in $CDCl_3$ 37
Figure S52: ^{1}H NMR spectrum of compound 5b in $CDCl_3$ 38
Figure S53: ^{13}C NMR spectrum of compound 5b in $CDCl_3$ 38
Figure S54: ^{1}H NMR spectrum of compound 5c in $CDCl_3$ 39
Figure S55: ^{13}C NMR spectrum of compound 5c in $CDCl_3$ 39
Figure S56: ^{1}H NMR spectrum of compound 5d in $CDCl_3$ 40
Figure S57: ^{13}C NMR spectrum of compound 5d in $CDCl_3$ 40
Figure S58: ^{1}H NMR spectrum of compound 5e in $CDCl_3$ 41
Figure S59: ^{13}C NMR spectrum of compound 5e in $CDCl_3$ 41
Figure S60: ^{19}F NMR spectrum of compound 5e in $CDCl_3$ 42
Figure S61: ^{1}H NMR spectrum of compound 5f in $CDCl_3$ 42
Figure S62: ^{13}C NMR spectrum of compound 5f in $CDCl_3$ 43
Figure S63: ^{19}F NMR spectrum of compound 5f in $CDCl_3$ 43
Figure S64: ^{1}H NMR spectrum of compound 5g in $CDCl_3$ 44

Figure S65: ^{13}C NMR spectrum of compound 5g in $CDCl_3$ 44
Figure S66: ^{19}F NMR spectrum of compound 5g in $CDCl_3$ 45
Figure S67: ^{1}H NMR spectrum of compound 5h in $CDCl_3$ 45
Figure S68: ^{13}C NMR spectrum of compound 5h in $CDCl_3$ 46
Figure S69: ^{19}F NMR spectrum of compound 5h in $CDCl_3$ 46
Figure S70: ^{1}H NMR spectrum of compound 6 in $CDCl_3$ 47
Figure S71: ^{13}C NMR spectrum of compound 6 in $CDCl_3$ 47

1 Synthesis and characterization

1.1. Chemicals and reagents

For synthesis, protoporphyrin IX was purchased from porphyrin-systems (Frontier scientific) (Halstenbek, Germany), Fluorinated alcohol from Fluorochem (Hadfiel, United Kingdom), and 1H,1H,2H,2H-perfluorohexanethiol and 1H,1H,2H,2H-perfluorooctanethiol were graciously provided by Atochem (Colombes, France). All reagents were from commercial sources and were used as received. All solvents were distilled and dried according to standard procedures. Reactions were checked for completions by TLC (EM Science, silica gel 60 F 254) which were visualized by quenching of u.v. fluorescence (λmax= 254 nm) or by spraying a 5% sulfuric acid solution in ethanol or a 2% ninhydrin solution in ethanol, and then by heating at ~ 150°C. Flash chromatography were performed using silica gel 60 (40-63µm, 230-400 mesh) or on combiflash Rf 200 apparatus from Teledyne Isco equipped with a UV detector. Size ex-clusion chromatography was carried out on hydroxypropylated cross-linked dextran (LH 20) from GE Healthcare. HR-MS spectra were recorded on a mass spectrometer equipped with a TOF analyzer for ESI + experiments at the Laboratoire de Mesures Physiques of University Montpellier 2 (IBMM instrument platform), NMR spectra were recorded on BRUCKER Avance 400 spectrometrer. Samples were prepared in CDCL3 (referenced to 7.26 ppm for 1H and 77.16 for 13C). Coupling constant (J) are in Hertz and corrected to the nearest 0.5 Hz. Multiplicities are reported as follows: s, singlet, d, doublet, dd, doublets of doublets, t, triplet, q, quartet, m multiplet, c, complex, and br broad pic. 1H NMR spectral assignments are supported by 1H-1H COSY and 13C-1H HSQC. Carbon spectra are supported by 13C-1H HSQC analysis where necessary. DLS experiments were performed on a Zetasizer Nano-S model 1600 (Malvern Instruments, UK) equipped with a He–Ne laser (λ= 633 nm, 4.0mW). The time-dependent correlation function of the scattered light intensity was measured at a fixed angle of 173° (backscattering detection). Each PpIX derivative was dissolved in pure Milli-Q water and centrifugated for 60 min at 25830g before being transferred into a 45 µL low-volume quartz batch cuvette (Hellma) for measurements. The intensity in kilo counts per second (Kpcs) was measured for each compound at 6 different concentrations (500, 100, 20, 4, 2, 0.5 µM). It is noteworthy that due to an overlaping between the absorbance of PpIX derivatives and the emmited light of the DLS apparatus (operating at 633 nm) the detection of the scattered light was non relevant at very low concentrations for some of them (intensity of scattered light not plotted at each concentration).

1.2 Synthesis of ***AminoPEG550 (1)***

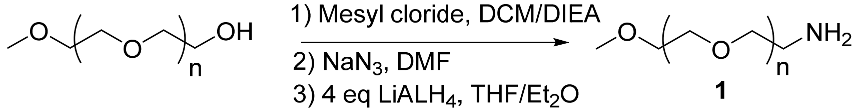

1

Synthesis of compound **1-O-Mesyl-ω-methoxy-PEG550**

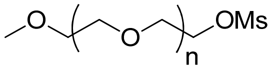

To a solution of monomethylPEG 550 (5 g, 9.0 mmol, 1 eq.) in 130 mL of DCM and 11 mL of DIEA (63 mmol, 7 eq.), cooled to 0 °C, was slowly added 3.5 mL of mesylchloride (45 mmol, 5 eq.). The solution was stirred for 16 h at room temperature *under* a *blanket* of *dry* N_2. The mesyl chloride remaining was neutralized with MeOH, then the solution was concentrated under reduced pressure and the residue was purified by column chromatography over SiO_2 (AcOEt/methanol 95:5) to give 3.5g of product as slightly orange oil (yield=62%). TLC Rf= 0.31 (Dichloromethane/ Methanol 9.5/0.5).
^{1}H NMR (400MHz, $CDCl_3$) δ 4.37 (m, 2H), 3.75 (m, 2H), 3.64 (m, 49H), 3.54 (m, 2H), 3.69 (s, 3H, **OMe**), 3.08 (s, 3H, **mesyl**).^{13}C NMR (100 MHz, $CDCl_3$) δ 72.04, 70.75–70.62 (m), 69.43, 69.14, 59.16 (**OMe**), 37.86 (**mesyl**).

Synthesis of compound **1-azide-ω-methoxy-PEG550**

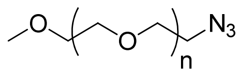

To a solution of **1-O-Mesyl-ω-methoxy-PEG550** (1.8 g, 2.86 mmol, 1 eq.) in 20 mL of DMF was added 558 mg of sodium azide (8.60 mmol, 3 eq.). The solution was stirred for 72 h at room temperature; then the reaction is concentrated under reduced pressure, dissolved in 20 ml of AcOEt and subsequently filtrated. The filtrate is concentrated under high vacuum to give 1.64g of product as slightly orange oil (yield= quantitative).
^{1}H NMR (400MHz, $CDCl_3$) δ 3.65–3.62 (m, 48H), 3.54 (m, 2H), 3.39–3.37 (m, 5H, **CH_2, OMe**).^{13}C NMR (100 MHz, $CDCl_3$) δ 72.26, 70.65, 70.13, 59.13 (**OMe**), 50.79 (**CH_2**-N_3).

Synthesis of compound **1-amino-ω-methoxy-PEG550 (1)**

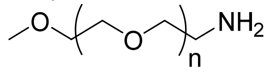

To a cold solution of 400 mg of **1-azide-ω-methoxy-PEG550** (0.69 mmol, 1 eq) in 20 ml of dry THF and diethyl ether 1/1 is added 105 mg of $LiAlH_4$ (2.77 mmol, 4eq), and the slurry is stirred during 16 hours at room temperature. Then a 10% solution of NaOH is added in a dropwise manner, then the solution is filtered over celite with a mixture of ethyl acetate and methanol, the filtrate is subsequently concentrated *in vacuo* to afford the pure compound **1** in quantitative yield.

*1.3 Synthesis of Bromo- PpIX (compound **2**)*

Typically, 334 mg of PpIX are dissolved in 35 ml of a solution of HBr in acetic acid (33%) during 24 hours, then the solvent is removed *in vacuo* to afford 590 mg of pure product in quantitative yield. We obtained protonated BrPpIX where the counter anion can be either Br^- or CH_3COO^- according the NMR spectra there is two CH_3COO^-. Mw is considered to be 1002 g/mol.

*1.4 Synthesis of tetrasubstituted PpIX derivatives (compounds **3a to 3h**)*

100 mg of Bromo- PpIX **1** are dissolved in a few ml of alcohol and the resulting mixture is stirred during 16 hours. Then the reaction mixture is diluted with EtOAc and washed twice with a saturated solution of $NaHCO_3$, dried over Na_2SO_4, dried through high vacuum to afford pure compounds in quantitative yield:

a) Synthesis of tetrasubstituted **C4PpIXC4** (compound **3a**)

100 mg of **BrPpIX** are dissolved in 5 ml of butanol and the resulting mixture is stirred during 16 hours. Then the reaction mixture is diluted with EtOAc and washed twice with a saturated solution of $NaHCO_3$, dried over Na_2SO_4 dried under high vacuum to afford 82 mg of pure **3a** in quantitative yield. ^1H NMR ($CDCl_3$, 400 MHz) δ 10.77 (1H, d, *J*=2.92Hz **CH**), 10.75, 10.20, 10.18 (3H, s, **CH**), 6.24 (2H ,m, **CH**-O), 4.52 (4H, dt, *J* =7.72, 22.49 Hz **CH_2**-CH_2-C=O), 4.22 (4H, m, ester O-**CH_2**- CH_2), 3.92-3.68 (16H, m, **CH_3**, ether O-**CH_2**-CH_2), 3.40 (4H, dt, *J* =6.91, 21.00 Hz CH_2-**CH_2**), 2.39 (6H, d, *J* =6.66Hz, **CH_3**), 1.89 (4H, m, ether O-CH_2-**CH_2**), 1.56 (6H, m, ester O-CH_2-**CH_2, CH_2**), 1.42-1.24 (6H, m, ester O-CH_2-**CH_2-CH_2**), 0.97 (6H, m, **CH_3**), 0.85 (6H, t, *J*=7.43 Hz **CH_3**), -3.69 (2H, m, **NH**); ^{13}C NMR ($CDCl_3$, 100 MHz) δ 173.45, 173.42 (C=O), 147.26-141.69, 140.61, 139.41, 138.66, 137.25, 136.65 (C pyrrole **CH_2**=CH), 98.95, 98.72, 96.86, 96.33 (CH_2=**CH**), 73.49 (**CH**-O), 69.42 (**CH_2**-Ox2), 64.65 (**CH_2**-O ester x2), 37.34 (**CH_2**-C=O), 32.57 (ether O-CH_2-**CH_2**), 30.74, 29.86 (ester O-CH_2-**CH_2**), 25.65 (**CH_3** x2), 22.09, 22.03 (**CH_2**- CH_2), 19.75, 19.14 (**CH_2**) 14.10, 13.68 (CH_2-**CH_3**), 11.94, 11.83, 11.76, 11.72 (**CH_3**). ESI Calcd for $C_{50}H_{71}N_4O_6$: 823.54 [M+H$^+$], found m/z 823.54 [M+H$^+$] HRMS Calcd for $C_{58}H_{87}N_4O_6$: 823.5374 [M+H$^+$], found m/z 823.5378 [M+H$^+$].

b) Synthesis of tetrasubstituted **C6PpIXC6** (compound **3b**)

100 mg of **BrPpIX** are dissolved in 3 ml of hexanol and the resulting mixture is stirred during 16 hours. Then the reaction mixture is diluted with EtOAc and washed twice with a saturated solution of $NaHCO_3$, dried over Na_2SO_4 dried under high vacuum to afford 94 mg of pure **3b** in quantitative yield. ^1H NMR ($CDCl_3$, 400 MHz) δ 10.71, 10.68 (1H, d, *J*=2.12Hz **CH**), 10.17, 10.16 (3H, s, **CH**), 6.17 (2H ,m, **CH**-O), 4.49 (4H, m, **CH_2**-CH_2-C=O), 4.11 (4H, m, ester O-**CH_2**-CH_2), 3.88-3.62 (16H, m, **CH_3**, ether O-**CH_2**-CH_2), 3.36 (4H, m, CH_2-**CH_2**), 2.34 (6H, d, *J* =6.66Hz, **CH_3**), 1.84 (4H, m, ether O-CH_2-**CH_2**), 1.50 (4H, m, ester O-CH_2-**CH_2**), 1.42-1.04 (24H, m, **CH_2**), 0.77 (12H, m, **CH_3**), -3.62 (2H, br, **NH**); ^{13}C NMR ($CDCl_3$, 100 MHz) δ 173.47 (C=O), 144.72-142.78, 140.43, 138.59, 137.22, 136.82 C pyrrole (**CH_2**=CH), 98.91, 98.72, 96.89, 96.39 (CH_2=**CH**), 73.58, 73.32 (**CH**-O), 69.72 (**CH_2**-Ox2), 64.97 (**CH_2**-O ester x2), 37.35 (**CH_2**-C=O), 31.89 (ether O-CH_2-**CH_2**), 31.40, 30.44 (ester O-CH_2-**CH_2**), 25.74, 25.58 (**CH_3** x2), 22.72, 22.45, 22.09 (**CH_2**) 14.56, 14.40, 14.07, 13.90 (CH_2-**CH_3**), 12.38, 12.01, 11.89, 11.84 (**CH_3**). ESI Calcd for $C_{58}H_{87}N_4O_6$: 935.66 [M+H$^+$], found m/z 935.66 [M+H$^+$] HRMS Calcd for $C_{58}H_{87}N_4O_6$: 935.6626 [M+H$^+$], found m/z 936.6633 [M+H$^+$].

c) Synthesis of tetrasubstituted **C8PpIXC8** (compound **3c**)

100 mg of **BrPpIX** are dissolved in 2 ml of octanol and the resulting mixture is stirred during 16 hours. Then the reaction mixture is diluted with EtOAc and washed twice with a saturated solution of $NaHCO_3$, dried over Na_2SO_4 dried under high vacuum to afford 105 mg of pure **3c** in quantitative yield. ^1H NMR ($CDCl_3$, 400 MHz) δ 10.71, 10.69 (1H, d, *J*=4.63Hz **CH**), 10.17, 10.16 (3H, s, **CH**), 6.19 (2H ,m, **CH**-O), 4.50 (4H, dd, *J* =7.66, 15.30 Hz **CH_2**-CH_2), 4.13 (4H, dt, *J* = 6.79, 6.79, 11.53 Hz ester O-**CH_2**-CH_2), 3.86-3.65 (16H, m, **CH_3**, ether O-**CH_2**-CH_2), 3.37 (4H, m, CH_2-**CH_2-**C=O), 2.34 (6H, dd, *J* =1.75, 6.61 Hz, **CH_3**), 1.84 (4H, m, ether O-CH_2-**CH_2**), 1.51 (4H, m, ester O-CH_2-**CH_2**), 1.43-0.98 (36H, m, **CH_2**), 0.77 (12H, m, **CH_3**) , -3.61 (2H, br, **NH**); ^{13}C NMR ($CDCl_3$, 100 MHz) δ 173.49 (C=O), 146.22-142.17, 140.55, 138.63, 137.21, 136.66 C pyrrole (**CH_2**=CH), 98.88, 98.67, 96.87, 96.38 (CH_2=**CH**), 73.57, 73.31 (**CH**-O), 69.72 (**CH_2**-Ox2), 64.97, 63.12 (**CH_2**-O ester x2), 37.34 (**CH_2**-C=O), 32.89 (ether O-CH_2-**CH_2**), 31.85, 31.73 (ether O-CH_2-**CH_2**), 30.18, 29.62, 29.34, 29.14, 28.65 (ester O-CH_2-**CH_2 CH_2**), 25.73, 25.58 (**CH_3** x2), 22.80, 22.68, 22.60, 22.08 (**CH_2**), 14.59, 14.13, 14.09, 13.65 (CH_2-**CH_3**), 12.61, 12.51, 12.37, 12.12 (**CH_3**) ESI Calcd for $C_{66}H_{103}N_4O_6$: 1047.79 [M+H$^+$], found m/z 1047.79 [M+H$^+$] HRMS Calcd for $C_{66}H_{103}N_4O_6$: 1047.7878 [M+H$^+$], found m/z 1047.7970 [M+H$^+$].

d) Synthesis of tetrasubstituted **C10PpIXC10** (compound **3d**)

46 mg of **BrPpIX** are dissolved in 0.8 ml of decanol and the resulting mixture is stirred during 16 hours. Then the reaction mixture is diluted with EtOAc and washed twice with a saturated solution of $NaHCO_3$, dried over Na_2SO_4 dried under high vacuum and the decanol remaining was removed by distillation under reduced pressure (temperature 80°C) to afford 62 mg of pure **3d** in quantitative yield. ^1H NMR ($CDCl_3$, 400 MHz) δ 10.65 (1H, s, **CH**), 10.63 (1H, d, *J*=5.48Hz **CH**), 10.13, 10.11 (2H, s, **CH**), 6.13 (2H ,m, **CH**-O), 4.45 (4H, m, **CH_2**-CH_2), 4.09 (4H, m, ester O-**CH_2**-CH_2), 3.85-3.55 (16H, m, **CH_3**, ether O-**CH_2**-CH_2), 3.32 (4H, t, *J*=7.61Hz CH_2-**CH_2-**C=O), 2.29 (6H, d, *J* =6.65 Hz, **CH_3**), 1.80 (4H, m, ether O-CH_2-

CH_2), 1.45 (4H, m, ester O-CH_2-**CH_2**), 1.38-0.94 (44H, m, **CH_2**), 0.82 (12H, m, **CH_3**), -3.67 (2H, br, **NH**); ^{13}C NMR ($CDCl_3$, 100 MHz) δ 173.45 (C=O), 145.66-136.43 (C pyrrole **CH_2**=CH), 98.92, 98.66, 96.86, 96.37 (CH_2=**CH**), 73.44 (**CH**-O), 69.72 (**CH_2**-Ox2), 64.98 (**CH_2**-O ester x2), 37.34 (**CH_2**-C=O), 32.95 (ether O-CH_2-**CH_2**), 32.05, 31.95 (ether O-CH_2-**CH_2**), 30.46-28.68 (ester O-CH_2-**CH_2** **CH_2**), 25.70, 25.66 (**CH_3** x2), 22.83, 22.76, 22.73, 22.09 (**CH_2**), 14.26, 14.24, 14.21, 14.19 (CH_2-**CH_3**), 11.98, 11.90, 11.78, 11.73 (**CH_3**) ESI Calcd for $C_{74}H_{119}N_4O_6$: 1159.91 [M+H$^+$], found m/z 1159.91 [M+H$^+$] HRMS Calcd for $C_{74}H_{119}N_4O_6$: 1159.9130 [M+H$^+$], found m/z 1159.9139 [M+H$^+$].

e) Synthesis of fluoro-tetrasubstituted **CF1PpIXCF1** (compound **3e**)
100 mg of **BrPpIX** are dissolved in 2 ml of 3,3,3-Trifluoropropan-1-ol and the resulting mixture is stirred during 16 hours. Then the reaction mixture is diluted with EtOAc and washed twice with a saturated solution of $NaHCO_3$, dried over Na_2SO_4 dried under high vacuum to afford 105 mg of pure **3e** in quantitative yield.
^{1}H NMR ($CDCl_3$, 400 MHz) δ 10.60, 10.58, 10.14, 10.09 (4H, s, **CH**), 6.19 (2H ,m, **CH**-O), 4.43 (4H, m, **CH_2**-CH_2-C=O), 4.33 (4H, m, **CH_2**-O ester), 3.99 (4H, m, ether O-**CH_2**-CH_2), 3.77-3.63 (12H, m, **CH_3**), 3.33 (4H, m, CH_2-**CH_2**-C=O), 2.61 (4H, m, CF_3-**CH_2**- CH_2 ether), 2.46-2.24 (10H, m, ester O-CH_2-**CH_2**, **CH_3**), -3.62 (2H, br, **NH**); ^{13}C NMR ($CDCl_3$, 100 MHz) δ 172.79 (C=O), 147.42-140.85, 139.44, 138.42, 137.53, 137.09 (C pyrrole **CH_2**=CH), 126.17 (qd, $J_{C,F}$= 276.9, 59.8 Hz CF_3), 98.70, 98.42, 97.14, 96.30 (CH_2=**CH**), 74.11 (dd, $J_{C,F}$= 72.32, 49.96Hz **CH**-O), 62.45 (t, $J_{C,F}$= 78.10 Hz ether **CH_2**-Ox2), 57.40 (t, $J_{C,F}$= 65.98 Hz **CH_2**-O ester x2), 36.91 (**CH_2**-C=O), 34.98 (q, $J_{C,F}$= 28.26 Hz ether **CH_2**-CF_3), 33.76-32.89 (m, ester **CH_2**-CF_3), 25.50, 25.39 (**CH_3**), 21.79 (**CH_2**-CH_2), 12.42, 11.93, 11.85, 11.69 (**CH_3**); ^{19}F NMR ($CDCl_3$, 376 MHz) -64.36 (6F, m, **CF_3**), -64.98 (6F, m, **CF_3**). ESI Calcd for $C_{46}H_{51}F_{12}N_4O_6$: 236.13 [M+H$^+$], found *m/z* 983.36 [M+H$^+$]. HRMS Calcd for $C_{13}H_{18}NO_3$: 983.3617 [M+H$^+$], found *m/z* 983.3621 [M+H$^+$].

f) Synthesis of fluoro-tetrasubstituted **CF2PpIXCF2** (compound **3f**)
100 mg of **BrPpIX** are dissolved in 2 ml of 3,3,4,4,4-Pentafluorobutan-1-ol and the resulting mixture is stirred during 48 hours. Then the reaction mixture is diluted with EtOAc and washed twice with a saturated solution of $NaHCO_3$, dried over Na_2SO_4 dried under high vacuum to afford 165 mg of pure **3f** in quantitative yield.
^{1}H NMR ($CDCl_3$, 400 MHz) δ 10.65 (1H, m, **CH**), 10.62 (1H, m, **CH**), 10.12 (1H, s, **CH**), 10.06 (1H, m, **CH**), 6.22 (2H ,m, **CH**-O), 4.54-4.15 (8 H, m, **CH_2**-O ester, **CH_2**-CH_2-C=O), 4.10 (4H, m, ether O-**CH_2**-CH_2), 3.86-3.42 (12H, m, **CH_3**,), 3.31 (4H, m, CH_2-**CH_2**-C=O), 2.62 (4H, m, CF_3-**CH_2**- CH_2 ether), 2.47-2.21 (10H, m, ester O-CH_2-**CH_2**, **CH_3**), -3.66 (2H, br, **NH**); ^{13}C NMR ($CDCl_3$, 100 MHz) δ 172.80 (C=O), 146.73-140.77, 139.44, 138.42, 137.53, 137.09 (C pyrrole **CH_2**=CH), 123.44-112.12 (4C, m, **CF_2**, **CF_3**), 98.62, 98.34, 97.16, 96.45 (CH_2=**CH**), 74.17 (m, $J_{C,F}$= 72.32, 49.96 **CH**-O), 61.01 (m, ether **CH_2**-Ox2), 56.58 (m, **CH_2**-O ester x2), 37.21, 36.88 (**CH_2**-C=O), 31.91 (m, ether **CH_2**-CF_3), 30.27 (m, ester **CH_2**-CF_3), 25.46, 25.35 (**CH_3**), 21.76 (**CH_2**-CH_2), 12.32, 11.88, 11.63, 11.47 (**CH_3**) ^{19}F NMR ($CDCl_3$, 376 MHz) -85.69--85.80 (12F, m, **CF_3**), -116.99 (4F, m, **CF_2**), -117.40 (4F, m, **CF_2**). ESI Calcd for $C_{50}H_{51}F_{20}N_4O_6$: 1183.35 [M+H$^+$], found *m/z* 1183.35 [M+H$^+$]. HRMS Calcd for $C_{50}H_{51}F_{20}N_4O_6$: 1183.3489 [M+H$^+$], found *m/z* 1183.3488 [M+H$^+$].

g) Synthesis of fluoro-tetrasubstituted **CF4PpIXCF4** (compound **3g**)
200 mg of BrPpIX are dissolved in 1.3 ml of 1H,1H,2H,2H,2H-Perfluorohexan-1-ol and the resulting mixture is stirred during 16 hours. Then the reaction mixture is diluted with EtOAc and washed twice with a saturated solution of $NaHCO_3$, dried over Na_2SO_4 dried under high vacuum to afford 190 mg of pure **3g** in 63% yield.
^{1}H NMR ($CDCl_3$, 400 MHz) δ 10.57-10.41 (2H, m, **CH**), 10.12-10.03 (2H, m, **CH**), 6.14 (2H ,m, **CH**-O), 4.36 (8 H, m, **CH_2**-O ester **CH_2**-CH_2-C=O), 4.00 (4H, m, ether O-**CH_2**-CH_2), 3.77-3.54 (12H, m, **CH_3**), 3.30 (4H, m, CH_2-**CH_2**-C=O), 2.28 (4H, m, CF_3-**CH_2**- CH_2 ether), 2.56 (4H, m, CF_3-**CH_2**- CH_2 ether), 2.41-2.14 (10H, m, **CH_3** ester O-CH_2-**CH_2**), -3.72 (2H, br, **NH**); ^{13}C NMR ($CDCl_3$, 100 MHz) δ 172.68 (C=O), 137.35, 137.06 (C pyrrole **CH_2**=CH), 98.61, 98.38, 97.02, 96.45 (CH_2=**CH**), 74.02 (**CH**-O), 61.26 (m, ether **CH_2**-Ox2), 56.58 (m, **CH_2**-O ester x2), 36.80 (**CH_2**-C=O), 31.92, 31.85 (m, ether **CH_2**-CF_3), 30.23 (m, ester **CH_2**-CF_3), 26.27, 25.29 (**CH_3**), 21.67 (**CH_2**-CH_2), 11.66, 11.53, 11.41 (**CH_3**); ^{19}F NMR ($CDCl_3$, 376 MHz) -81.18 (12F, m, **CF_3**), -114.02 (8F, m, **CF_2**), -124.66 (8F, m, **CF_2**), -126.16 (8F, m, CF_2). ESI Calcd for $C_{58}H_{50}F_{36}N_4O_6$: 1583.32

[M+H+], found *m/z* 1583.33 [M+H+]. HRMS Calcd for $C_{58}H_{50}F_{36}N_4O_6$: 1583.3234 [M+H+], found *m/z* 1583.3258 [M+H+].

h) Synthesis of fluoro-tetrasubstituted **CF6PpIXCF6** (compound **3h**)
100 mg of **BrPpIX** are dissolved in 2 ml of 1H,1H,2H,2H,2H-Perfluorooctan-1-ol and the resulting mixture is stirred during 16 hours. Then the reaction mixture is diluted with EtOAc and washed twice with a saturated solution of $NaHCO_3$, dried over Na_2SO_4 dried under high vacuum to afford 165 mg of pure **3h** in quantitative yield.
^{1}H NMR ($CDCl_3$, 400 MHz) δ 10.56 (1H, d, *J*=2.92Hz **CH**), 10.52 (1H, d, *J*=10.81Hz **CH**), 10.13, 10.11 (2H, s, **CH**), 6.14 (2H ,m, **CH**-O), 4.43 (4H, t, *J*=7.62 Hz **CH2**-CH2-C=O), 4.33 (4H, dd, *J*=6.79, 14.27 Hz **CH2**-O ester), 4.00 (4H, m, ether O-**CH2**-CH2), 3.74-3.58 (12H, m, **CH3**), 3.31 (4H, t, *J*=7.62 Hz CH2-**CH2-**C=O), 2.73 (4H, m, CF3-**CH2**- CH2 ether), 2.34-2.11(10H, m, ester O-CH2-**CH2**, **CH3**), -3.66 (2H, br, **NH**); ^{13}C NMR ($CDCl_3$, 100 MHz) δ 172.86 (C=O), 139.25, 138.44, 137.76, 137.19 C pyrrole (**CH2**=CH), 124.93 (dd, $J_{C,F}$= 276.9, 59.8 Hz CF3), 98.78, 98.43, 97.20, 96.46 (CH2=**CH**), 74.18 (**CH**-O), 61.41 (ether **CH2**-Ox2), 56.51, **CH2**-O ester x2), 36.97, 36,90 (**CH2**-C=Ox2), 31.89 (ether **CH2**-CF3), 30.32 (m, ester **CH2**-CF2), 25.47, 25.36 (**CH3**), 21.84 (**CH2**-CH2), 11.85, 11.70, 11.56, 11.54 (**CH3**) ^{19}F NMR ($CDCl_3$, 376 MHz) -80.92 (12F, m, **CF3**), -113.31 (4F, m, **CF2**), -113.89 (4F, m, **CF2**), -122.06 (8F, m, **CF2**), -123.04 (8F,br s, **CF2**), -123.79 (8F, br s, **CF2**), 126.31 (8F, br s, **CF2**). ESI Calcd for $C_{66}H_{51}F_{52}N_4O_6$: 1983.30 [M+H+], found *m/z* 1983.30 [M+H+]. HRMS Calcd for $C_{66}H_{51}F_{52}N_4O_6$: 236.2978 [M+H+], found *m/z* 1983.2969 [M+H+].

1.5 Hydrolysis of esters (saponification): Synthesis of disubstituted PpIX analogues **4a to 4f**

a) Synthesis of disubstituted C4PpIX (compound **4a**)
75 mg of **C4PpIXC4-3a** (1eq, 0.091 mmol) and 44 mg of LiOH (20 eq, 1.82 mmol) are dissolved in a mixture of THF (3ml) and water (1ml). The resulting mixture is stirred during 16 hours, the THF is removed *in vacuo*, and then 1 ml of glacial acetic acid is added to the remaining mixture and filtrated. The crude is dissolved in glacial acetic acid, diluted with DCM washed twice with a brine solution and dried over Na_2SO_4 to afford 64 mg of pure product in quantitative yield.
^{1}H NMR ($CDCl_3$, 400 MHz) δ 10.66 (1H, d, *J*=1.98Hz **CH**), 10.63, 9.99, 9.86 (3H, s, **CH**), 6.12 (2H, m, **CH**-O), 4.38 (2H, br, **CH2**-CH2-C=O), 4.13 (2H, br, **CH2**-CH2-C=O), 3.90-3.59 (12H, m, **CH3**, ether O-**CH2**-CH2), 3.45 (3H, s, **CH3**), 3.39-3.19 (4H, m, CH2-**CH2**), 2.28 (6H, m, **CH3**), 1.80 (4H, m, ether O-CH2-**CH2**), 1.45 (4H, m, **CH2**), 0.87 (6H, m, **CH3**); ^{13}C NMR ($CDCl_3$, 100 MHz) δ 180.08, 180.05 (C=O), 145.60-136.06 (C pyrrole **CH2**=CH), 99.14, 98.79, 96.88, 95.47 (CH2=**CH**), 73.45 (**CH**-O), 69.44 (**CH2**-Ox2), 37.62, 37.55 (**CH2**-C=O), 32.54 (ether O-CH2-**CH2**), 25.62, 25,57 (**CH3** x2), 22.03, 21.93 (**CH2**-CH2-C=O), 19.74 (**CH2**) 14.12, 14.09 (CH2-**CH3**), 11.80, 11.74, 11.69, 11.54 (**CH3**) ESI Calcd for $C_{42}H_{53}N_4O_6$: 709.40 [M-H+], found m/z 709.40 [M-H+] HRMS Calcd for $C_{42}H_{53}N_4O_6$: 709.3965 [M-H+], found m/z 709.3967 [M-H+].

b) Synthesis of disubstituted C6PpIX (compound **4b**)
62 mg of **C6PpIXC6-3b** (1eq, 0.066 mmol) and 32 mg of LiOH (20 eq, 1.32 mmol) are dissolved in a mixture of THF (3ml) and water (1ml). The resulting mixture is stirred during 16 hours, the THF is removed *in vacuo*, then 4 ml of a mixture of glacial acetic acid (1ml) and water (3ml) are added to the remaining mixture and filtrated, then the crude is dissolved in 50 ml of EtOAc and washed twice with HCl 0.1N, twice with a brine solution and dried over Na_2SO_4 to afford 50 mg of pure **4b** in quantitative yield.
^{1}H NMR ($CDCl_3$, 400 MHz) δ 10.67 (1H, s, **CH**), 10.64 (1H, d, *J*=2.71Hz **CH**), 10.10, (2H, s, **CH**), 6.11 (2H ,m, **CH**-O), 4.45 (4H, m, **CH2**-CH2-C=O), 3.82-3.54 (16H, m, **CH3**, ether O-**CH2**-CH2), 3.38 (4H, m, CH2-**CH2**), 2.26 (6H, m, **CH3**), 1.78 (4H, m, ether O-CH2-**CH2**), 1.50-1.07 (12H, m, **CH2**), 0.73 (6H, m, **CH3**); ^{13}C NMR ($CDCl_3$, 100 MHz) δ 179.81 (C=O), 139.60, 138.06, 136.79, (C pyrrole **CH2**=CH), 99.25, 98.89, 97.01, 95.71 (CH2=**CH**), 73.44 (**CH**-O), 69.76 (**CH2**-Ox2), 37.62 (**CH2**-C=O), 30.43 (ether O-CH2-**CH2**), 26.26 (**CH2**), 25.63 (**CH3** x2), 22.69, 22.17 (**CH2**), 14.06, 14.04 (CH2-**CH3**), 11.89, 11.77, 11.71 (**CH3**). ESI Calcd

for $C_{46}H_{61}N_4O_6$: 765.46 [M-H+], found m/z 765.46 [M-H+] HRMS Calcd for $C_{46}H_{61}N_4O_6$: 765.4591 [M-H+], found m/z 765.4591 [M-H+].

c) Synthesis of disubstituted **C8PpIX** (compound **4c**)
54 mg of **C8PpIXC8-3c** (1eq, 0.052 mmol) and 25 mg of LiOH (20 eq, 1.03 mmol) are dissolved in a mixture of THF (3ml) and water (1ml). The resulting mixture is stirred during 16 hours, the THF is removed *in vacuo*, then 4 ml of a mixture glacial acetic acid (1ml) and water (3ml) are added to the remaining mixture and filtrated, then the crude is dissolved in 50 ml of EtOAc and washed twice with HCl 0.1N, twice with a brine solution dried over Na_2SO_4 to afford 42 mg of pure **4c** in quantitative yield.
^{1}H NMR ($CDCl_3$, 400 MHz) δ 10.69, 10.66, 10.09 (4H, s, **CH**), 6.11 (2H ,m, **CH**-O), 4.39 (4H, m, **CH2**-CH2-C=O), 3.85-3.51 (16H, m, **CH3**, ether O-**CH2**-CH2), 3.34 (4H, m, CH2-**CH2**-C=O), 2.25 (6H, s, **CH3**), 1.82 (4H, m, ether O-CH2-**CH2**), 1.51-1.02 (20H, m, **CH2**), 0.74 (6H, m, **CH3**); ^{13}C NMR ($CDCl_3$, 100 MHz) δ 179.43 (C=O), 140.86, 139.43, 138.37, 136.06 (C pyrrole **CH2** =CH), 99.49, 99.05, 97.26, 96.25 (CH2 =**CH**), 73.44 (**CH**-O), 69.85 (**CH2**-Ox2), 37.43 (**CH2**-C=O), 31.86 (**CH2**), 30.47 (ether O-CH2-**CH2**), 29.85, 29.64, 29.51, 29.36, 29.35 (**CH2**), 26.59 (**CH2**), 25.59 (**CH3** x2), 22.84, 22.70, 22.68, 22.07, 22.00 (**CH2**) 14.26, 14.12 (CH2-**CH3**), 11.92, 11.81, 11.75 (**CH3**) ESI Calcd for $C_{50}H_{69}N_4O_6$: 821.52 [M-H+], found m/z 821.52 [M-H+] HRMS Calcd for $C_{46}H_{61}N_4O_6$: 821. ESI C5217 [M-H+], found m/z 821.5217 [M-H+].

d) Synthesis of disubstituted **C10PpIX** (compound **4d**)
31 mg of **C10PpIXC10-3d** (1eq, 0.027 mmol) and 12.8 mg of LiOH (20 eq, 0.53 mmol) are dissolved in a mixture of THF (3ml) and water (1ml). The resulting mixture is stirred 16 hours, the THF is removed *in vacuo*, then 4 ml of a mixture glacial acetic acid (1ml) and water (3ml) are added to the remaining mixture and filtrated, washed with water and dissolved in a mixture of AcOEt and methanol, dried *in vacuo* and purified over LH20 (MeOH/DCM 1/2) to afford 18 mg of pure **4d** in 76.0 % yield.
^{1}H NMR ($CDCl_3$, 400 MHz) δ 10.60 (2H, s, **CH**), 10.11, 10.00 (2H, s, **CH**), 6.06 (2H ,m, **CH**-O), 4.33 (4H, m, **CH2**-CH2), 3.81-3.39 (16H, m, **CH3**, ether O-**CH2**- CH2), 3.26 (4H, t, *J*=7.61Hz CH2-**CH2**-C=O), 2.20 (6H, d, *J* =6.65 Hz, **CH3**), 1.74 (4H, m, ether O-CH2-**CH2**), 1.47-0.97 (44H, m, **CH2**), 0.77 (12H, m, **CH3**); ^{13}C NMR ($CDCl_3$, 100 MHz) δ 180.01 (C=O), 145.17-134.89 (C pyrrole **CH2**=CH), 99.05, 98.65, 96.84, 95.81 (CH2=**CH**), 73.38 (**CH**-O), 69.69 (**CH2**-Ox2), 38.82, 37.86 (**CH2**-C=O), 31.90 (ether O-CH2-**CH2**), 30.70, 30.42, 29.84, 29.65, 29.62, 29.58, 29.32, 26.54, (**CH2**), 25.58 (**CH3** x2), 22.83, 22.69, 22.17 (**CH2**), 14.25, 14.14, 13.80, 14.19 (CH2-**CH3**), 11.75, 11.65 (**CH3**) ESI calcd for $C_{54}H_{77}N_4O_6$: 877.58 [M-H+], found m/z 877.58 [M-H+] HRMS Calcd for $C_{54}H_{77}N_4O_6$: 877.5843 [M-H+], found m/z 877.5839 [M-H+].

e) Synthesis of fluoro-disubstituted **CF1PpIX** (compound **4e**)
45 mg of **CF1PpIXCF1-3e** (1eq, 0.045 mmol) and 22 mg of LiOH (20 eq, 0.90 mmol) are dissolved in a mixture of THF (3ml) and water (1ml). The resulting mixture is stirred during 2H 30min, the THF is removed *in vacuo*, then 4 ml of a mixture glacial acetic acid (1ml) and water (3ml) are added to the remaining mixture and filtrated, the crude is dissolved in 30 ml of EtOH and dried under high vacuum to afford 35 mg of pure **4e** in quantitative yield.
^{1}H NMR ($CDCl_3$, 400 MHz) δ 10.54, 10.50, 9.93, 9.81 (4H, s, **CH**), 6.10 (2H ,m, **CH**-O), 4.27 (2H, br, **CH2**-CH2-C=O), 3.95 (6H, m, ether O-**CH2**-CH2 **CH2**-CH2-C=O), 3.76-3.52 (9H, m, **CH3**), 3.38 (3H, m, **CH3**), 3.30-3.06 (4H, m, CH2-**CH2**-C=O), 2.56 (4H, m, CF3-**CH2**-CH2 ether), 2.25 (6H, m, **CH3**); ^{13}C NMR ($CDCl_3$, 100 MHz) δ 179.73 (C=O), 142.22-135.43 (C pyrrole **CH2**=CH), 126.44 (q, $J_{C,F}$= 276.9 Hz CF3), 98.76, 98.41, 97.01, 95.81 (CH2 =**CH**), 74.19, 73.97 (**CH**-O), 62.45 (**CH2**-Ox2), 37.53 (**CH2**-C=O), 34.98 (q, $J_{C,F}$= 28.54 Hz ether **CH2**-CF3), 25.41, 25.34 (**CH3**) 21.95, 21.73 (**CH2**-CH2), 12.25, 11.70, 11.63, 11.43 (**CH3**) ^{19}F NMR ($CDCl_3$, 376 MHz) -64.44 (6F, m, **CF3**), ESI Calcd for $C_{40}H_{43}F_6N_4O_6$: 789.31 [M-H+], found *m/z* 789.31 [M-H+]. HRMS Calcd $C_{40}H_{43}F_6N_4O_6$: 789.3087 [M-H+], found *m/z* 789.3121 [M-H+].

f) Synthesis of fluoro-disubstituted **CF2PpIX** (compound **4f**)
74 mg of **CF2PpIXCF2-3f** (1eq, 0.063 mmol) and 30 mg of LiOH (20 eq, 1.25 mmol) are dissolved in a mixture of THF (3ml) and water (1ml). The resulting mixture is stirred during 2H 30min, the THF is removed *in vacuo*, then 4 ml of a mixture glacial acetic acid (1ml) and water (3ml) are added to the

remaining mixture and filtrated, then the crude is dissolved in 50 ml of EtOAc and washed twice with HCl 0.1 N, dried over Na_2SO_4 to afford 56 mg of pure **4f** in quantitative yield.
^{1}H NMR ($CDCl_3$, 400 MHz) δ 10.54 (1H, d, *J*=3.15Hz **CH**), 10.49, 9.97, 9.82 (3H, s, **CH**), 6.13 (2H ,m, **CH**-O), 4.28 (2 H, br, **CH_2**-O ether), 4.13-3.92 (6H, m, ether O-**CH_2**-CH_2, CH_2-**CH_2**-C=O), 3.76-3.55 (9H, m, **CH_3**), 3.49-3.11 (7H, m, **CH_3**-CH_2, **CH_2**-C=O), 2.57 (4H, m, CF_3-CF_2-**CH_2**-CH_2 ether), 2.34-2.27 (6H, m, **CH_3**); ^{13}C NMR ($CDCl_3$, 100 MHz) δ 179.46 (C=O), 144.78-133.22 (C pyrrole **CH_2**=CH), 98.72, 98.20, 96.92, 95.49 (CH_2=**CH**), 74.15, (**CH**-O), 61.47 (ether **CH_2**-Ox2), 37.45, 37.26 (**CH_2**-C=O), 31.92 (m, ether **CH_2**-CF_3), 25.43, 25.32 (**CH_3**), 21.79 (**CH_2**-CH_2), 11.64, 11.53, 11.08 (**CH_3**) ^{19}F NMR ($CDCl_3$, 376 MHz) -85.74 (6F, m, **CF_3**), -117.02 (4F, m, **CF_2**). ESI Calcd for $C_{42}H_{43}F_{10}N_4O_6$: 889.30 [M-H$^+$], found *m/z* 889.30 [M-H$^+$]. HRMS Calcd $C_{40}H_{43}F_6N_4O_6$: 889.3023 [M-H$^+$], found *m/z* 889.3018 [M-H$^+$].

g) Synthesis of fluoro-disubstituted **CF4PpIX** (compound **4g**)
100 mg of **CF4PpIXCF4-3g** (1eq, 0.063 mmol) and 30 mg of LiOH (20 eq, 1.26 mmol) are dissolved in a mixture of THF (3ml) and water (1ml). The resulting mixture is stirred during 1 hour, then the THF is removed *in vacuo*, then 4 ml of a mixture glacial acetic acid (1ml) and water (3ml) are added to the remaining mixture and filtrated, then the crude is dissolved in 50 ml of EtOAc and washed twice with HCl 0.1 N, dried over Na_2SO_4 to afford 63 mg of pure **4g** in 92.8% yield.
^{1}H NMR ($CDCl_3$, 400 MHz) δ 10.56 (1H, d, *J*=4.38Hz **CH**), 10.45 (1H, d, *J*=9.23Hz **CH**), 9.85, 9.47 (2H, s, **CH**), 6.13 (2H ,m, **CH**-O), 4.08 (8H, m, **CH_2**-O ether **CH_2**-CH_2-C=O), 3.82-3.45 (12H, m, **CH_3**), 3.15 (4H, m, CH_2-**CH_2**-C=O), 2.59 (4H, m, CF_3-**CH_2**-CH_2 ether), 2.31 (6H, m, **CH_3**); ^{13}C NMR ($CDCl_3$, 100 MHz) δ 179.54 (C=O), 139.44, 136.07 (C pyrrole **CH_2**=CH), 98.28, 97.01 (CH_2=**CH**), 74.18 (**CH**-O), 61.46 (ether **CH_2**-Ox2), 37.30 (**CH_2**-C=O), 32.08 (ether **CH_2**-CF_3), 26.08, 25.39 (**CH_3**), 22.84 (**CH_2**-CH_2), 11.69, 11.56, 11.30 (**CH_3**) ^{19}F NMR ($CDCl_3$, 376 MHz) -81.09 (12F, m, **CF_3**), -113.44 (8F, br, **CF_2**), -124.58 (8F, br, **CF_2**), -126.04(8F, m, **CF_2**). ESI Calcd for $C_{46}H_{43}F_{18}N_4O_6$: 1089.29 [M-H$^+$], found *m/z* 1089.28 [M-H$^+$]. HRMS Calcd $C_{46}H_{43}F_{18}N_4O_6$: 1089.2895 [M-H$^+$], found *m/z* 1089.2895 [M-H$^+$].

h) Synthesis of fluoro-disubstituted **CF6PpIX** (compound **4h**)
65 mg of **CF6PpIXCF6-3h** (1eq, 0.033 mmol) and 15 mg of LiOH (20 eq, 0.65 mmol) are dissolved in a mixture of THF (3ml) and water (1ml). The resulting mixture is stirred during 2h30, the THF is removed *in vacuo*, then 4 ml of a mixture glacial acetic acid (1ml) and water (3ml) are added to the remaining mixture and filtrated, then the crude is dissolved in 50 ml of EtOAc and washed twice with HCl 0.1N, dried over Na_2SO_4 to afford 40 mg of pure **4h** in 95.2 % yield.
^{1}H NMR ($CDCl_3$, 400 MHz) δ 10.56 (1H, d, *J*=3.0 Hz **CH**), 10.48 (1H, d, *J*=11.20 Hz **CH**), 10.11, 10.06 (2H, s, **CH**), 6.13 (2H ,m, **CH**-O), 4.39 (4H, m, **CH_2**-CH_2-C=O), 4.03 (4H, m, ether O-**CH_2**-CH_2), 3.72-3.49 (12H, m, **CH_3**), 3.31 (4H, m, CH_2-**CH_2**-C=O), 2.58 (4H, m, CF_3-**CH_2**-, CH_2 ether), 2.24 (6H, d, *J*=5.08 Hz **CH_3**); ^{13}C NMR ($CDCl_3$, 100 MHz) δ 179.24 (C=O), 124.93 (dd, $J_{C,F}$= 276.9, 59.8 Hz CF_3), 98.88, 97.19, 96.09 (CH_2=**CH**), 74.17 (**CH**-O), 61.42 (ether **CH_2**-Ox2), 37.53, 36.68 (**CH_2**-C=Ox2), 32.08 (ether **CH_2**-CF_3), 25.44, 25.37 (**CH_3**), 22.09 (**CH_2**-CH_2), 11.70, 11.55 (**CH_3**) ^{19}F NMR ($CDCl_3$, 376 MHz) -80.85 (6F, m, **CF_3**), -113.30 (4F, m, **CF_2**), -121.97 (4F, br s, **CF_2**), -122.97 (4F, br s, **CF_2**), -123.71 (4F, br s, **CF_2**), -126.23 (4F, br s, **CF_2**). ESI Calcd for $C_{50}H_{43}F_{26}N_4O_6$: 1289.28 [M-H$^+$], found *m/z* 1289.28 [M-H$^+$]. HRMS Calcd $C_{50}H_{43}F_{26}N_4O_6$: 1289.2767 [M-H$^+$], found *m/z* 1289.2750 [M-H$^+$].

1.6 Coupling of PEG moieties: PpIX analogues **5a to 5h & 6**

a) Synthesis of **PpIXPEG550** (compound **6**)
50 mg of **PpIX** (1eq, 0.089 mmol), 46 mg of DCC (2.5eq, 0.23 mmol) and 30 mg of HOBt (2.5eq, 0.23 mmol) were dissolved in 5 ml of DMF, after 5 min of stirring 153 mg of 1-amino-ω-methoxy-PEG550 (PEGamine) **1** (3eq, 0.27 mmol) are added, the resulting mixture is stirred during 60 hours; then the DMF is removed under high *vacuum* and the crude is purified over LH20 in DCM/MeOH 1/1 to afford 75 mg of pure **6** in 50.3% yield.
^{1}H NMR ($CDCl_3$, 400 MHz) δ 9.80, 9.76, 9.73, 9.59 (4H, s, **CH**), 8.11 (2H, m, **CH**=CH_2), 6.93 (1H, br, NH), 6.89 (1H, br, NH), 6.29 (4H, dd, *J*=5.97, 17.82Hz CH=**CH_2**), 6.14 (4H, dd, *J*=3.85, 11.37Hz CH=**CH_2**), 4.22 (4 H, br, *J*=6.57 Hz **CH_2**-CH_2-C=O), 3.74-3.17 (86H, m, **CH_2** PEG **CH_3 O-CH_3**), 3.10 (8H, m, NH-**CH_2 CH_2**-

O), 2.97 (4H, m, CH_2-**CH_2**-C=O), 2.74 (8H, m, **CH_2**-O), 2.08 (8H, m, **CH_2**-O); ^{13}C NMR ($CDCl_3$, 100 MHz) δ 172.98, 172.96 (C=O), 138.96, 136.12 (C pyrrole **CH_2**=CH), 130.25 (**CH**=CH_2), 120.62 (CH=**CH_2**), 97.37, 96.83, 96.77, 96.51 (CH_2=**CH**), 74.17 (**CH**-O), 71.99-69.07 (**CH_2** PEG), 59.07 (O-**CH_3**), 39.68 (**CH_2**-C=O), 39.14 (**CH_2**-NH), 23.01 (**CH_2**-CH_2), 12.69, 12.65, 11.55, 11.49 (**CH_3**).

b) Synthesis of amphiphilic **C4PpIXPEG550** (compound **5a**)
32 mg of **C4PpIX-4a** (1eq, 0.045 mmol), 23 mg of DCC (2.5eq, 0.113 mmol) and 15 mg of HOBt (2.5eq, 0.113 mmol) were dissolved in 3 ml of DMF, after 5 min of stirring 78 mg of PEGamine **1** (3eq, 0.27 mmol) is added, the resulting mixture is stirred during 60 hours; then the DMF is removed under high *vacuum* and the crude is purified over LH20 in DCM/MeOH 1/1 to afford 32 mg of pure **5a** in 39.0 % yield.
^{1}H NMR ($CDCl_3$, 400 MHz) δ 10.61, 10.60, 10.25, 10.10 (4H, s, **CH**), 7.04 (1H, br, NH), 6.98 (1H, br, NH), 6.11 (2H, m, **CH**-O), 4.44 (4H, m, **CH_2**-CH_2-C=O), 3.85-3.26 (101H, m, **CH_3**, ether O-**CH_2**-, **CH_2**-O), 3.26-3.08 (16H, m, **CH_2**-O, O-**CH_3**, **CH_2**-NH, CH_2-**CH_2**-C=O), 2.95 (8H, m, **CH_2**-O), 2.35 (8H, m, **CH_2**-O), 2.24 (6H, d, *J*=6.64 Hz **CH_3**), 1.78 (4H, m, ether O-CH_2-**CH_2**), 1.42 (4H, m, **CH_2**), 0.84 (6H, m, **CH_3**); ^{13}C NMR ($CDCl_3$, 100 MHz) δ 173.11 (C=O), 140.53-136.44 (C pyrrole **CH_2**=CH), 98.73, 98.54, 97.31, 96.74 (CH_2=**CH**), 73.43 (**CH**-O), 72.07-69.37 (**CH_2**-O), 59.14, 59.11 (O-**CH_3**), 39.93 (**CH_2**-C=O), 39.33 (**CH_2**-NH), 32.52 (ether O-CH_2-**CH_2**), 25.63 (**CH_3** x2), 23.26 (**CH_2**-CH_2-C=O), 19.72 (**CH_2**) 14.08 (CH_2-**CH_3**), 11.82, 11.79, 11.70 (**CH_3**).

c) Synthesis of amphiphilic **C6PpIXPEG550** (compound **5b**)
34 mg of **C6PpIX-4b** (1eq, 0.044 mmol), 23 mg of DCC (2.5eq, 0.110 mmol) and 14 mg of HOBt (2.5eq, 0.110 mmol) were dissolved in 3 ml of DMF, after 5 min of stirring 77 mg of PEGamine **1** (3eq, 0.27 mmol) are added, the resulting mixture is stirred during 24 hours; then the DMF is removed under high *vacuum* and the crude is purified over LH20 in DCM/MeOH 1/1 to afford 51 mg of pure **5b** in 62.2 % yield.
^{1}H NMR ($CDCl_3$, 400 MHz) δ 10.62, 10.59, 10.26, 10.10 (4H, s, **CH**), 7.07 (2H, br, NH), 6.11 (2H , m, **CH**-O), 4.44 (4H, m, **CH_2**-CH_2-C=O), 3.78-3.29 (93H, m, **CH_3**, O-**CH_2**, ether O-**CH_2**-CH_2), 3.27-3.07 (16H, m, **CH_2**-O, **CH_2**-NH, CH_2-**CH_2**-C=O), 2.91 (8H, m, **CH_2**-O), 2.42 (8H, m, **CH_2**-O), 2.24 (6H, m, **CH_3**), 1.77 (4H, m, ether O-CH_2-**CH_2**), 1.52-1.10 (12H, m, **CH_2**), 0.74 (6H, m, **CH_3**); ^{13}C NMR ($CDCl_3$, 100 MHz) δ 173.13 (C=O), 140.55-136.40 (C pyrrole **CH_2** =CH), 98.79, 98.51, 97.33, 96.74 (CH_2=**CH**), 73.41 (**CH**-O), 72.04-69.38 (**CH_2**-O), 59.11 (O-**CH_3**), 39.95 (**CH_2**-C=O), 39.35 (**CH_2**-NH), 31.87, 31.85 (**CH_2**), 30.42 (ether O-CH_2-**CH_2**), 26.26 (**CH_2**), 25.66, 25.63 (**CH_3** x2), 23.30, 22.68 (**CH_2**) 14.07, 14.04 (CH_2-**CH_3**), 11.86, 11.80, 11.76, 11.69 (**CH_3**) ESI Calcd for $C_{13}H_{18}NO_3$: 236.13 [M+H$^+$], found *m/z* 236.13 [M+H$^+$].

d) Synthesis of amphiphilic **C8PpIXPEG550** (compound **5c**)
25 mg of C8PpIX-**4c** (1eq, 0.032 mmol), 23 mg of DCC (2.5eq, 0.075 mmol) and 14 mg of HOBt (2.5eq, 0.075 mmol) were dissolved in 3 ml of DMF, after 5 min of stirring 57 mg of PEGamine **1** (3eq, 0.099 mmol) are added, the resulting mixture is stirred during 24 hours; then the DMF is removed under high vacuum and the crude is purified over LH20 in DCM/MeOH 1/1 to afford 20 mg of pure **5c** in 32.2 % yield.
^{1}H NMR ($CDCl_3$, 400 MHz) δ 10.62, 10.60, 10.25, 10.10 (4H, s, **CH**), 7.01 (1H, br, **NH**), 6.10 (2H ,m, **CH**-O), 4.44 (4H, m, **CH_2**-CH_2-C=O), 3.86-3.30 (87H, m, **CH_3**, O-**CH_2**, ether O-**CH_2**-CH_2), 3.28-3.18 (16H, m, **CH_2**-O, **CH_3**-O **CH_2**-NH, CH_2-**CH_2**-C=O), 2.92 (8H, m, **CH_2**-O), 2.44 (6H, m, **CH_2**-O), 2.24 (6H, d, *J*=6.34 Hz **CH_3**), 1.77 (4H, m, ether O-CH_2-**CH_2**), 1.51-1.02 (20H, m, **CH_2**), 0.76 (6H, m, **CH_3**); ^{13}C NMR ($CDCl_3$, 100 MHz) δ 173.14 (C=O), 144.71-137.04 (C pyrrole **CH_2** =CH), 98.83, 98.59, 97.35, 96.79 (CH_2=**CH**), 73.43 (**CH**-O), 72.06-69.41 (**CH_2**-O), 59.13 (O-**CH_3**), 39.94, (**CH_2**-C=O), 39.34 (**CH_2**-NH), 31.86, 31.03, 30.46 (**CH_2**), 30.46 (ether O-CH_2-**CH_2**), 29.83, 29.63, 29.49, 29.34 (**CH_2**), 26.59 (**CH_2**), 25.65 (**CH_3** x2), 23.30, 22.82, 22.70 (**CH_2**) 14.24, 14.11 (CH_2-**CH_3**), 11.87, 11.77, 11.71 (**CH_3**).

e) Synthesis of amphiphilic **C10PpIXPEG550** (compound **5d**)
9 mg of **C10PpIX-4d** (1eq, 0.010 mmol), 5 mg of DCC (2.5eq, 0.026 mmol) and 3.5 mg of HOBt (2.5eq, 0.026 mmol) were dissolved in 1 ml of DMF, after 5 min of stirring, a solution of 18 mg of PEGamine **41** (3eq, 0.065 mmol) in 2 ml of DMF is added, the resulting mixture is stirred during 24 hours; then the

DMF is removed under high *vacuum* and the crude is purified over LH20 in DCM/MeOH 2/1 to afford 13 mg of pure **5d** in 65.0 % yield.

^{1}H NMR ($CDCl_3$, 400 MHz) δ 10.61 (1H, d, *J*=3.10 Hz), 10.59 (1H, d, *J*=2.15 Hz), 10.26, 10.10 (2H, s, **CH**), 7.05 (2H, br, NH), 6.10 (2H , d, *J*=6.54 Hz **CH**-O), 4.44 (4H, m, **CH₂**-CH₂-C=O), 3.82-3.28 (77H, m, **CH₃** O-**CH₃** ether O-**CH₂**- CH₂), 3.27-3.06 (14H, m, **CH₂**-O **CH₂**-NH CH₂-**CH₂**-C=O), 2.93 (8H, m, **CH₂**-O), 2.35 (8H, m, **CH₂**-O), 2.24 (6H, d, *J*=6.54 Hz **CH₃**), 1.78 (4H, m, ether O-CH₂-**CH₂**), 1.51-1.01 (28H, m, **CH₂**), 0.79 (6H, m, **CH₃**); ^{13}C NMR ($CDCl_3$, 100 MHz) δ 173.15 (C=O), 98.75, 98.52, 97.35, 96.77 (CH₂=**CH**), 73.42 (**CH**-O), 72.05-69.38 (**CH₂**-O), 59.15 (O-**CH₃**), 39.98 (**CH₂**-C=O), 39.32 (**CH₂**-NH), 31.94 (**CH₂**), 30.46 (ether O-CH₂-**CH₂**), 39.63, 29.39, 29.36, 26.60 (**CH₂**), 25.69 (**CH₃** x2), 23.33, 22.73 (**CH₂**) 14.20 (CH₂-**CH₃**), 11.90, 11.84, 11.79, 11.73 (**CH₃**) ESI Calcd for $C_{13}H_{18}NO_3$: 236.13 [M+H$^+$], found *m/z* 236.13 [M+H$^+$].

f) Synthesis of amphiphilic **CF1PpIXPEG550** (compound **5e**)

30 mg of **CF1PpIX-4e** (1eq, 0.038 mmol), 17 mg of DCC (2.2eq, 0.083 mmol) and 11 mg of HOBt (2.2eq, 0.083 mmol) were dissolved in 3 ml of DMF, after 5 min of stirring 65 mg of PEGamine **41** (3eq, 0.114 mmol) are added, the resulting mixture is stirred during 24 hours; then the DMF is removed under high vacuum and the crude is purified over LH20 in DCM/MeOH 1/1 to afford 47 mg of pure **5e** in 65.3 % yield.

^{1}H NMR ($CDCl_3$, 400 MHz) δ 10.53, 10.51, 10.27, 10.11 (4H, s, **CH**), 7.15 (1H, br, **NH**), 7.05 (1H , br, **NH**), 6.13 (2H , q, *J*=6.42 Hz **CH**-O), 4.44 (4H, m, **CH₂**-CH₂-C=O), 3.95 (4H, m, ether O-**CH₂**-CH₂), 3.77-3.28 (95H, m, **CH₂**-O **CH₃** O-**CH₃**), 3.27-3.10 (16H, m, **CH₂**-O **CH₂**-NH CH₂-**CH₂**-C=O), 2.93 (8H, m, **CH₂**-O), 2.58 (4H, m, CF₃-**CH₂**-CH₂ ether), 2.41 (6H, m, **CH₂**-O), 2.28 (6H, d, J=6.55 Hz **CH₃**); ^{13}C NMR ($CDCl_3$, 100 MHz) δ 173.08 (C=O), 139.90-136.21 (C pyrrole **CH₂** =CH), 126.38 (q, $J_{C,F}$= 278.4 Hz CF₃), 98.44, 98.21, 97.54, 96.95 (CH₂=**CH**), 74.08 (**CH**-O), 72.02-69.37 (**CH₂**-O), 62.45 (ether O-**CH₂**-CH₂ x2), 59.09 (O-**CH₃**), 39.87, 39.81 (**CH₂**-C=O), 39.32 (**CH₂**-NH), 34.99 (q, $J_{C,F}$=28.39 Hz ether **CH₂**-CF₃), 25.43 (**CH₃**), 23.25, 23.18 (**CH₂**-CH₂-C=O), 11.76, 11.71, 11.67, 11.60 (**CH₃**) ^{19}F NMR ($CDCl_3$, 376 MHz) -64.43 (6F, m, **CF₃**).

g) Synthesis of amphiphilic **CF2PpIXPEG550** (compound **5f**)

25 mg of **CF2PpIX-4f** (1eq, 0.028 mmol), 14 mg of DCC (2.5eq, 0.070 mmol) and 11 mg of HOBt (2.5eq, 0.070 mmol) were dissolved in 3 ml of DMF, after 5 min of stirring 65 mg of PEGamine **1** (3eq, 0.084 mmol) are added, the resulting mixture is stirred during 60 hours; then the DMF is removed under high *vacuum* and the crude is purified over LH20 in DCM/MeOH 1/1 to afford 23 mg of pure **5f** in 41.0 % yield.

^{1}H NMR ($CDCl_3$, 400 MHz) δ 10.53 (1H, d, *J*=3.04 Hz **CH**), 10.50 (1H, d, *J*=3.84 Hz **CH**), 10.28, 10.11 (2H, s, **CH**), 7.08 (1H, br, NH), 6.99 (1H, br, NH), 6.14 (2H ,m, **CH**-O), 4.43 (2 H, br, **CH₂**-O ether), 4.02 (4H, m, ether O-**CH₂**-CH₂), 3.78-2.82 (116H, m, **CH₂** –O, O-**CH₃**, **CH₃**, CH₂-**CH₂**-C=O), 2.46 (4H, m, CF₃-**CH₂**-CH₂ ether), 2.27 (6H, m, *J*=2.27 Hz **CH₃**); ^{13}C NMR ($CDCl_3$, 100 MHz) δ 173.10 (C=O), 139.84-136.97 (C pyrrole **CH₂** =CH), 120.57, 117.10, 115.49 (m, CF₂, CF₃), 98.45, 98.12, 97.63, 97.03 (CH₂=**CH**), 74.17 (**CH**-O), 72.06-69.44 (**CH₂** PEG), 61.51 (ether **CH₂**-O x2), 59.13 (O-**CH₃**), 39.94 (**CH₂**-C=O), 39.37 (**CH₂**-NH), 31.93 (t, *J*=20.96 Hz ether **CH₂**-CF₃), 25.41(**CH₃**), 23.31, 23.25 (**CH₂**-CH₂), 11.81, 11.73, 11.63 (**CH₃**) ^{19}F NMR ($CDCl_3$, 376 MHz) -85.74 (6F, m, **CF₃**), -117.03 (4F, m, **CF₂**).

h) Synthesis of amphiphilic **CF4PpIXPEG550** (compound **5g**)

40 mg of **CF4PpIX-4g** (1eq, 0.037 mmol), 19 mg of DCC (2.5eq, 0.092 mmol) and 12 mg of HOBt (2.5eq, 0.092 mmol) were dissolved in 3 ml of DMF, after 5 min of stirring 64 mg of PEGamine **1** (3eq, 0.110 mmol) is added, the resulting mixture is stirred during 60 hours; then the DMF is removed under high vacuum and the crude is purified over LH20 in DCM/MeOH 1/1 to afford 38 mg of pure **5g** in 46.9 % yield.

^{1}H NMR ($CDCl_3$, 400 MHz) δ 10.59-10.42 (2H, s, **CH**), 10.29-9.99 (2H, s, **CH**), 6.15 (2H ,m, **CH**-O), 4.39 (8 H, m, **CH₂**-CH₂-C=O), 4.01 (4H, m, ether O-**CH₂**-CH₂), 3.76-3.28 (110H, m, **CH₂**-O, **CH₃**, O-**CH₃**), 3.28-3.18 (16H, m, **CH₂**-O, **CH₂**-NH, CH₂-**CH₂**-C=O), 2.58 (4H, m, CF₃-**CH₂**- CH₂ ether), 2.41 (6H, m, **CH₂**-O), 2.28 (6H, m, **CH₃**); ^{13}C NMR ($CDCl_3$, 100 MHz) δ 173.09 (C=O), 140.69-136-82 (C pyrrole **CH₂** =CH), 98.52, 98.09, 97.58, 97.00 (CH₂=**CH**), 74.19 (**CH**-O), 72.05-69.18 (**CH₂**-O), 61.43 (ether O-**CH₂**-CH₂ x2), 59.11 (O-**CH₃**), 39.87 (**CH₂**-C=O), 39.37 (**CH₂**-NH), 32.00 (m, ether **CH₂**-CF₂), 26.57, 25.47 (**CH₃**), 22.81 (**CH₂**-CH₂),

11.77, 11.70, 11.55 (**CH_3**) ^{19}F NMR ($CDCl_3$, 376 MHz) -81.08 (6F, m, **CF_3**), -113.44 (4F, br, **CF_2**), -124.57 (4F, br, **CF_2**), -126.01(4F, m, **CF_2**).

i) Synthesis of amphiphilic **CF6PpIXPEG550** (compound **5h**)
28 mg of **CF6PpIX-4h** (1eq, 0.021 mmol), 11 mg of DCC (2.5eq, 0.053 mmol) and 7 mg of HOBt (2.5eq, 0.053 mmol) were dissolved in 3 ml of DMF, after 5 min of stirring 38 mg of PEGamine **1** (3eq, 0.065 mmol) are added, the resulting mixture is stirred during 60 hours; then the DMF is removed under high *vacuum* and the crude is purified over LH20 in DCM/MeOH 1/1 to afford 19 mg of pure **5h** in 38.0 % yield.
^{1}H NMR ($CDCl_3$, 400 MHz) δ 10.55 (1H, d, *J*=3.10 Hz), 10.48 (1H, d, *J*=14.26 Hz), 10.28, 10.12 (2H, s, **CH**), 6.14 (2H ,m, **CH**-O), 4.44 (4H, m, **CH_2**-CH_2-C=O), 4.02 (4H, m, ether O-**CH_2**-CH_2), 3.74-3.29 (71H, m, **CH_2**-O, **CH_3**, O-**CH_3**), 3.28-3.10 (10H, m, **CH_2**-O, **CH_2**-NH, CH_2-**CH_2**-C=O), 2.97 (6H, m, **CH_2-O)**, 2.55 (4H, m, CF_3-**CH_2**-CH_2 ether), 2.47 (6H, m, **CH_2-O)**, 2.27 (6H, m, **CH_3**); ^{13}C NMR ($CDCl_3$, 100 MHz) δ 173.11 (C=O), 140.25-137.01 (C pyrrole (**CH_2**=CH), 98.55, 98.16, 97.64, 97.04 (CH_2=**CH**), 74.21 (**CH**-O), 72.07-69.72 (**CH_2**-O), 61.44 (ether O-**CH_2**-CH_2), 59.15 (O-**CH_3**), 39.91 (**CH_2**-C=O), 39.37 (**CH_2**-NH), 32.08 (ether **CH_2**-CF_3), 25.52, 25.44 (**CH_3**), 22.84 (**CH_2**-CH_2), 11.84, 11.73, 11.56 (**CH_3**) ^{19}F NMR ($CDCl_3$, 376 MHz) -80.83 (6F, m, **CF_3**), -113.29 (4F, m, **CF_2**), -121.95 (4F, br s, **CF_2**), -122.96 (4F,br s, **CF_2**), -123.67 (4F, br s, **CF_2**), -126.20 (4F, m, **CF_2**).

2 DLS measurements

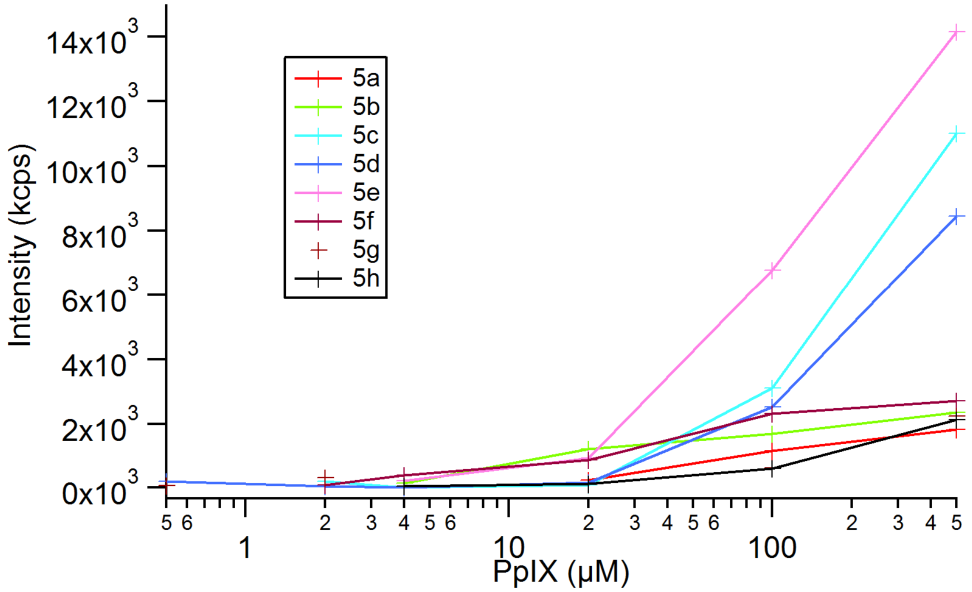

Figure S1: Intensity of scattered light (kcps) as a function of PpIX derivative concentration (µM). Intensity plotted for concentrations ranging from 500 µM to 0.5 µM.

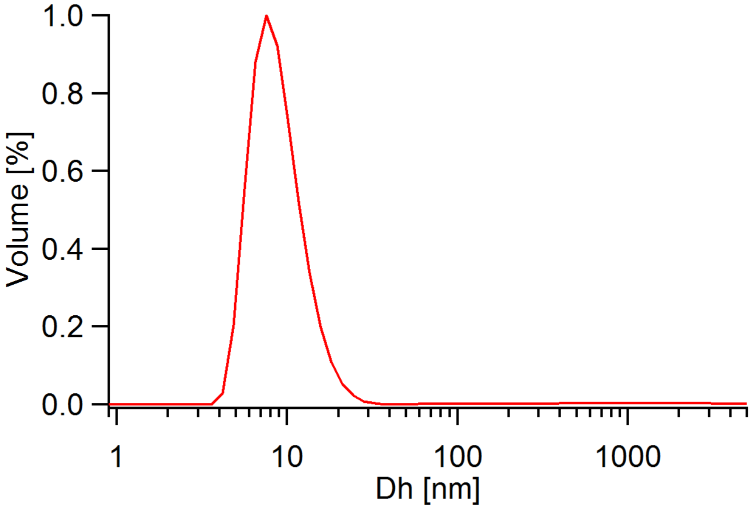

Figure S2 : Example of size distribution (DLS analysis in volume-weighted distribution CONTIN) of compound **5d** at 500 μM (at this concentration the compound is self-assembled within micelles).

3. Photochemical properties

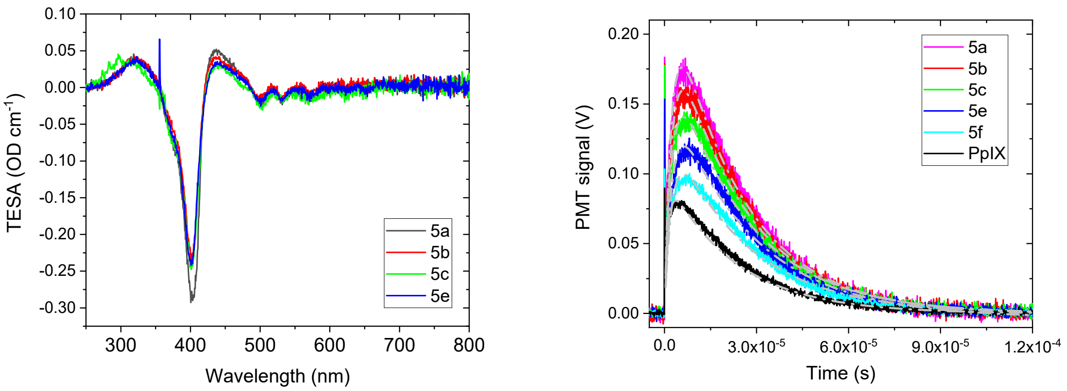

Figure S3: *Left panel*: Representative transient triplet excited state absorption (TESA) spectra of selected compounds in oxygen evacuated/depleted THF solutions recorded using 355 nm excitation. TESA is shown as a positive amplitude but ground state depletion is shown as a negative ground state absorption spectrum in each case (see Glimsdal et al [1] for details how to interpret these spectra). *Right panel*: Transient singlet oxygen luminescence at 1275 nm for excitation at 425 nm of air-saturated THF solutions. The amplitude scale is here taking into account the absorbance of the ground state absorption at 425 nm. The gray curves are fits to parameters outlined in eq (1) and giving relative singlet oxygen yields, see Table S1

Table S1: Results of fits to transient singlet oxygen luminescence using parameters defined in the eq (1), where $\mathbf{k_{SO}} = \mathbf{1/\tau_{SO}}$ and $\mathbf{k_{PS}} = \mathbf{1/\tau_{PS}}$. The resulting relative quantum efficiency (RQE) from C in eq (1) is normalized to give 1.0 for PpIX.

Parameter / *Compound*	τ_{SO} (ms)	τ_{PS} (ms)	RQE
5a	23.0±0.047	2.21±0.015	1.3
5b	23.3±0.052	2.36±0.016	1.1
5c	24.4±0.056	2.37±0.018	1.1
5e	26.0±0.014	2.35±0.015	0.95
5f	25.3±0.060	2.34±0.017	1.1
PpIX	22.8±0.080	1.17±0.013	1.0

4. NMR Spectra

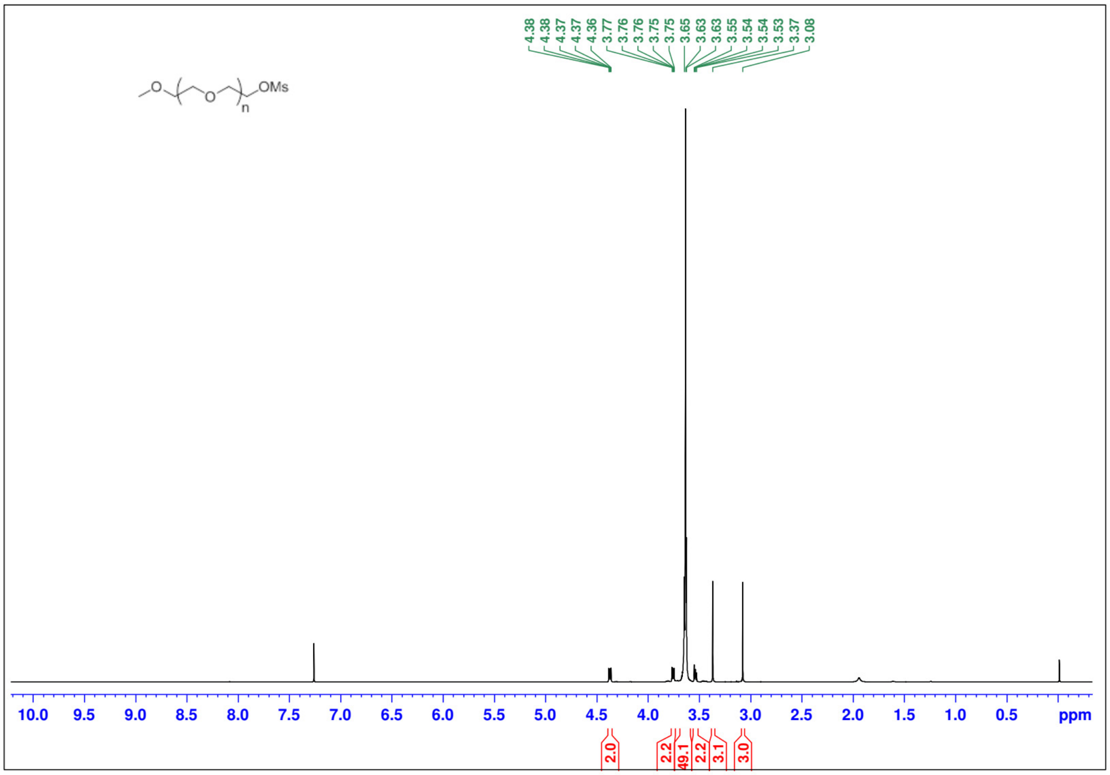

Figure S4: Figure S3: ^{1}H NMR spectrum of compound PEG-OMs in $CDCl_3$

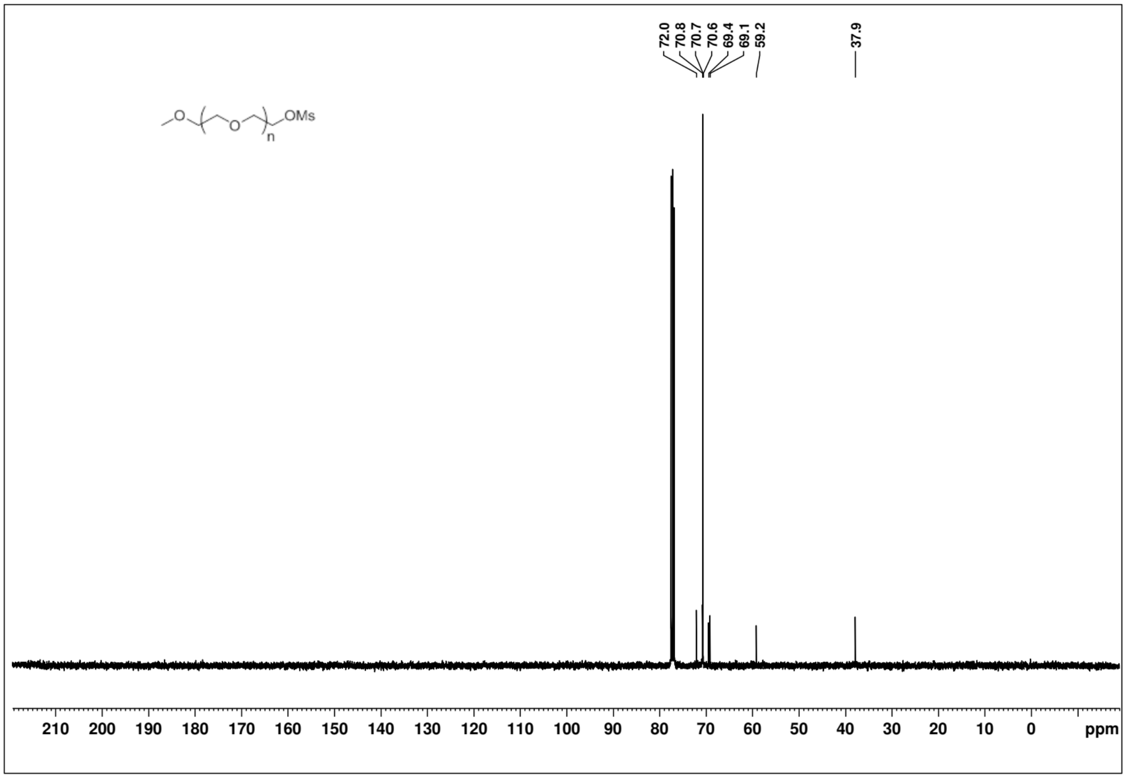

Figure S5: ^{13}C NMR spectrum of compound PEG-OMs in $CDCl_3$

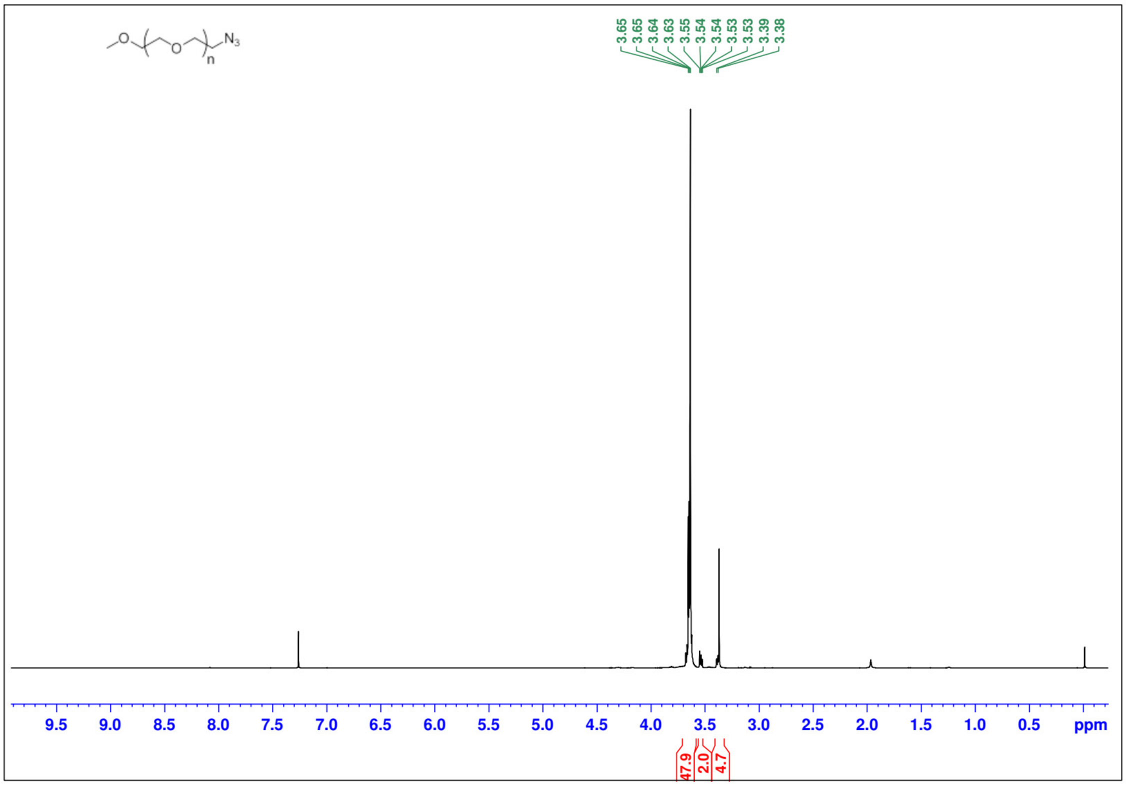

Figure S6: ^{1}H NMR spectrum of compound PEG-N3 in $CDCl_3$

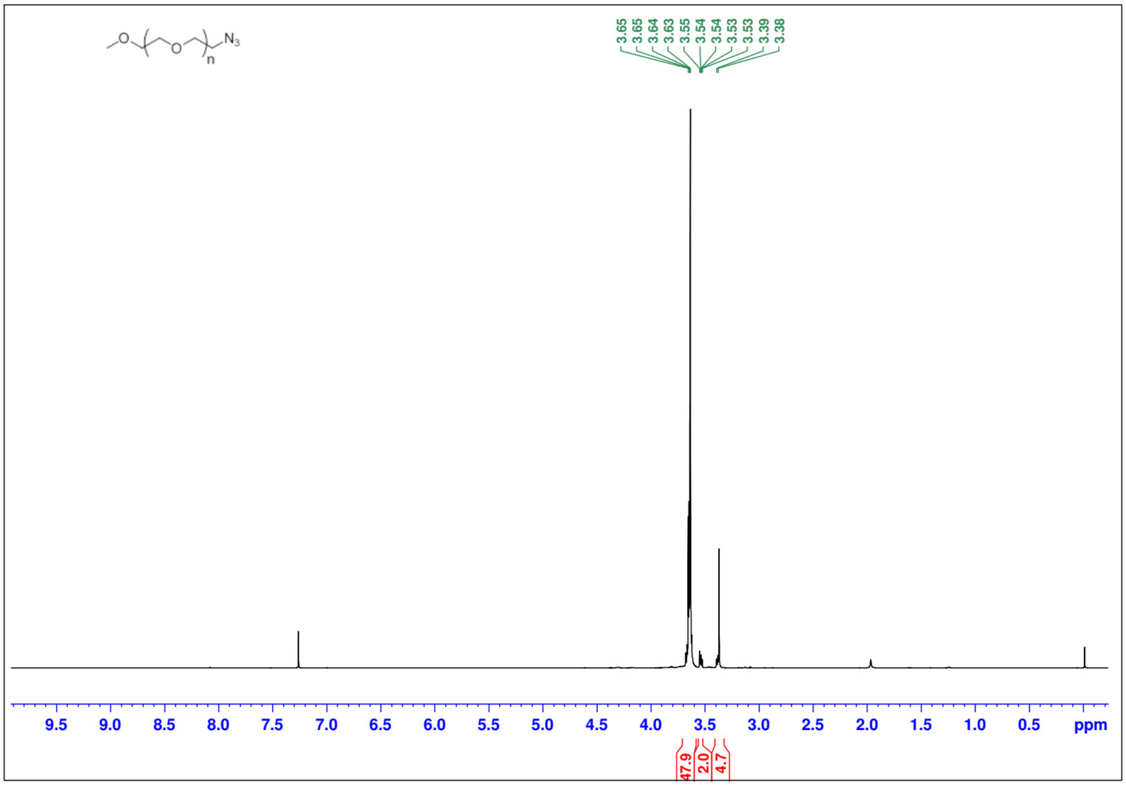

Figure S7: ^{13}C NMR spectrum of compound PEG-N3 in $CDCl_3$

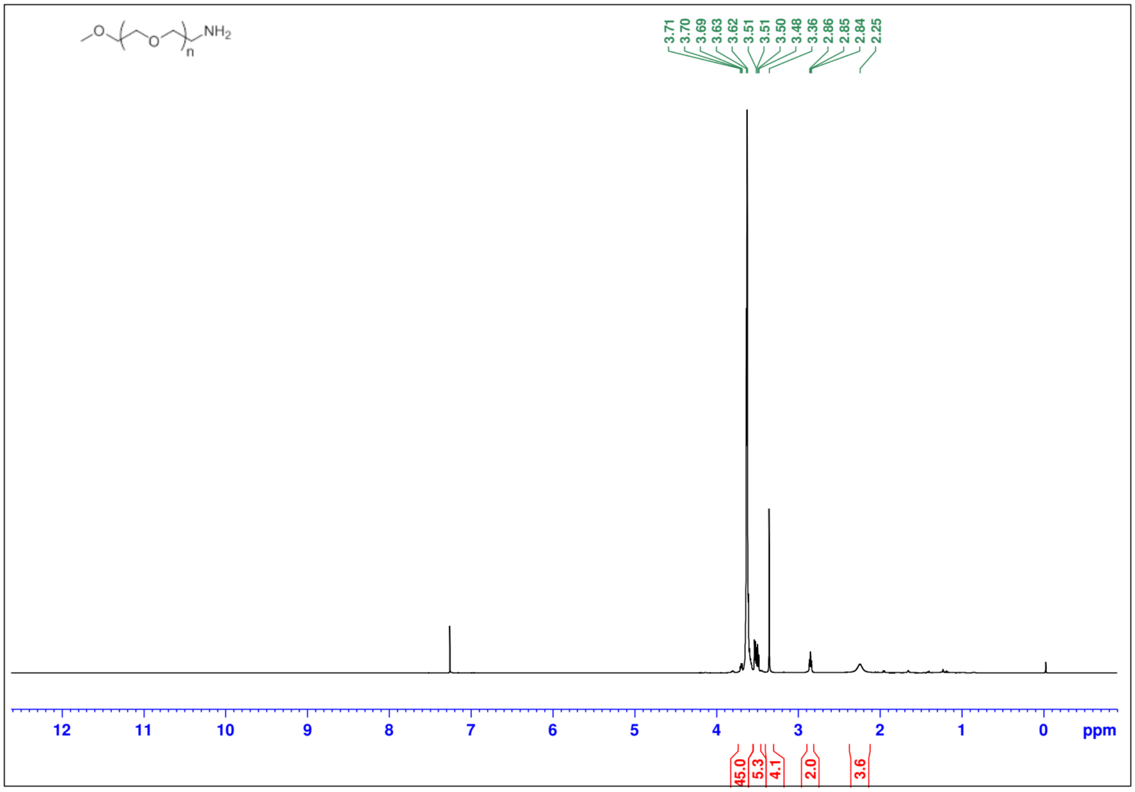

Figure S8: ^{1}H NMR spectrum of compound 1 in $CDCl_3$

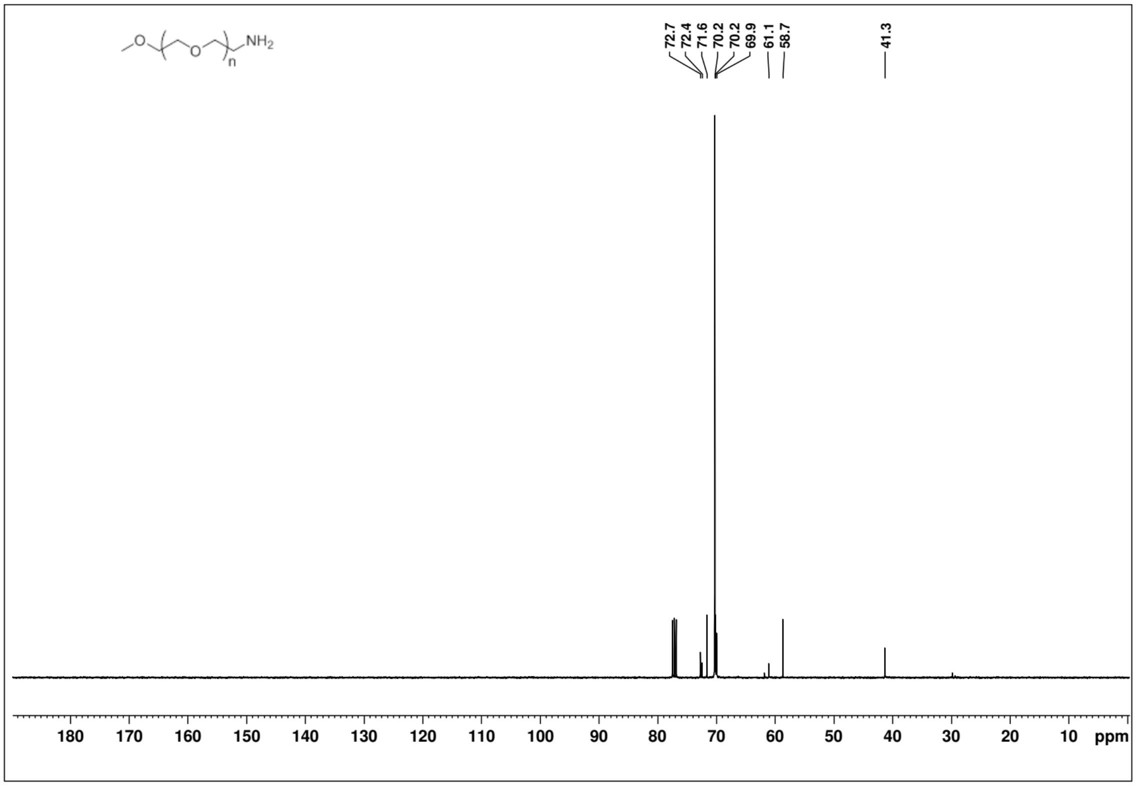

Figure S9: ^{13}C NMR spectrum of compound 1 in $CDCl_3$

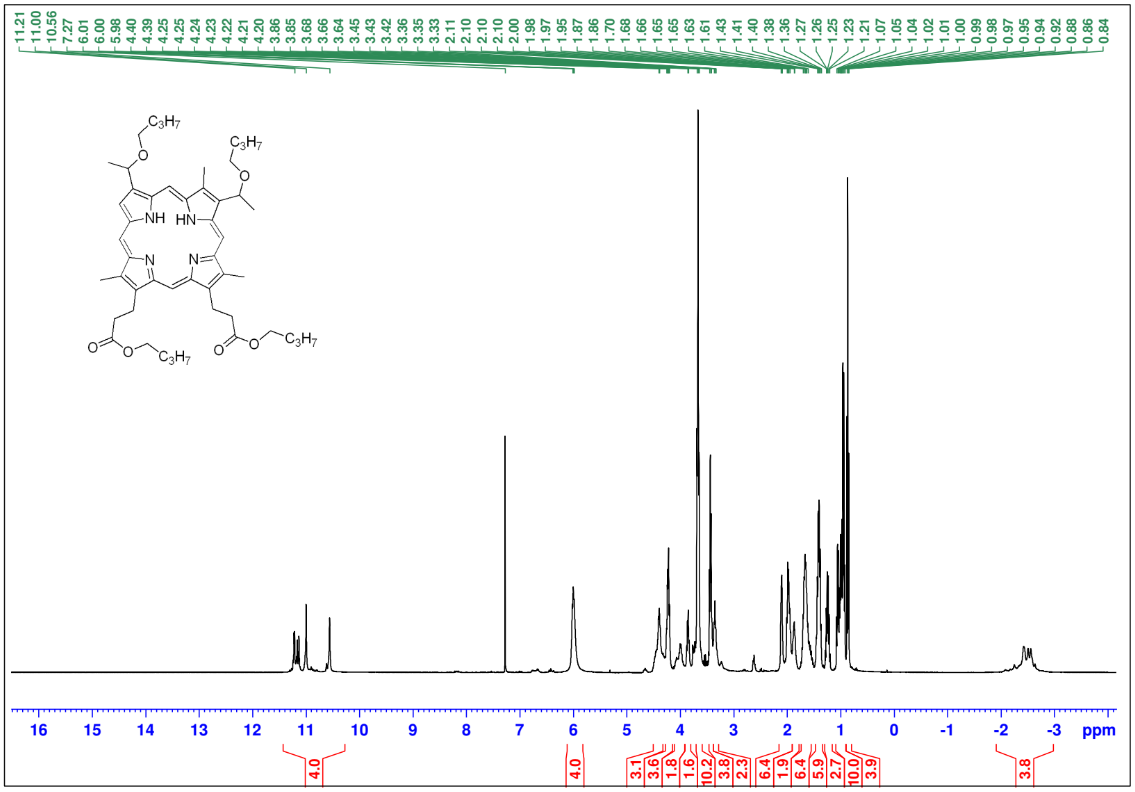

Figure S10: ^{1}**H NMR spectrum of compound 3a in CDCl**$_3$

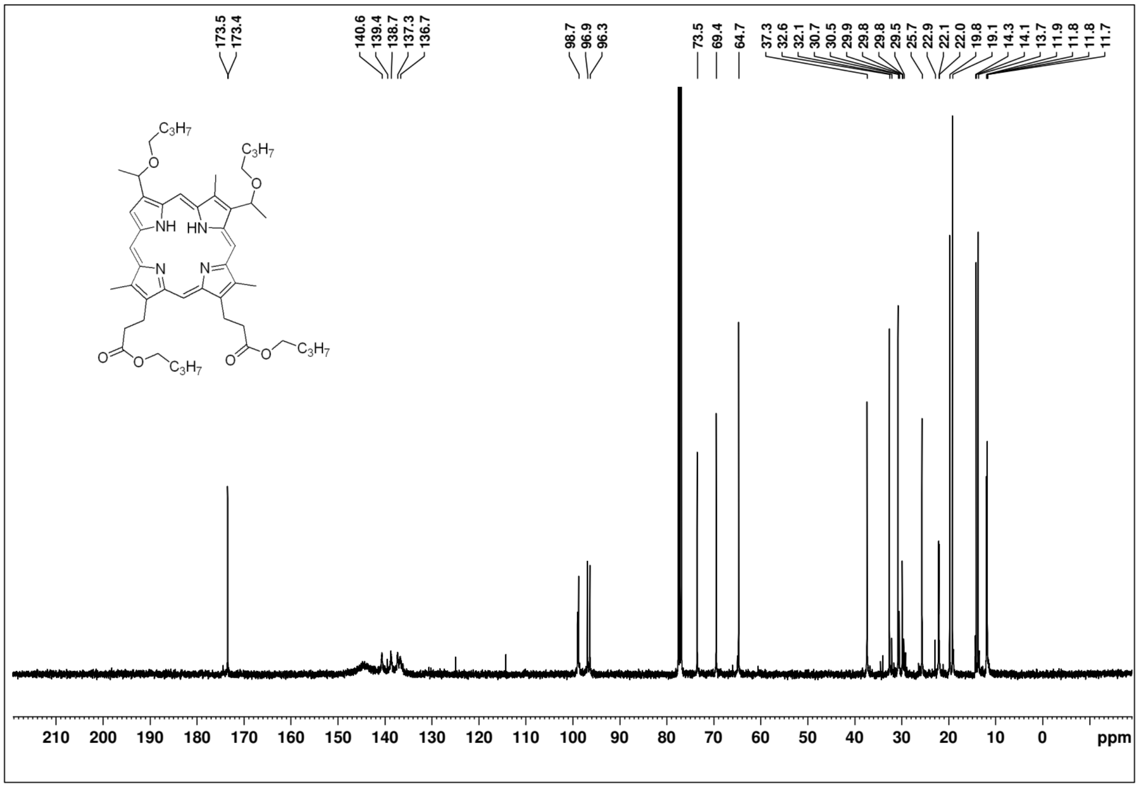

Figure S11: ^{13}**C NMR spectrum of compound 3a in CDCl**$_3$

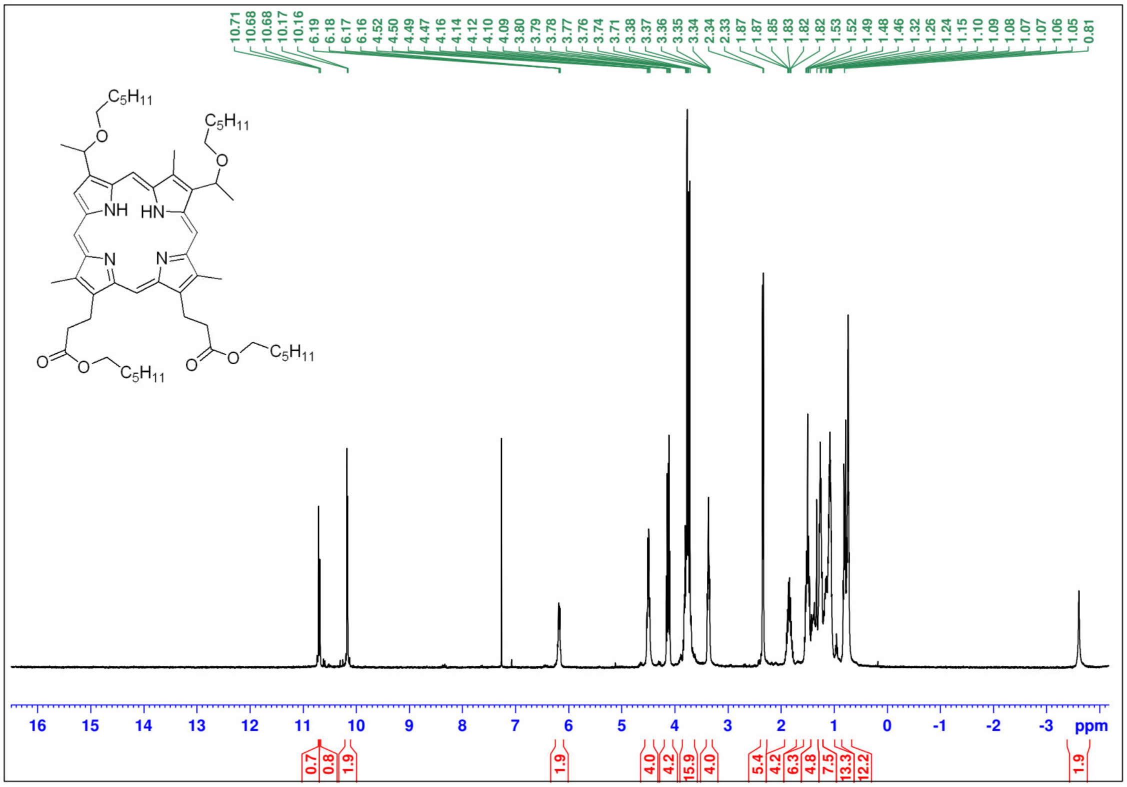

Figure S12: ^{1}H NMR spectrum of compound 3b in $CDCl_3$

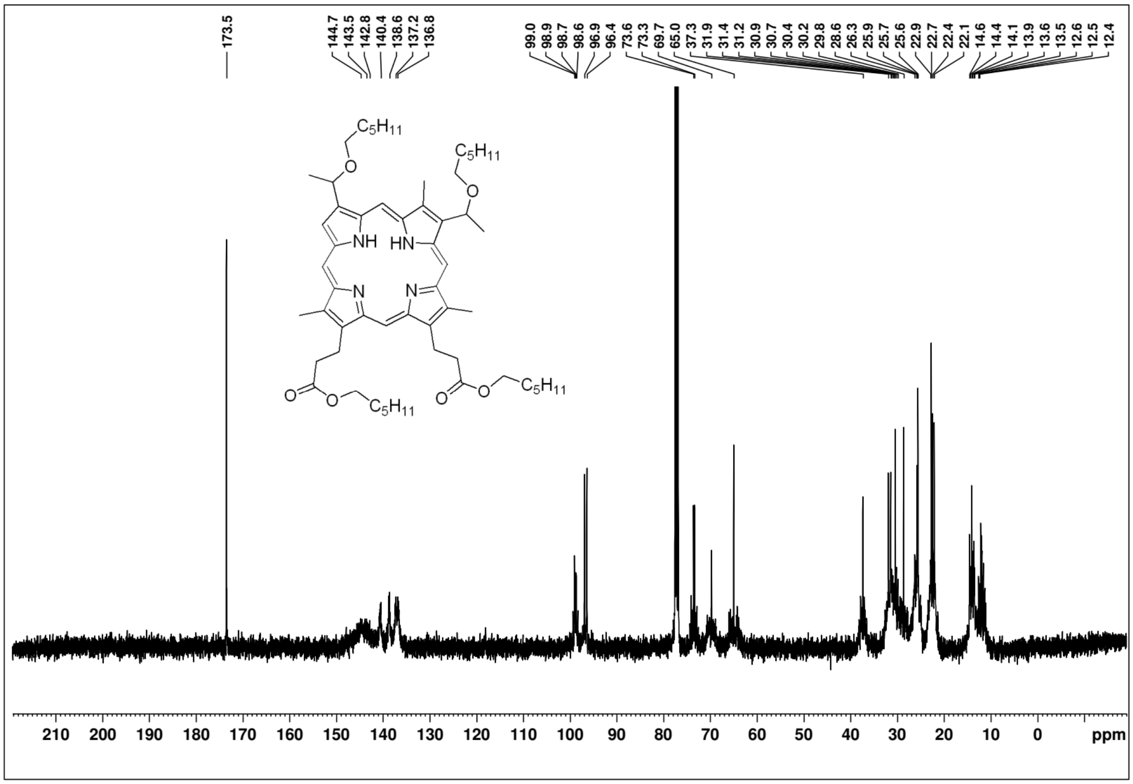

Figure S13: ^{13}C NMR spectrum of compound 3b in $CDCl_3$

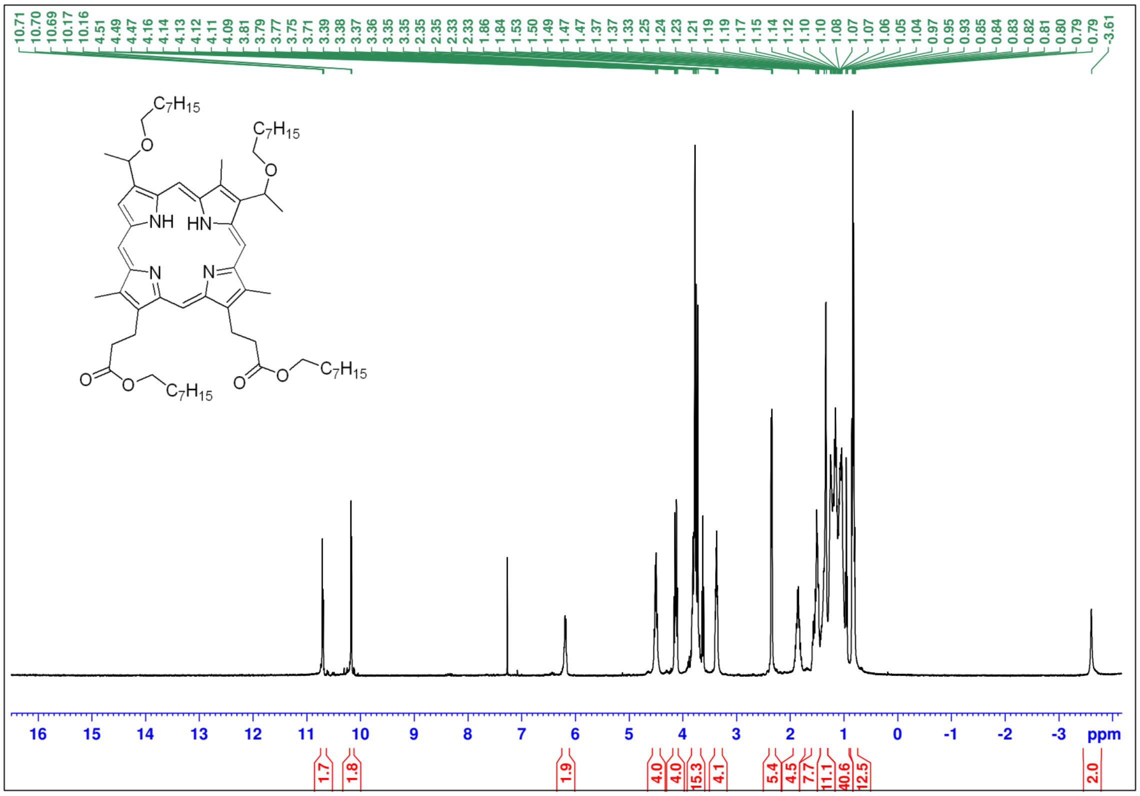

Figure S14: ^{1}H **NMR spectrum of compound 3c in** $CDCl_3$

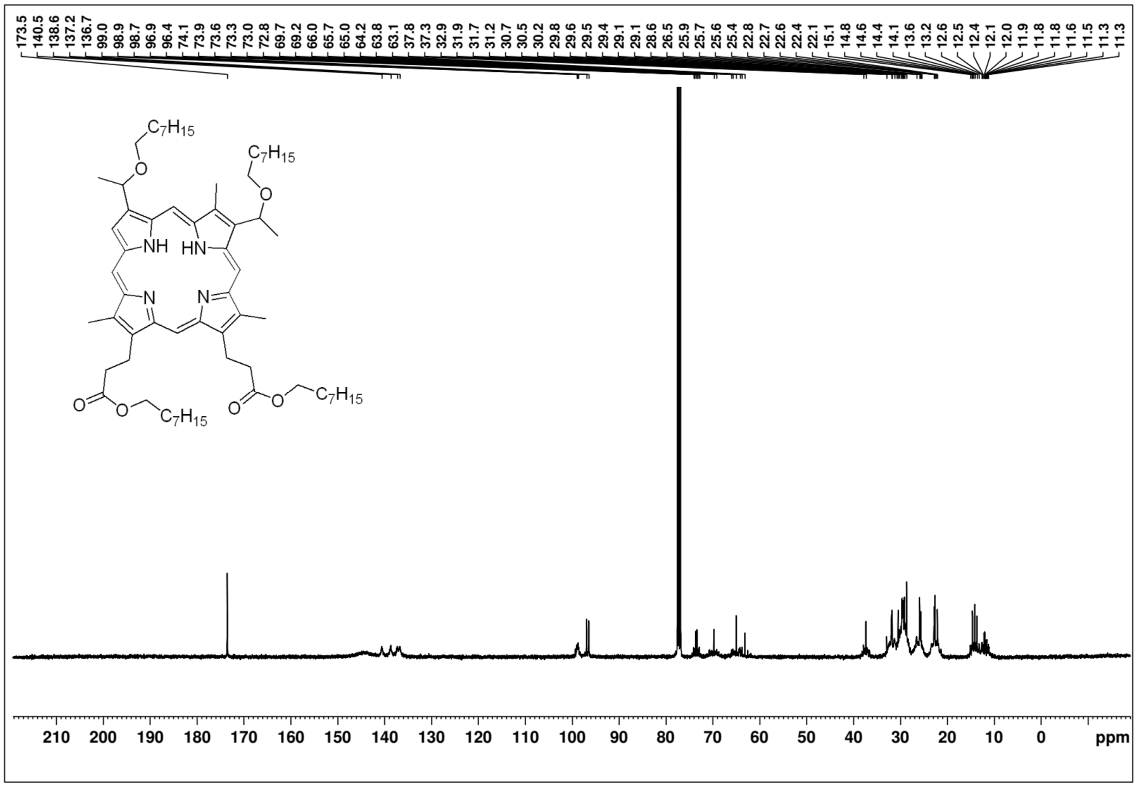

Figure S15: ^{13}C **NMR spectrum of compound 3c in** $CDCl_3$

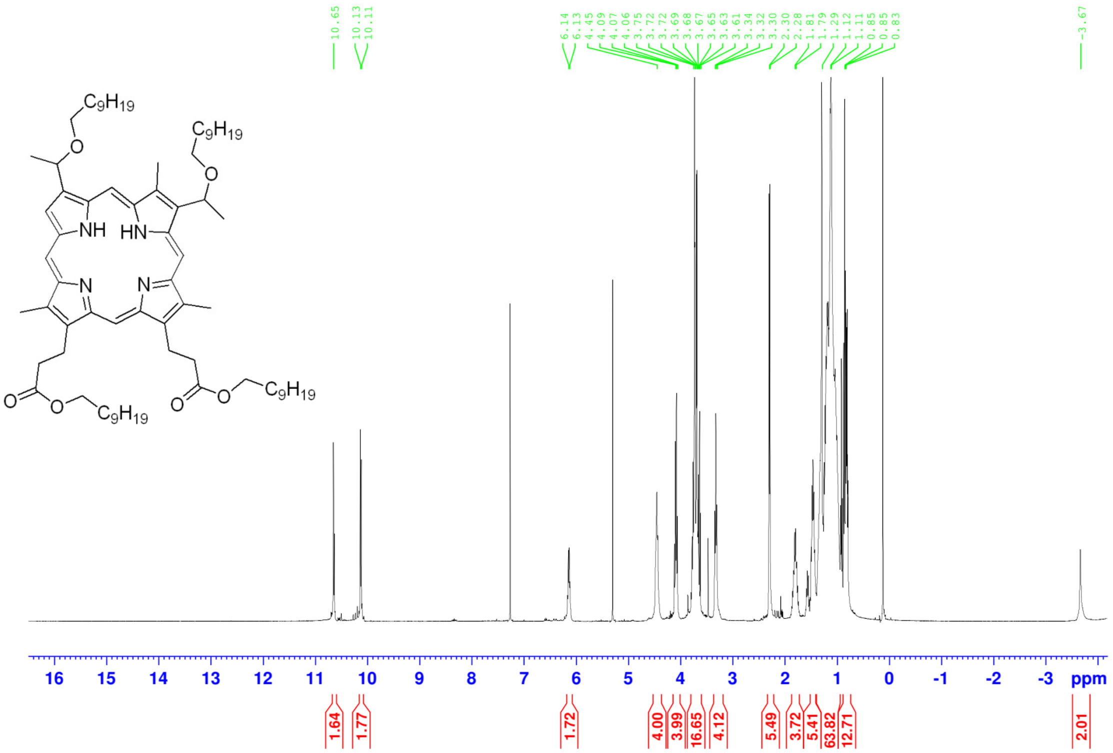

Figure S16: ^{1}**H NMR spectrum of compound 3d in CDCl**$_3$

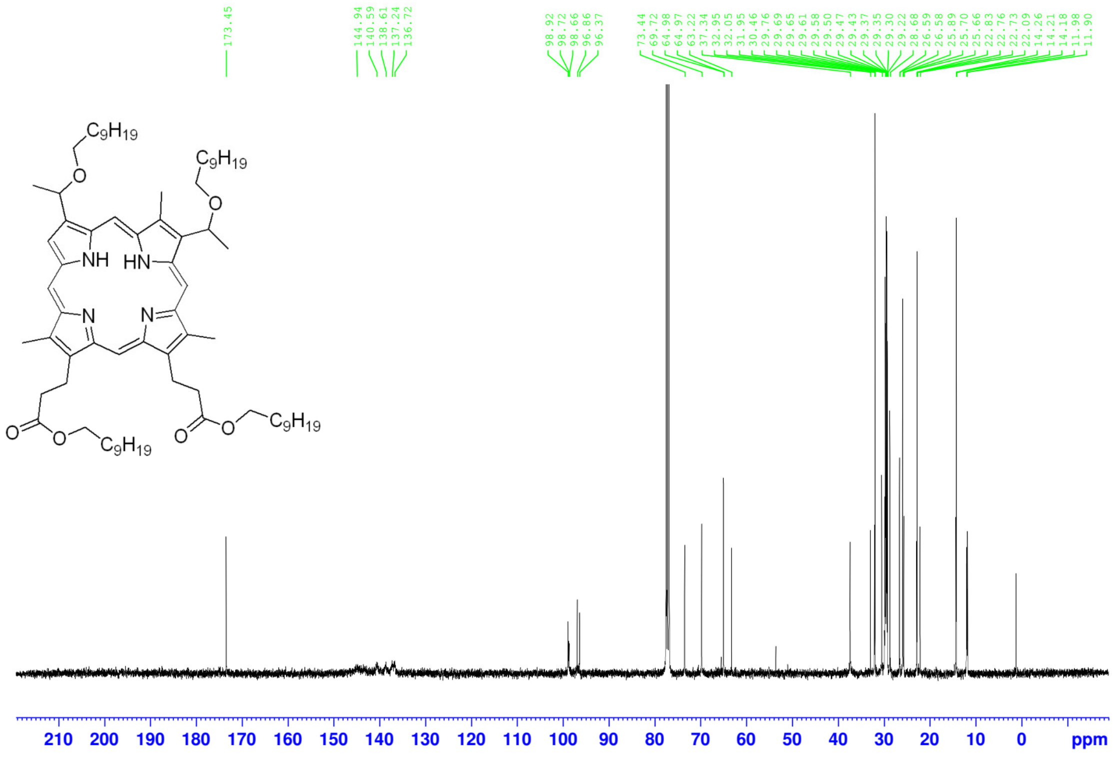

Figure S17: ^{13}**C NMR spectrum of compound 3d in CDCl**$_3$

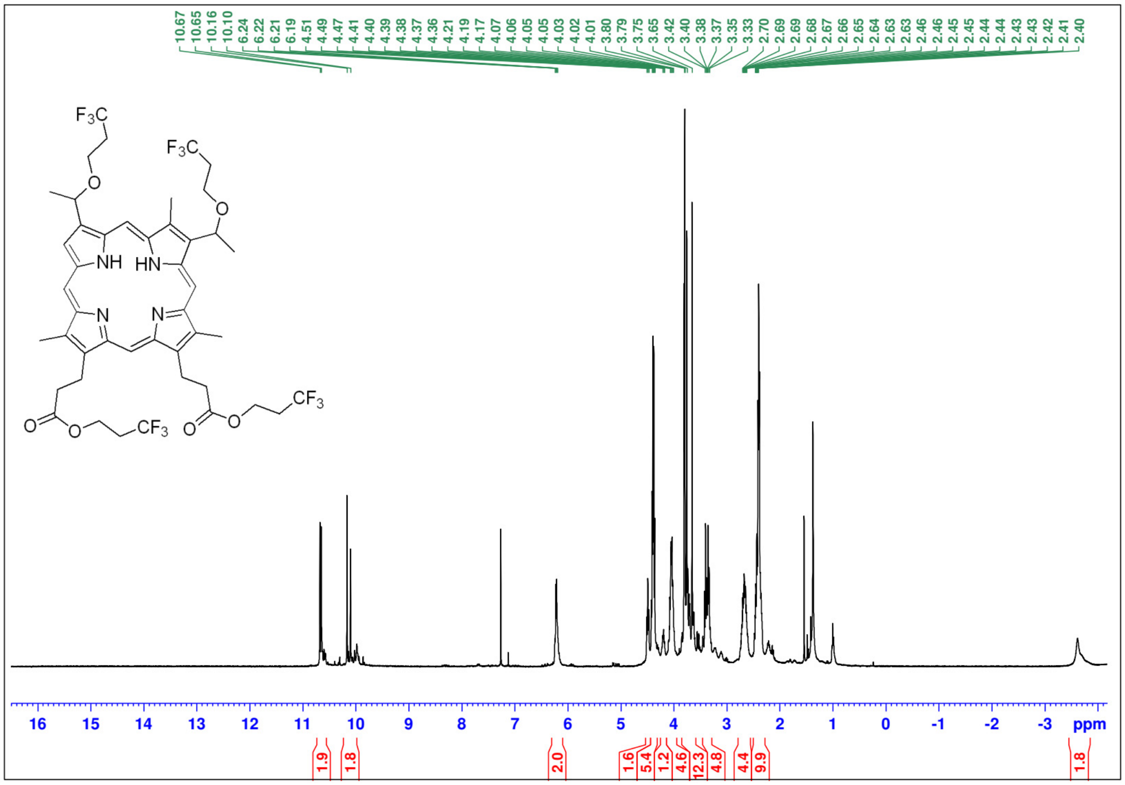

Figure S18: ^{1}**H NMR spectrum of compound 3e in CDCl**$_3$

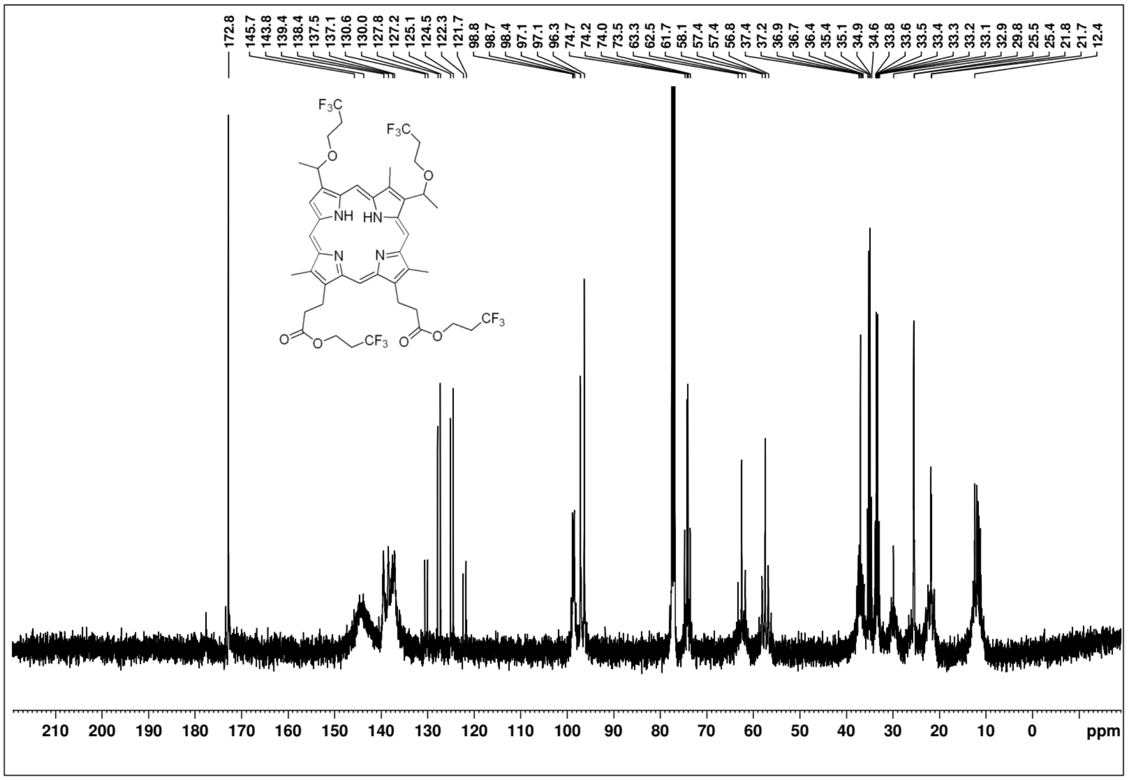

Figure S19: ^{13}**C NMR spectrum of compound 3e in CDCl**$_3$

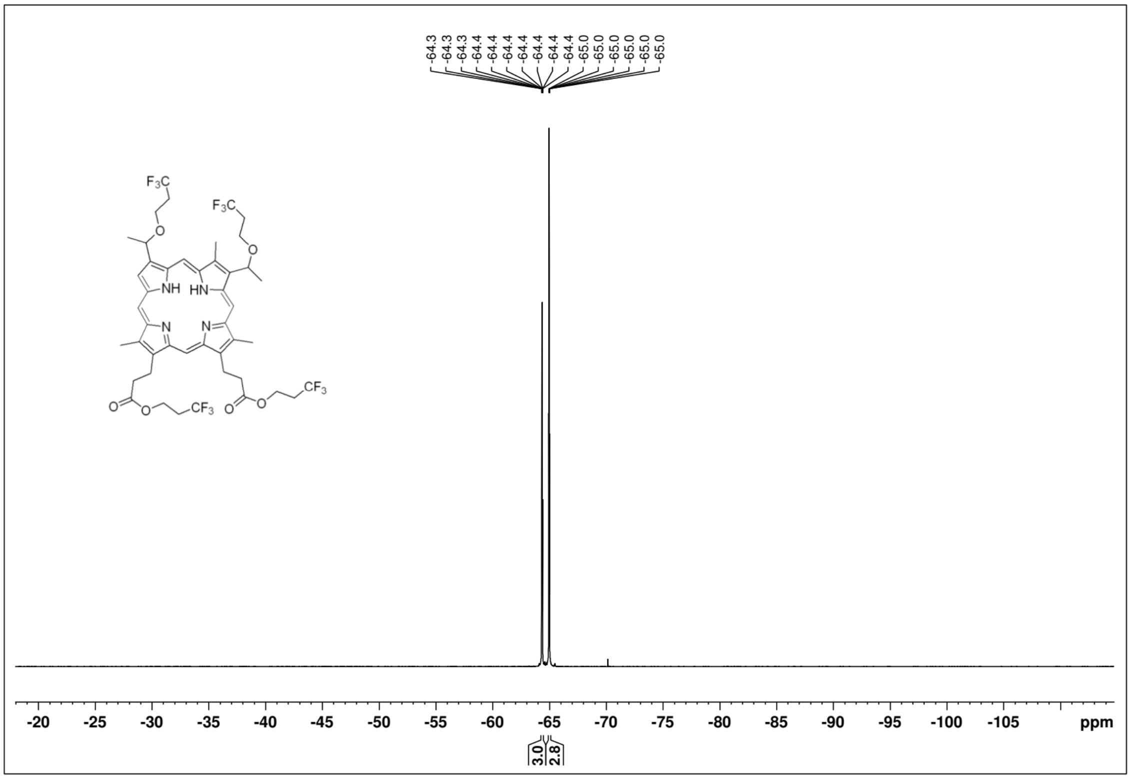

Figure S20: ^{19}F NMR spectrum of compound 3e in $CDCl_3$

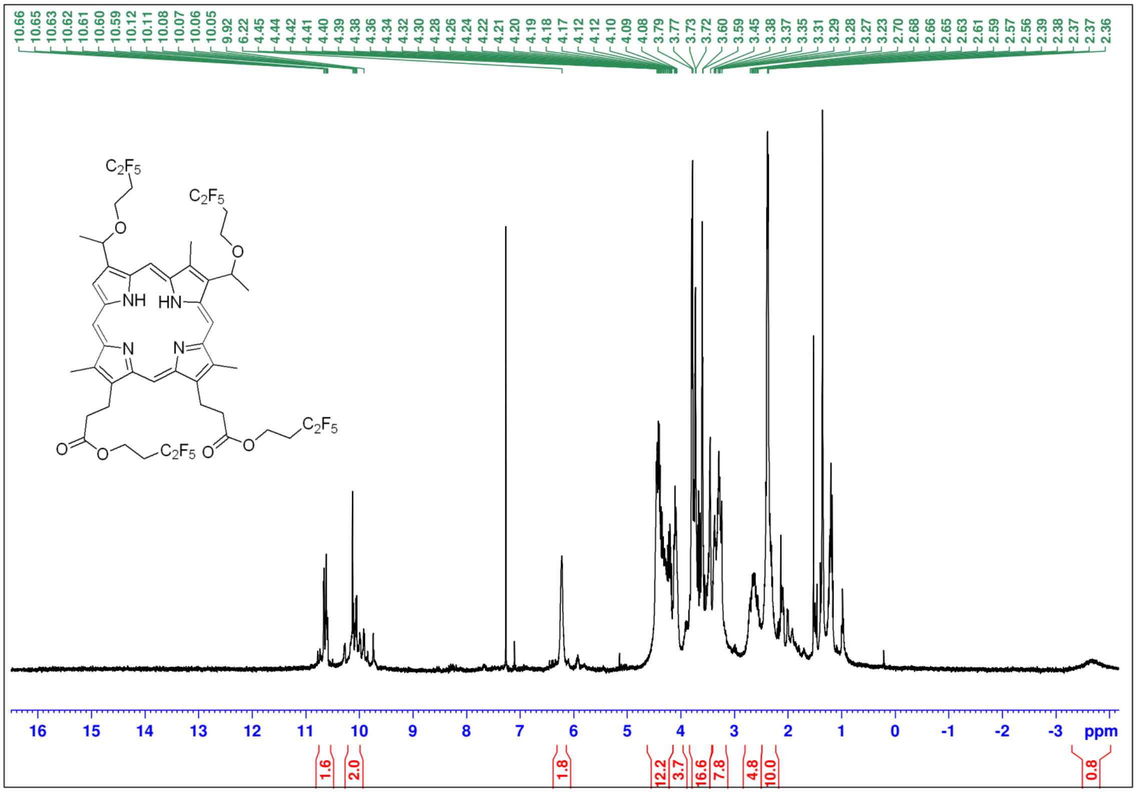

Figure S21: ^{1}H NMR spectrum of compound 3f in $CDCl_3$

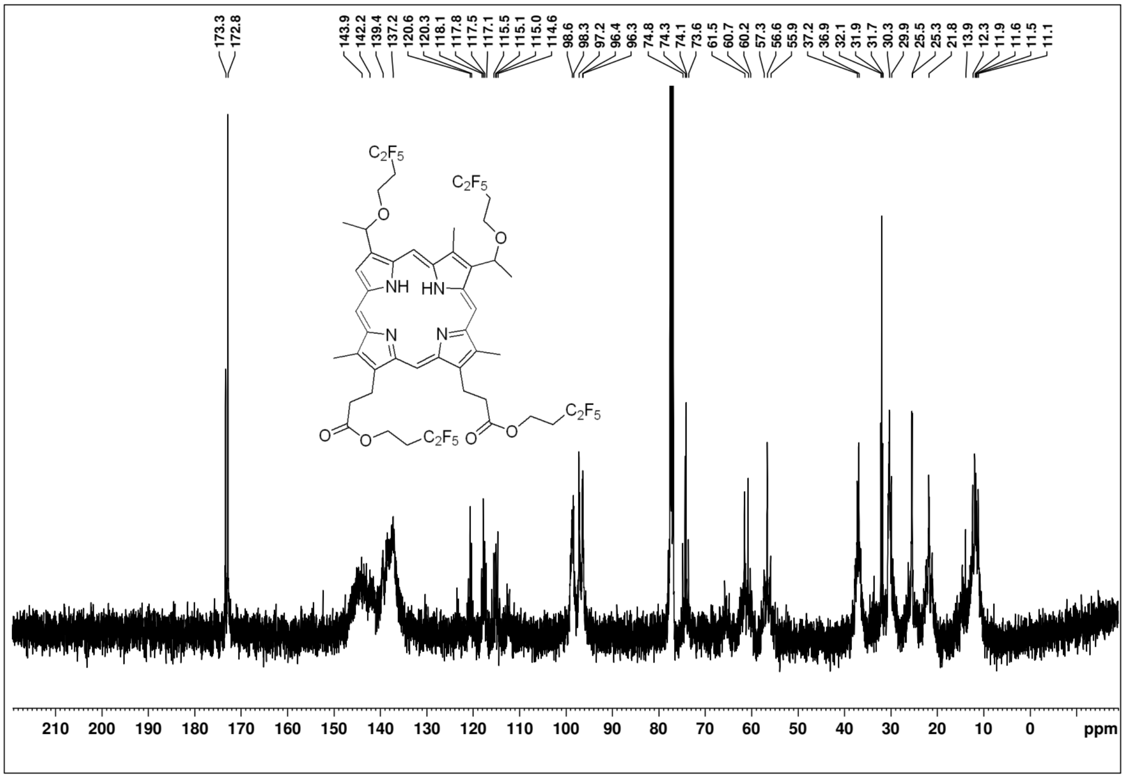

Figure S22: ^{13}C NMR spectrum of compound 3f in CDCl3

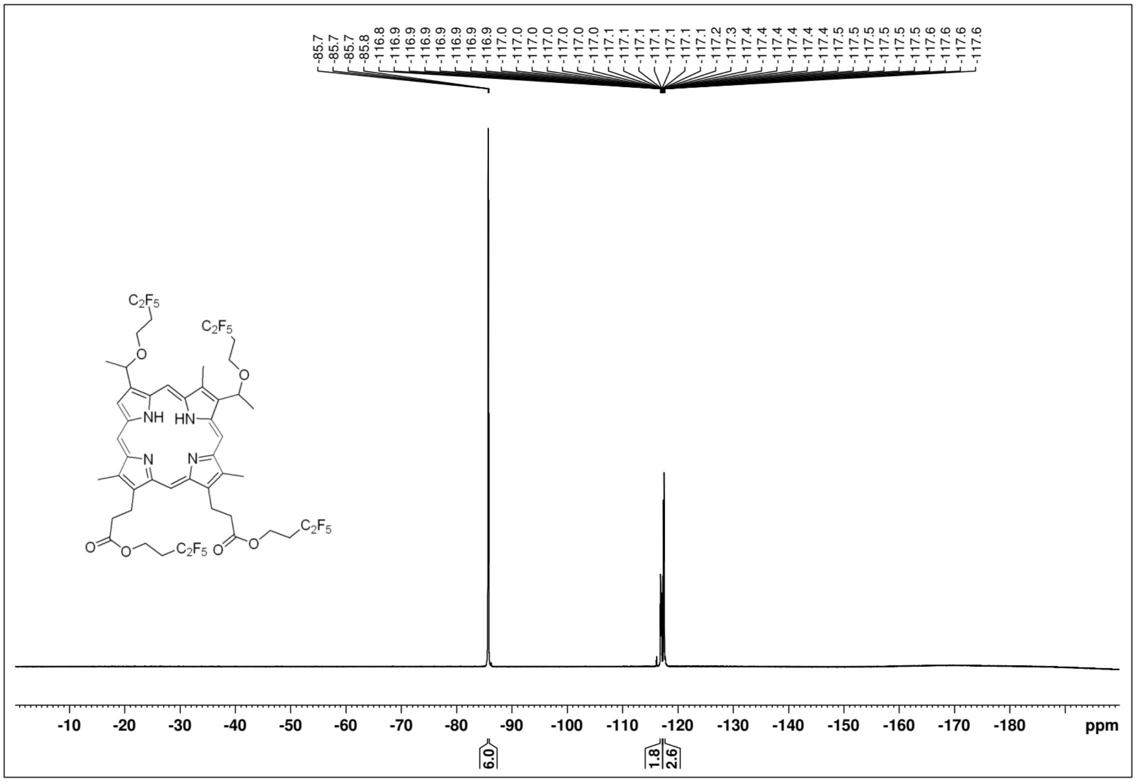

Figure S23: ^{19}F NMR spectrum of compound 3f in CDCl3

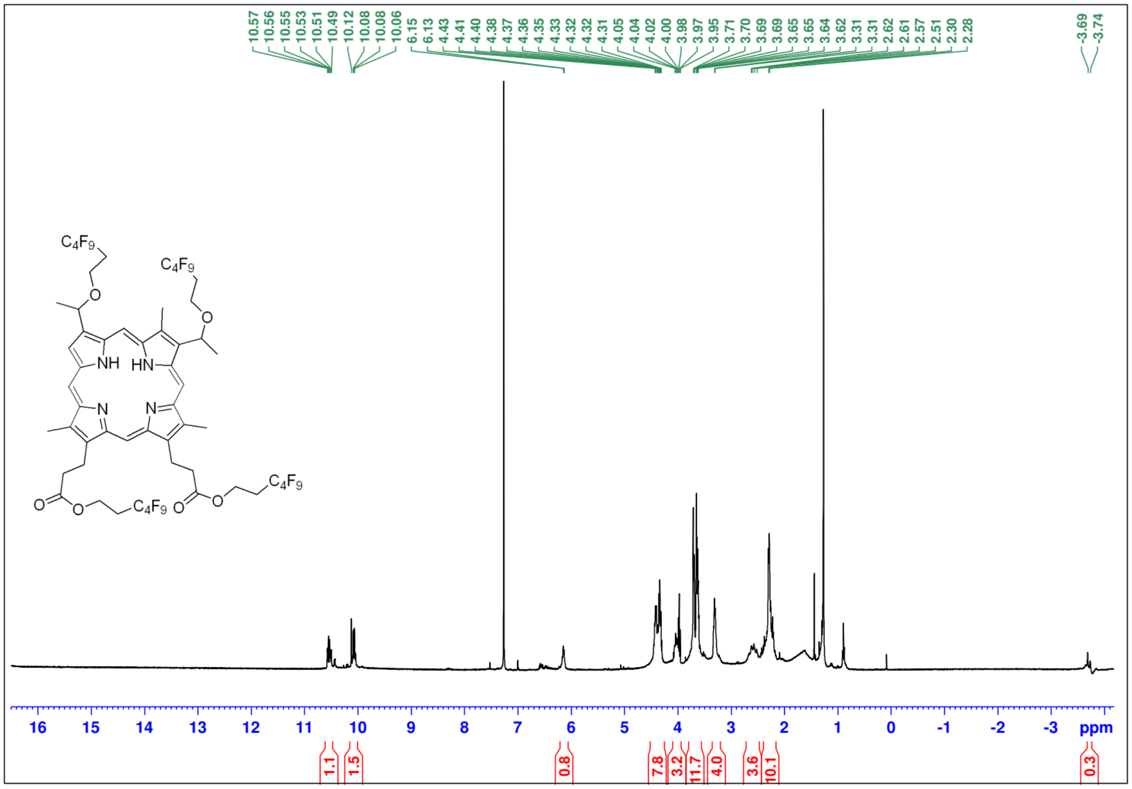

Figure S24: ^{1}H NMR spectrum of compound 3g in $CDCl_3$

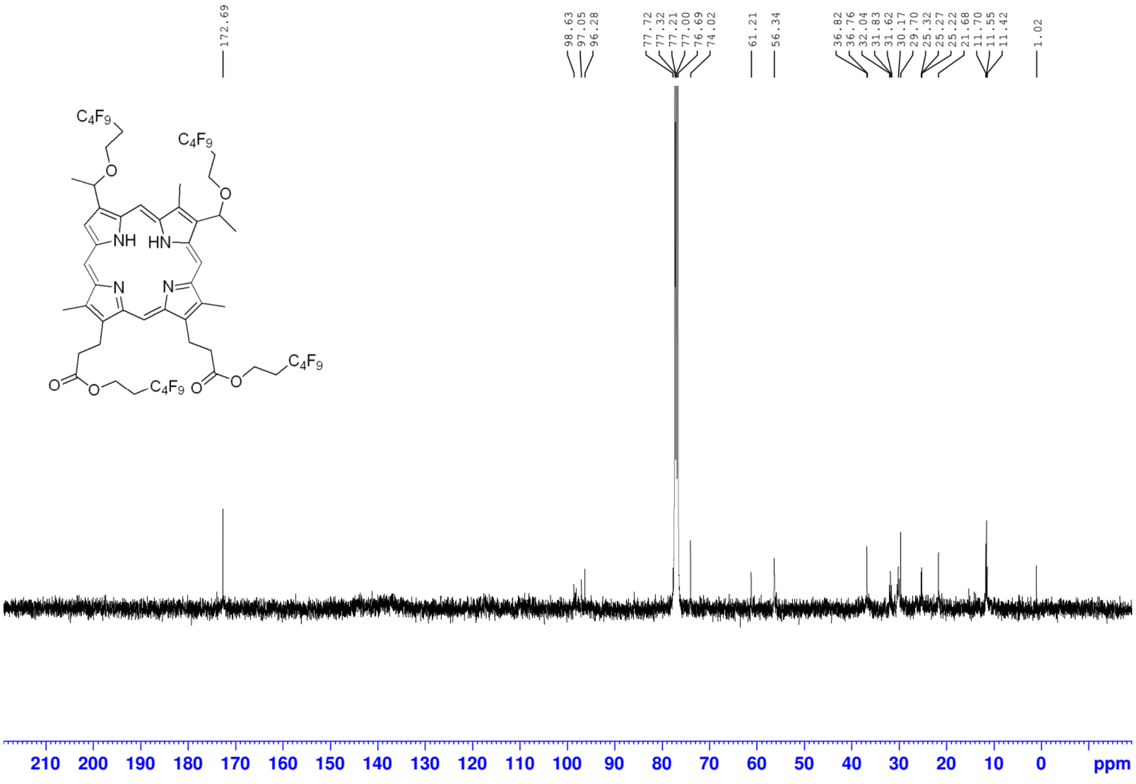

Figure S25: ^{13}C NMR spectrum of compound 3g in $CDCl_3$

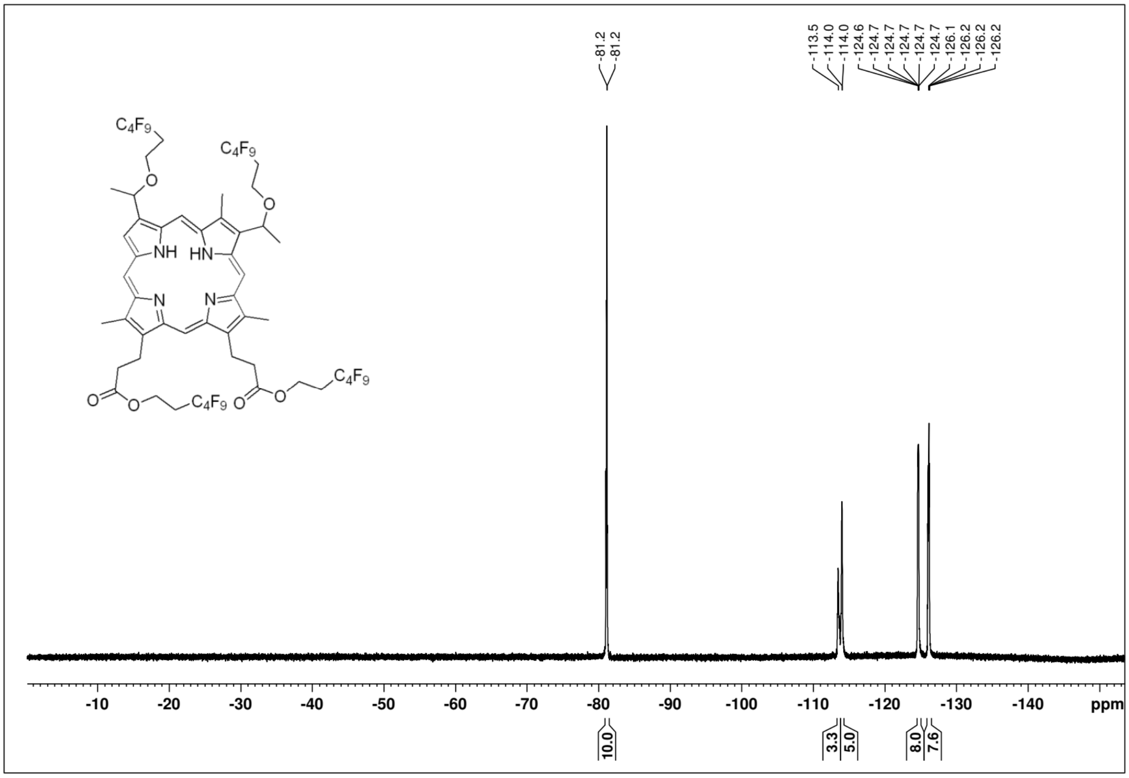

Figure S26: ^{19}F **NMR spectrum of compound 3g in** $CDCl_3$

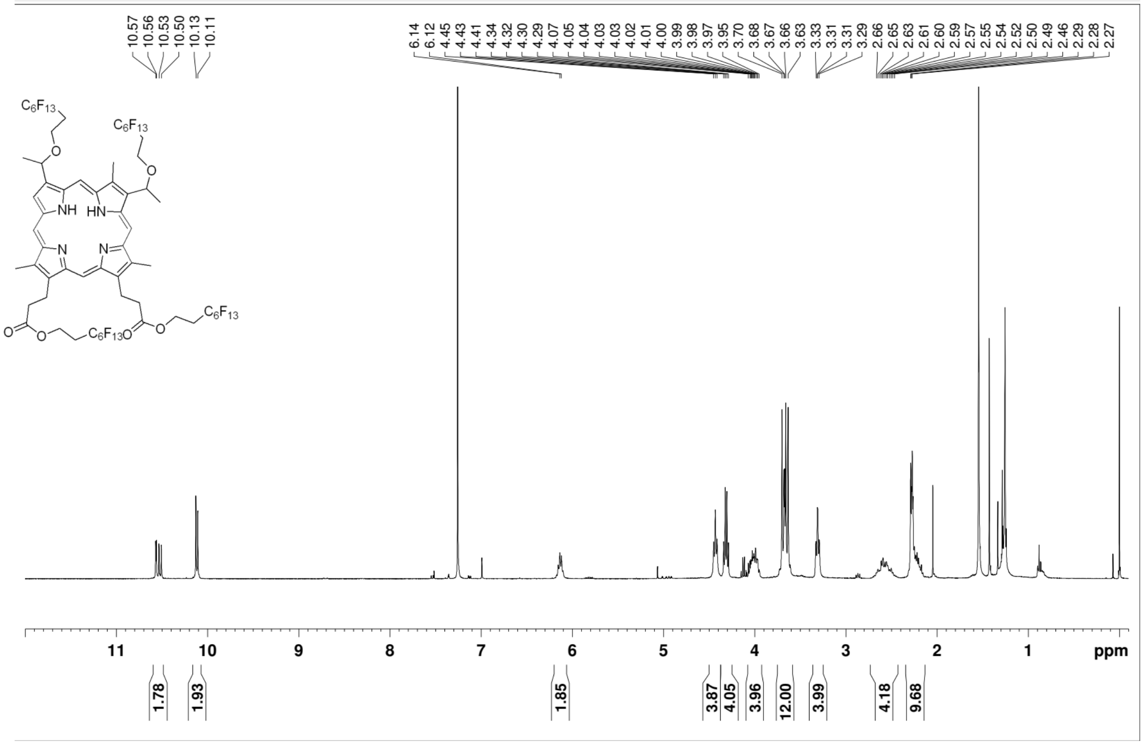

Figure S27: ^{1}H **NMR spectrum of compound 3h in** $CDCl_3$

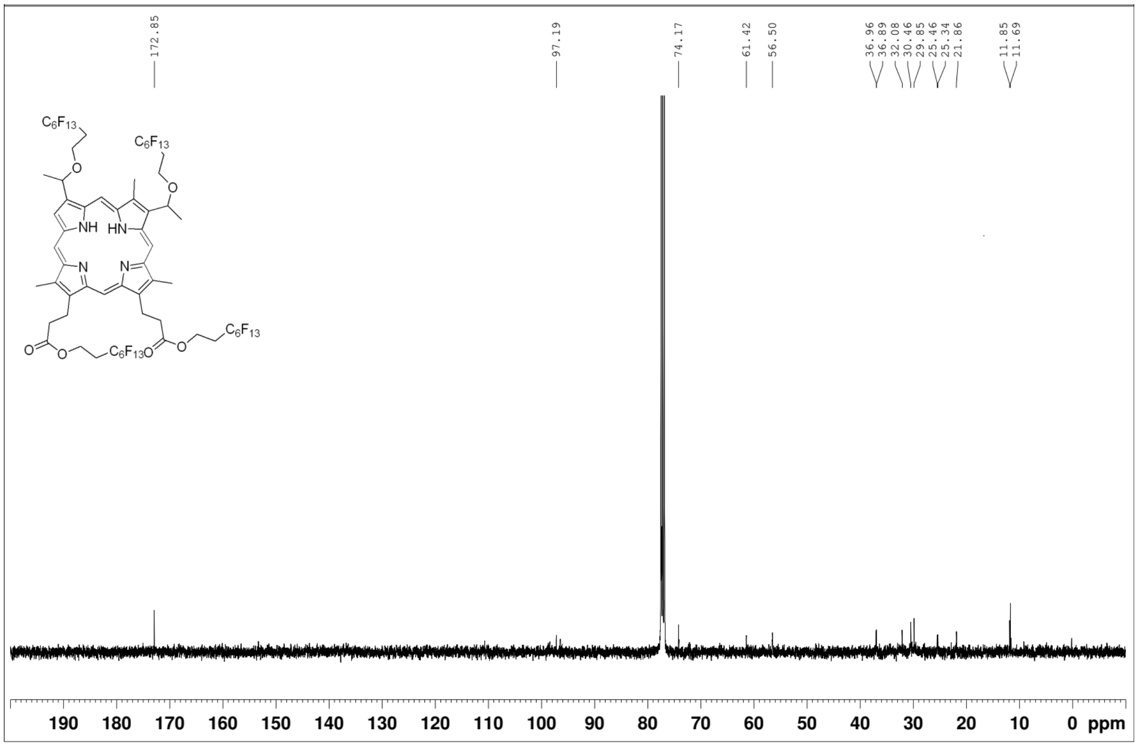

Figure S28: ^{13}C NMR spectrum of compound 3h in $CDCl_3$

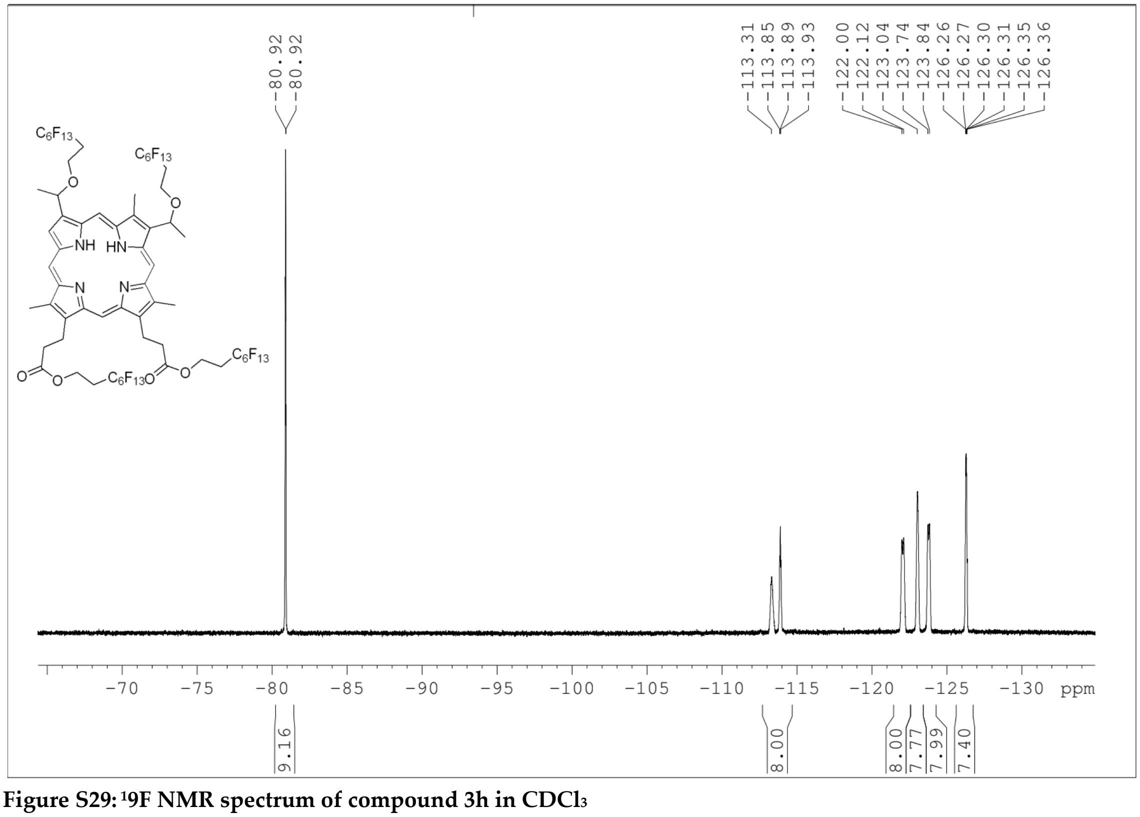

Figure S29: ^{19}F NMR spectrum of compound 3h in $CDCl_3$

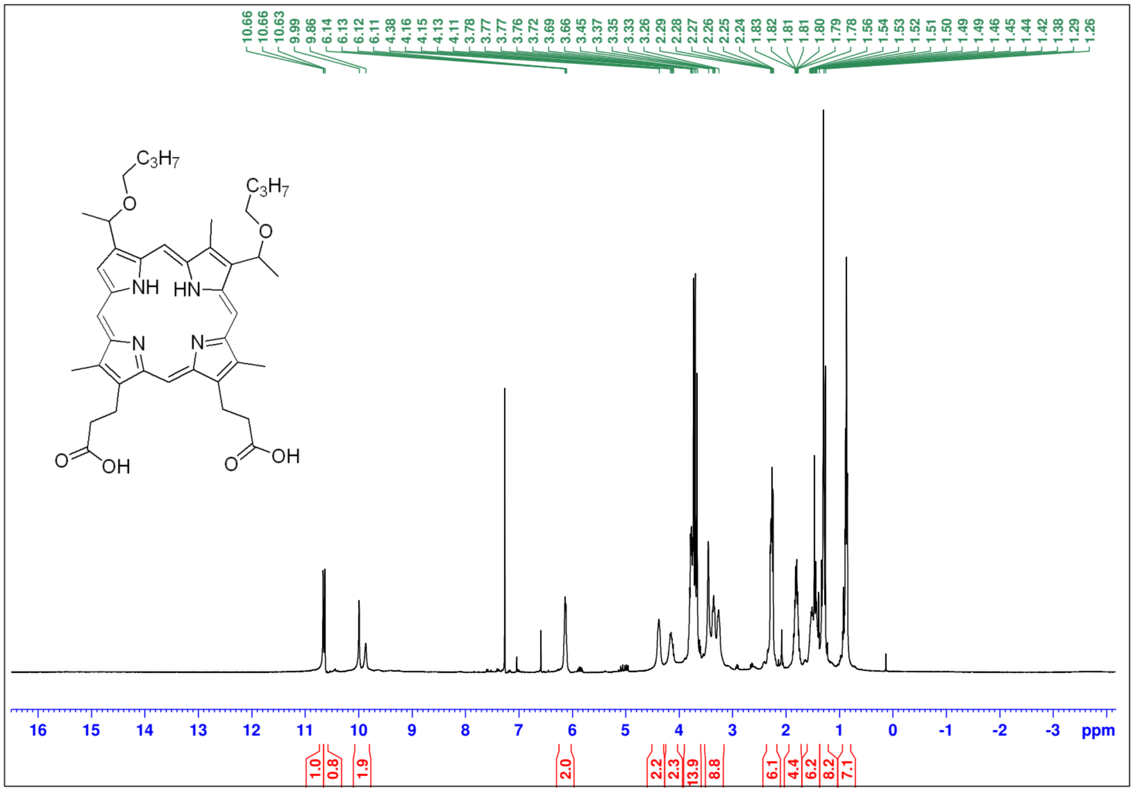

Figure S30: ^{1}H **NMR spectrum of compound 4a in** $CDCl_3$

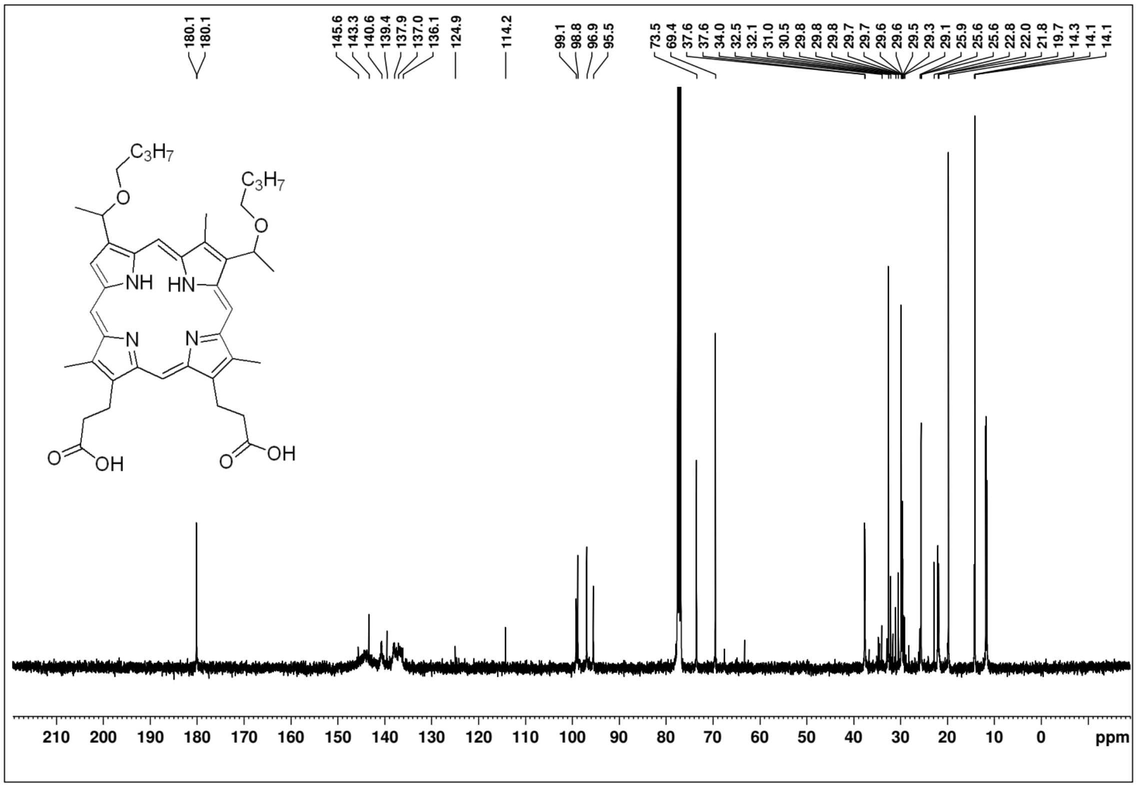

Figure S31: ^{13}C **NMR spectrum of compound 4a in** $CDCl_3$

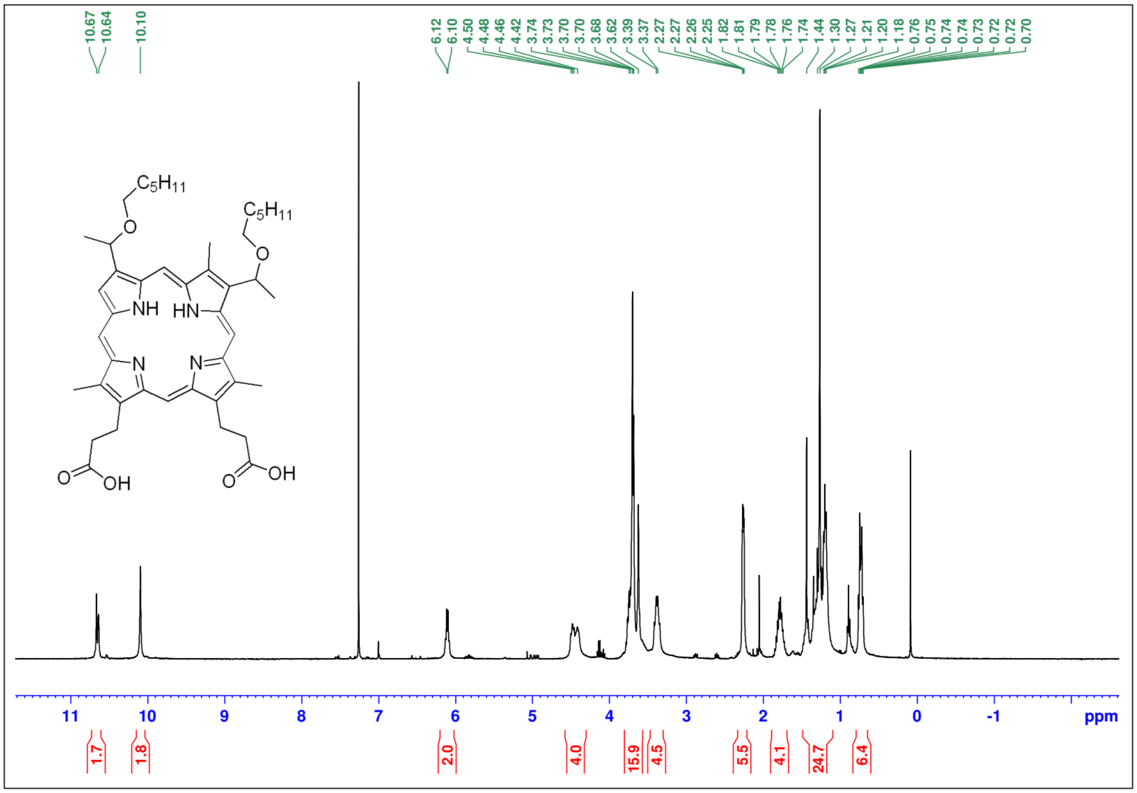

Figure S32: ^{1}H **NMR spectrum of compound 4b in** $CDCl_3$

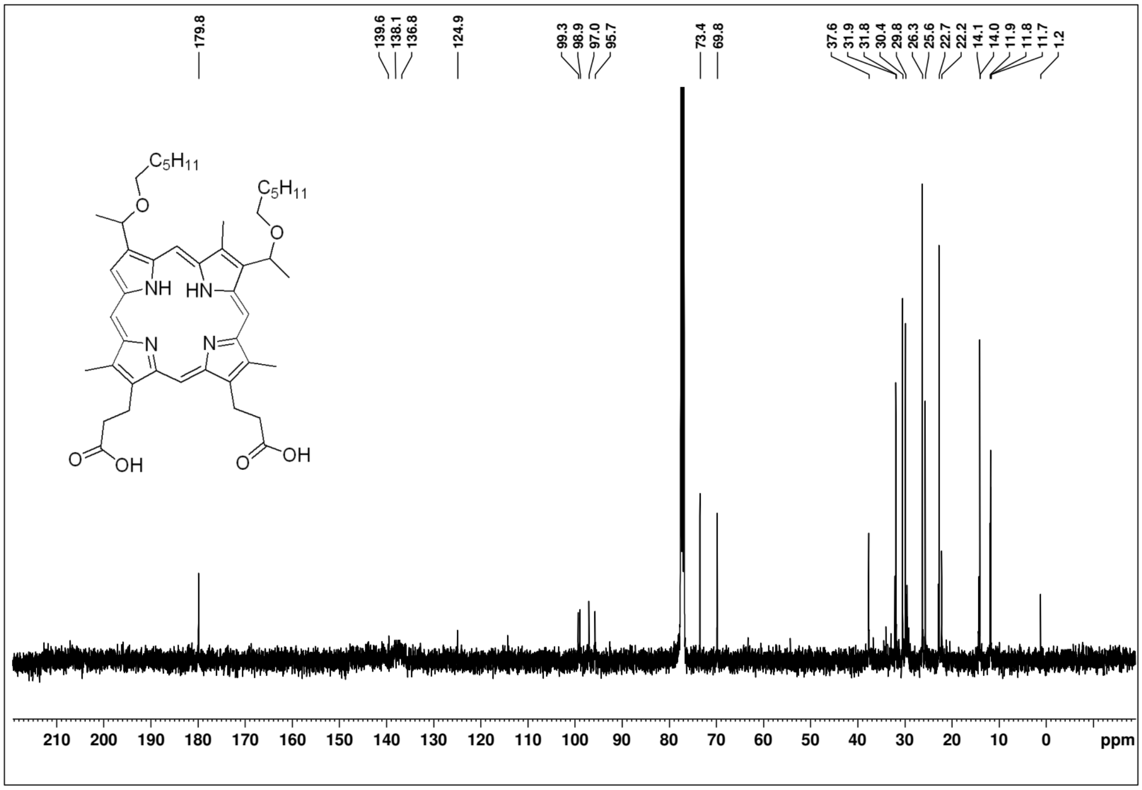

Figure S33: ^{13}C **NMR spectrum of compound 4b in** $CDCl_3$

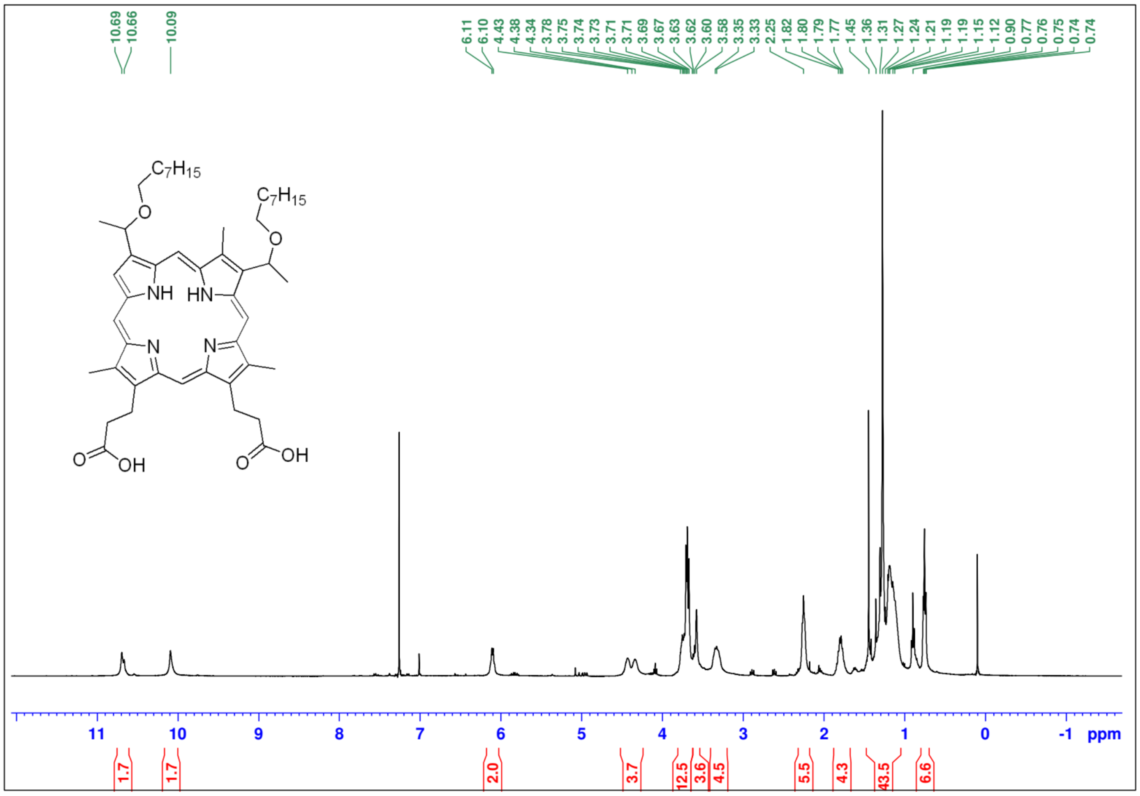

Figure S34: ^1H **NMR spectrum of compound 4c in** $CDCl_3$

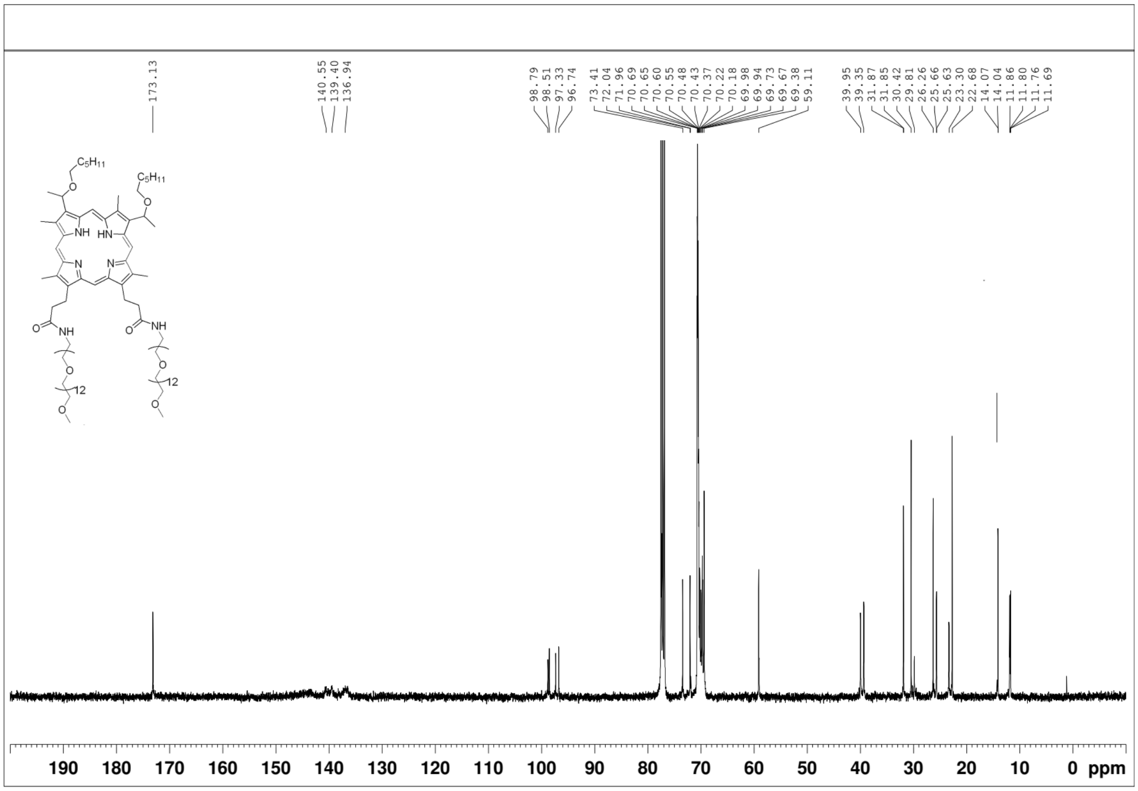

Figure S35: ^{13}C **NMR spectrum of compound 4c in** $CDCl_3$

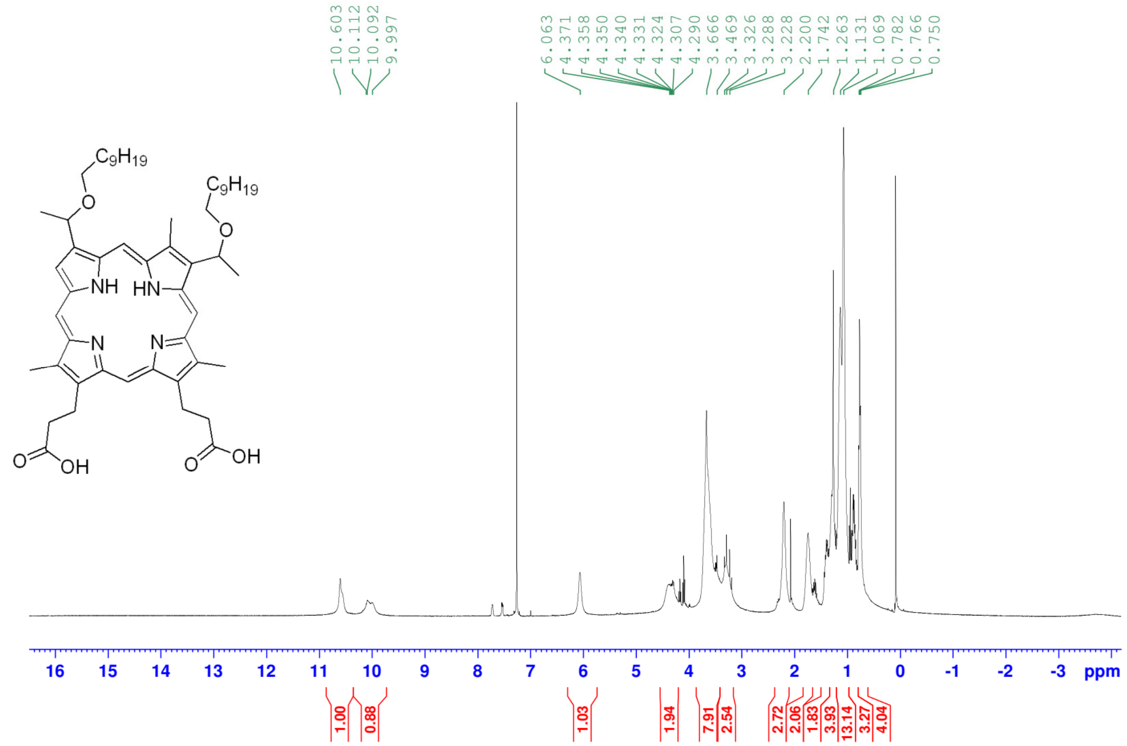

Figure S36: ^{1}H NMR spectrum of compound 4d in $CDCl_3$

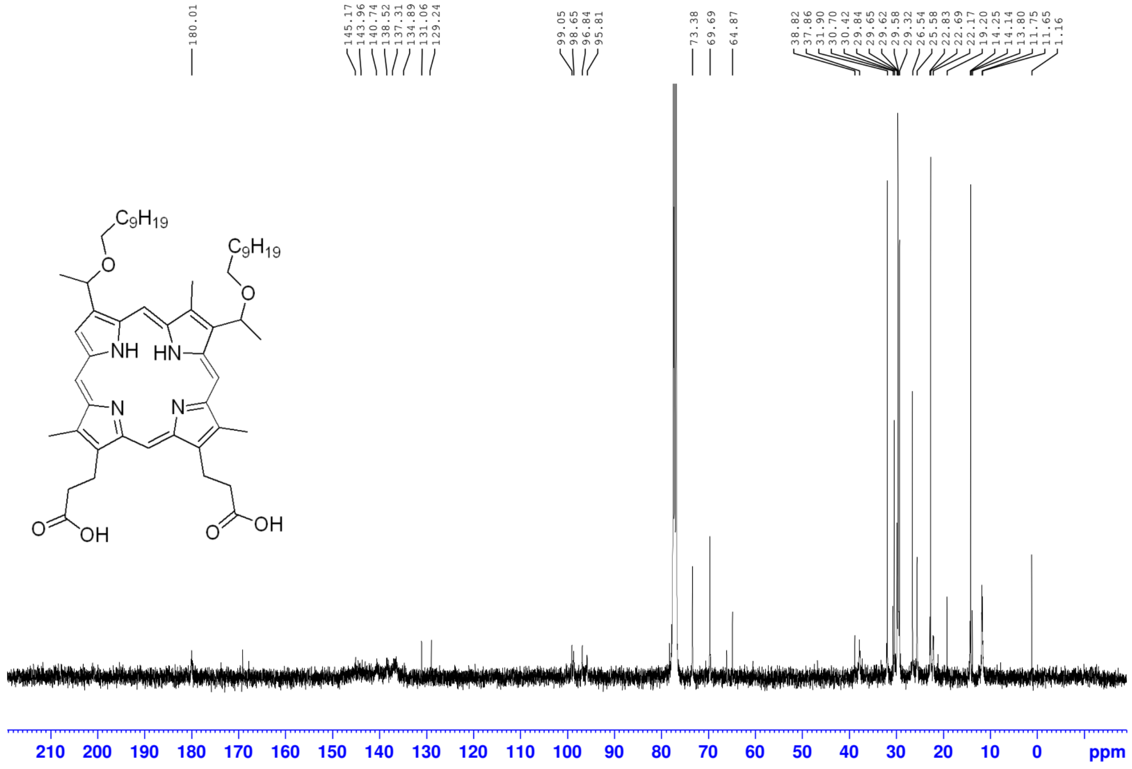

Figure S37: ^{13}C NMR spectrum of compound 4d in $CDCl_3$

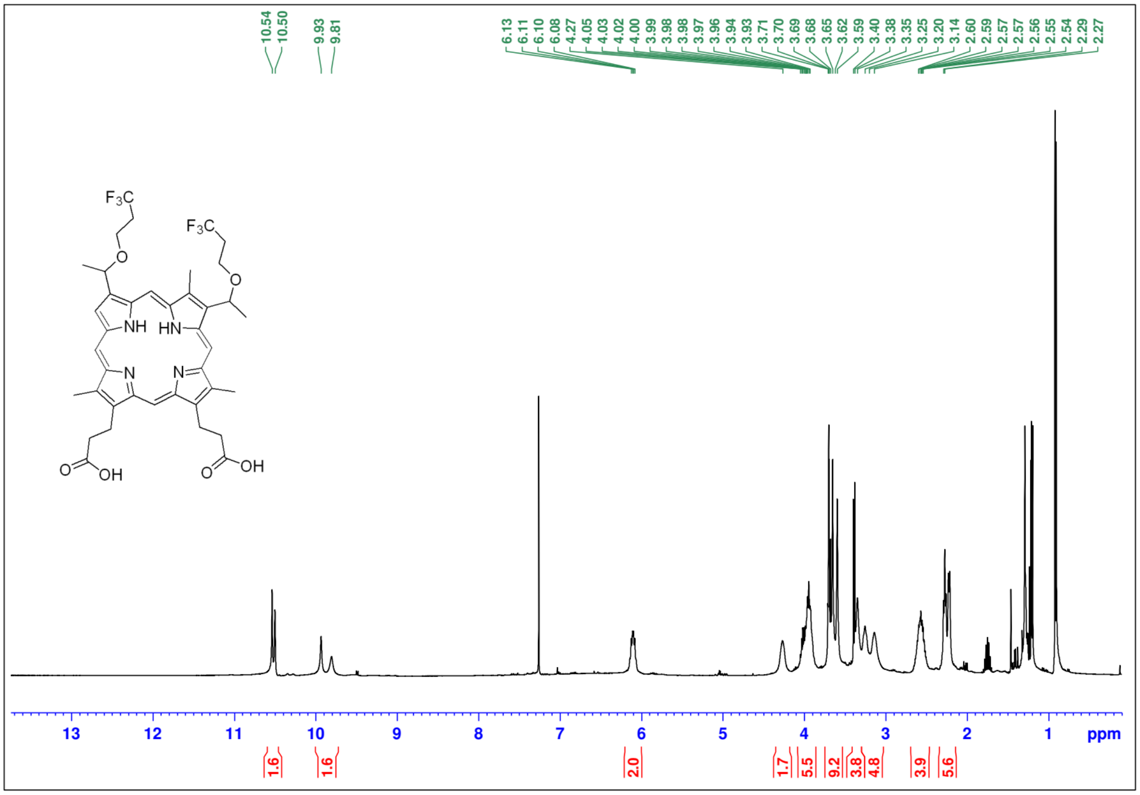

Figure S38: ¹H NMR spectrum of compound 4e in $CDCl_3$

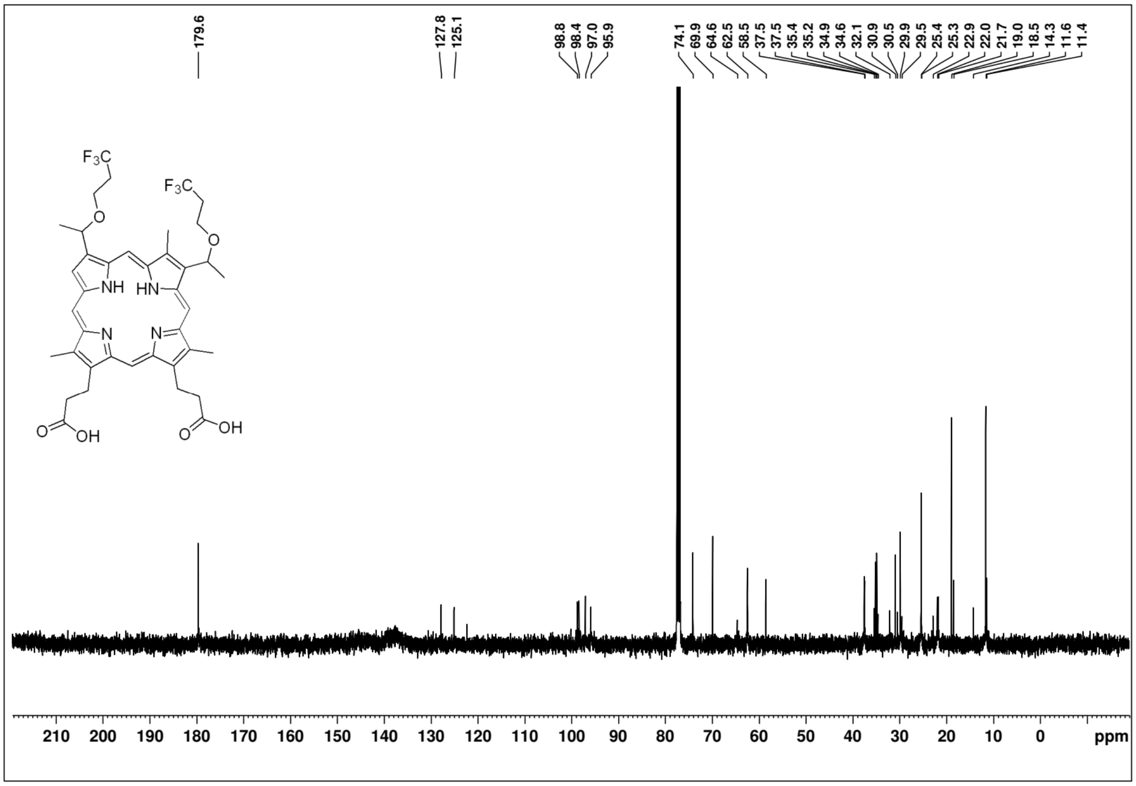

Figure S39: ¹³C NMR spectrum of compound 4e in $CDCl_3$

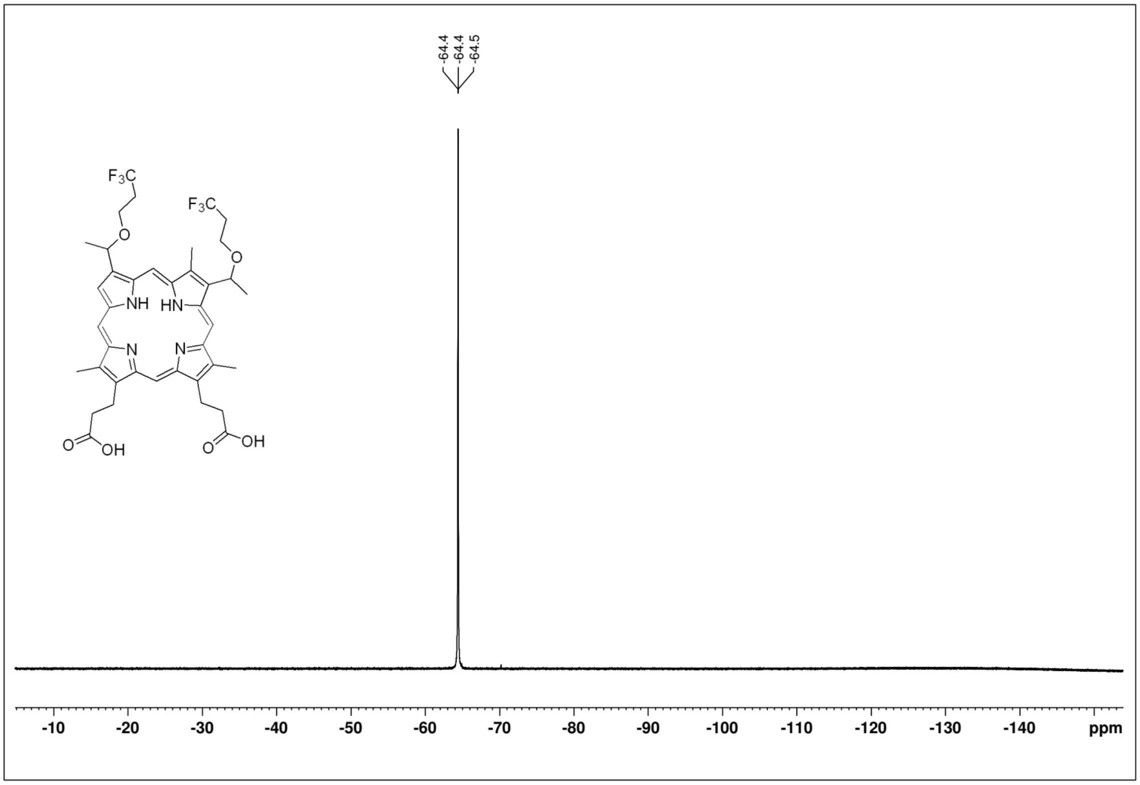

Figure S40: ^{19}F NMR spectrum of compound 4e in $CDCl_3$

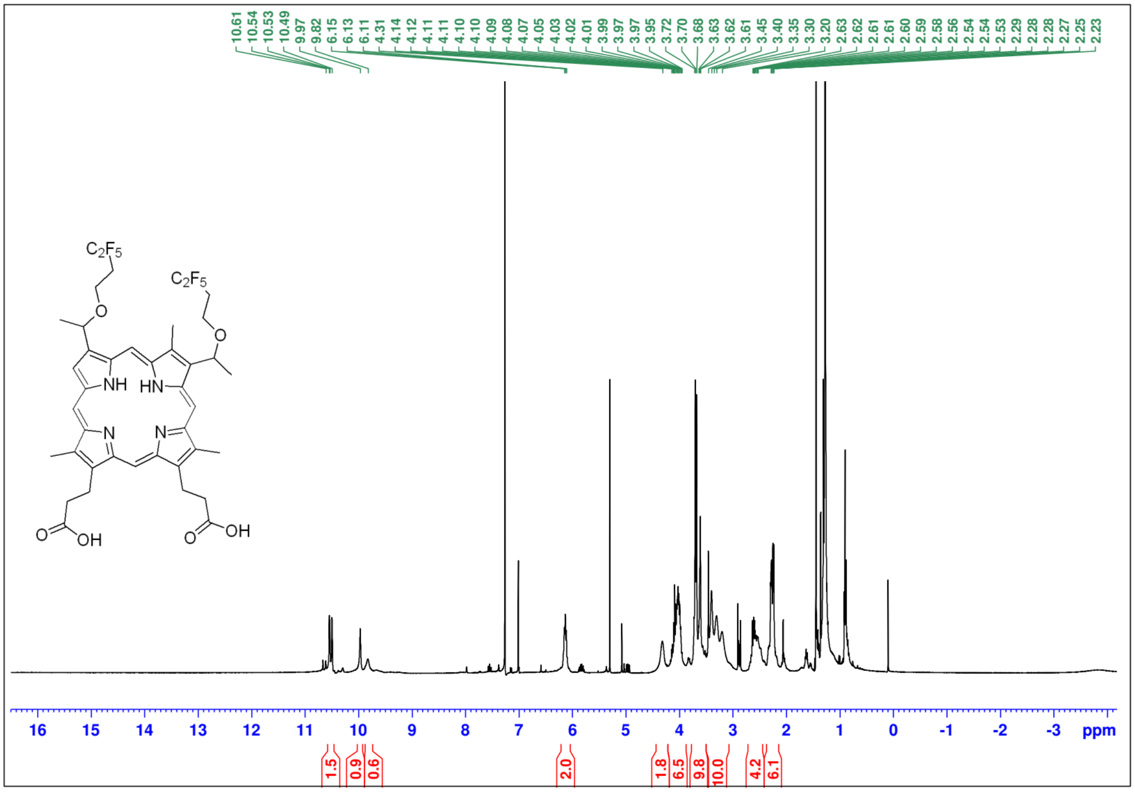

Figure S41: ^{1}H NMR spectrum of compound 4f in $CDCl_3$

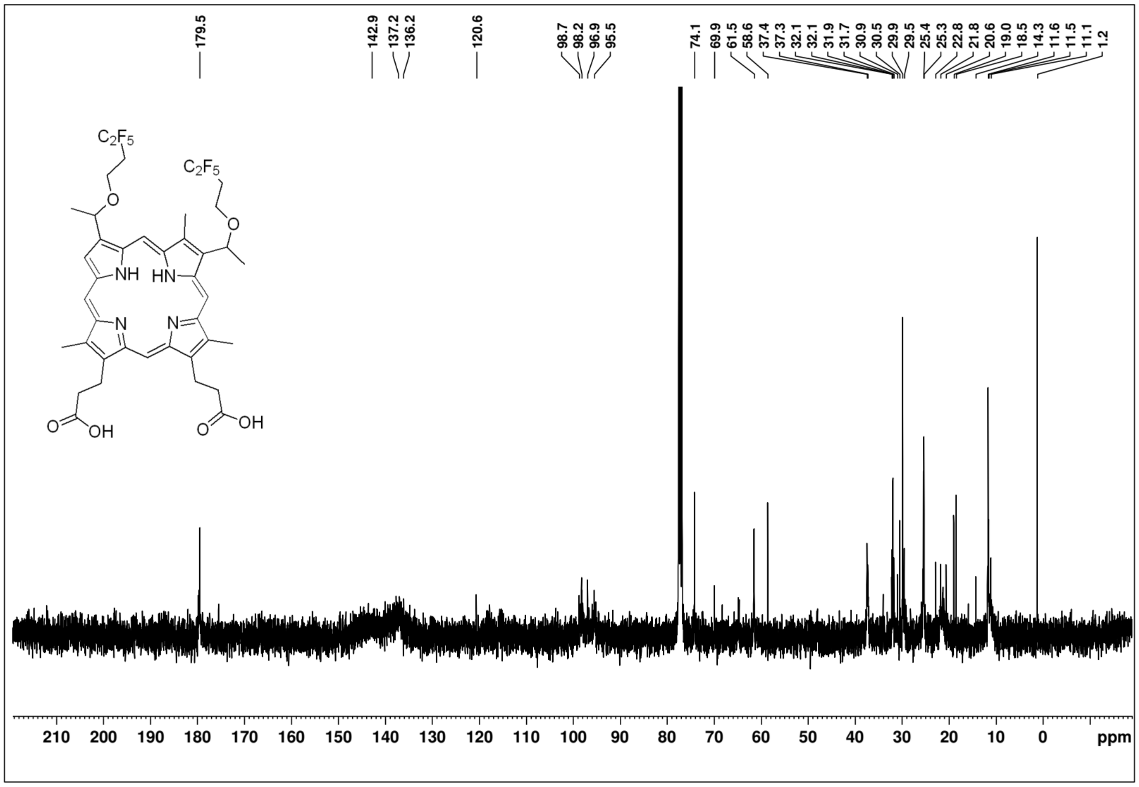

Figure S42: ^{13}C **NMR spectrum of compound 4f in** $CDCl_3$

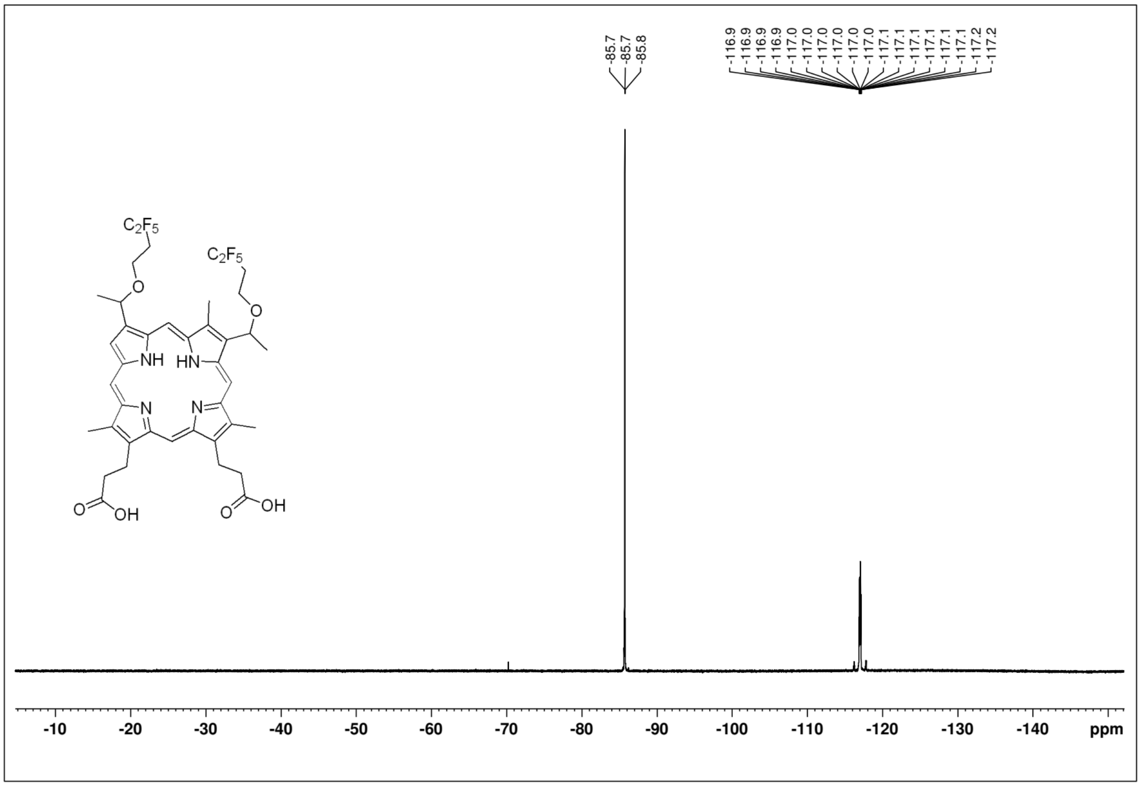

Figure S43 : ^{19}F **NMR spectrum of compound 4f in** $CDCl_3$

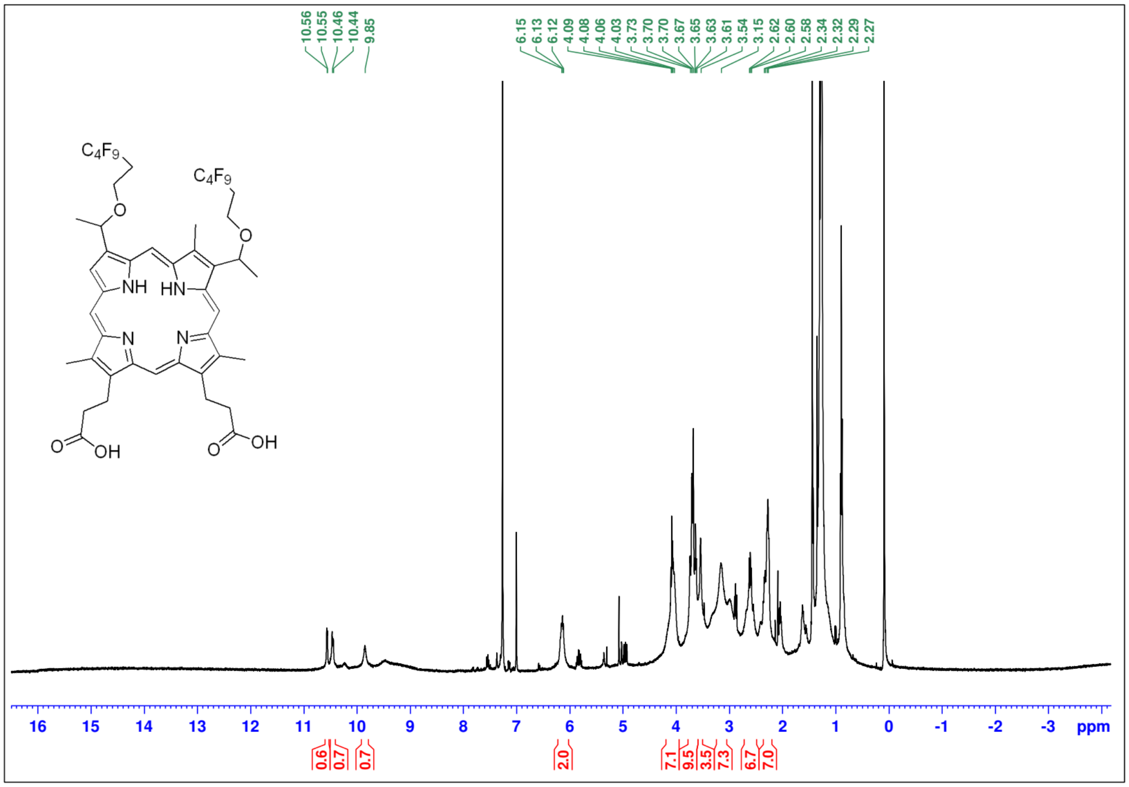

Figure S44: ¹H NMR spectrum of compound 4g in CDCl₃

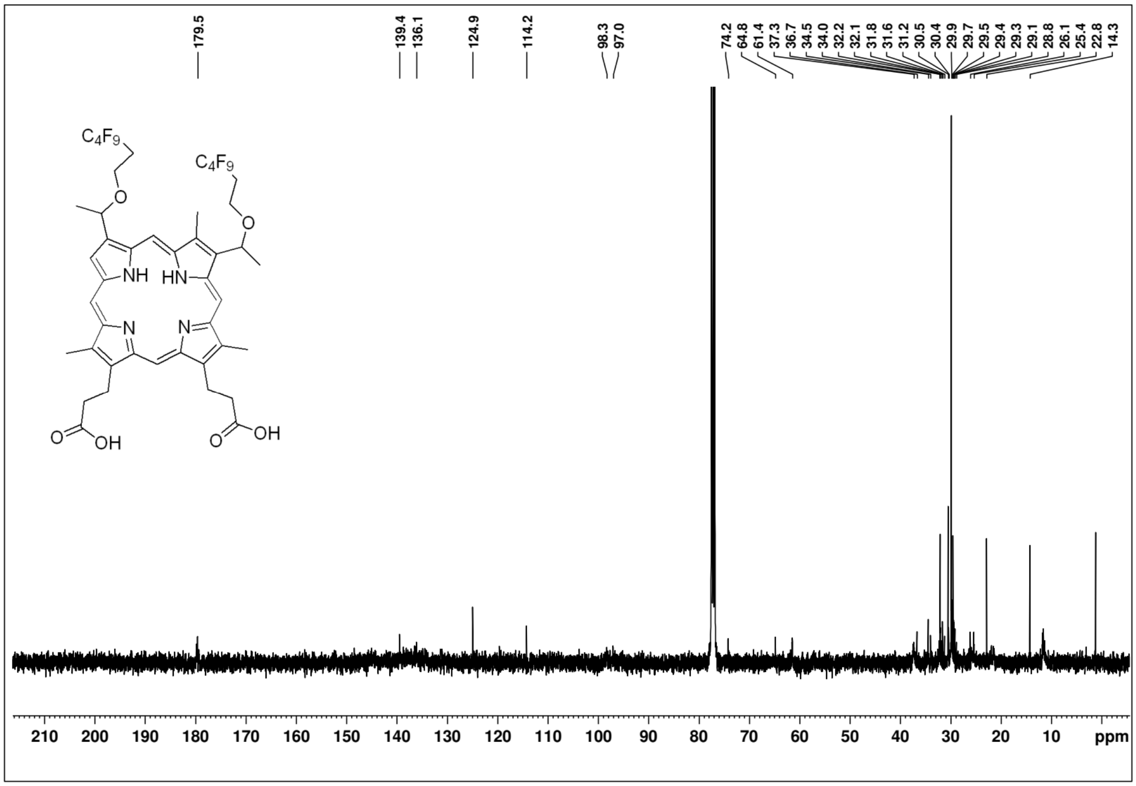

Figure S45: ¹³C NMR spectrum of compound 4ga in CDCl₃

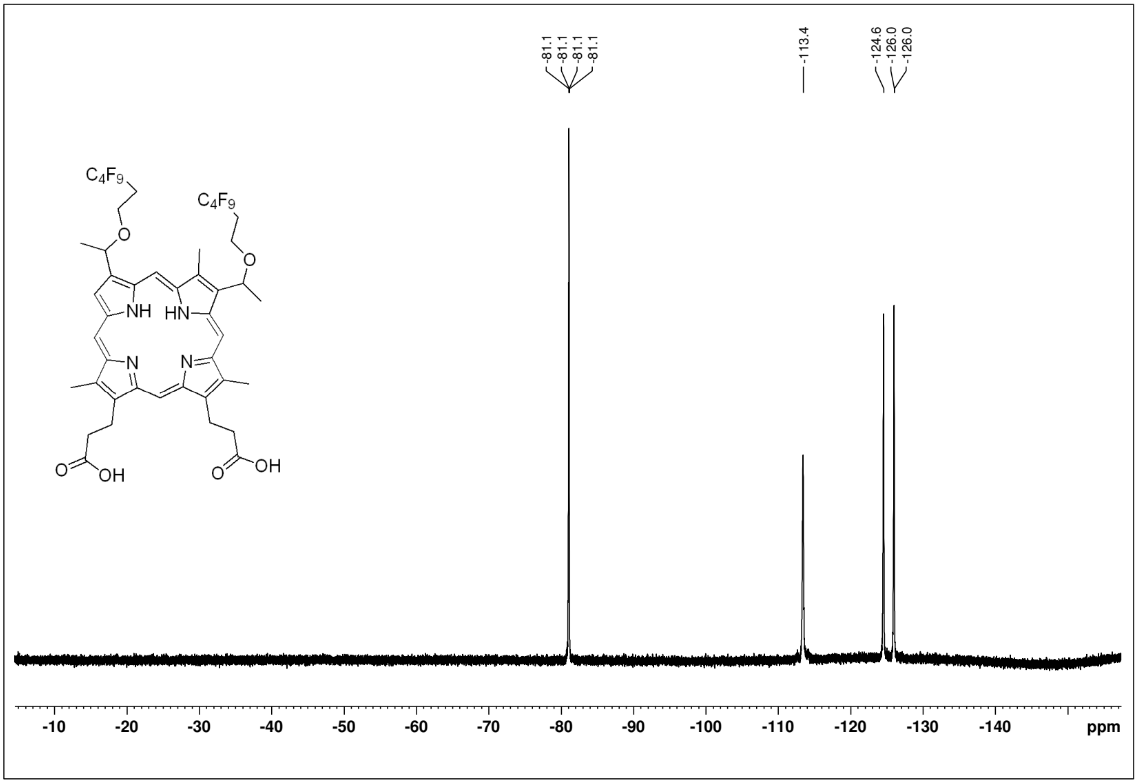

Figure S46: ^{13}C **NMR spectrum of compound 4g in** $CDCl_3$

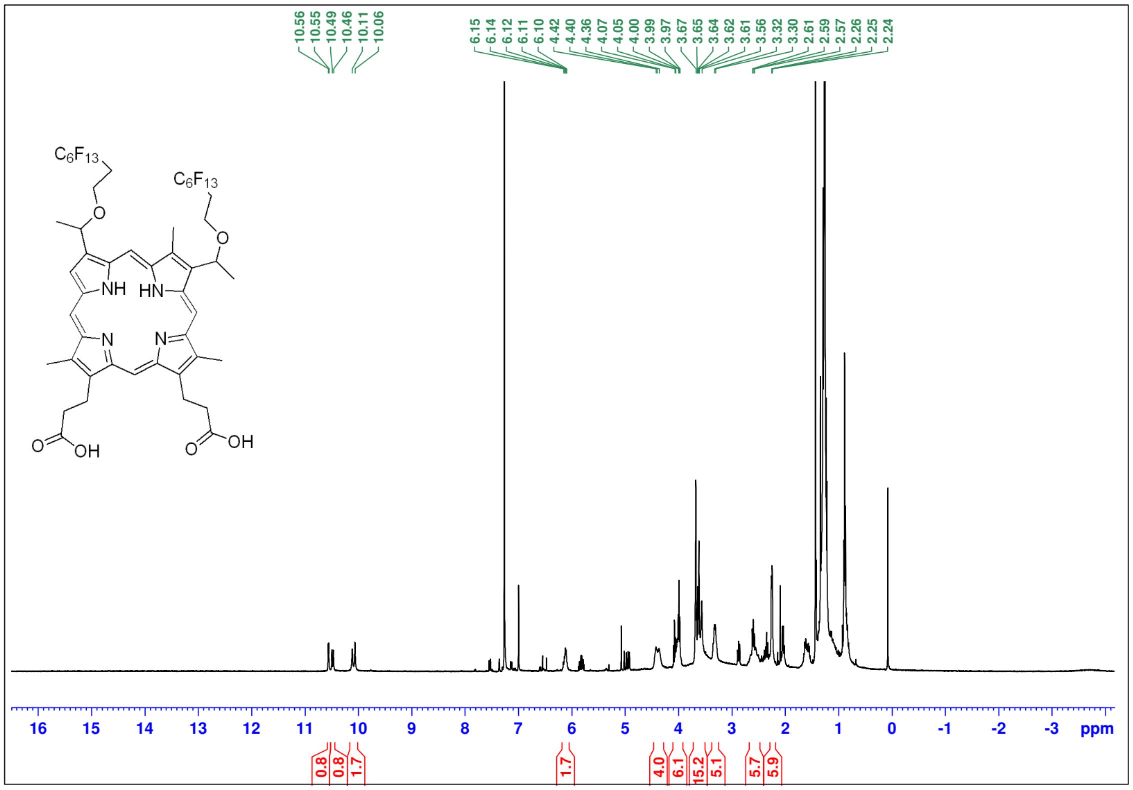

Figure S47: ^{1}H **NMR spectrum of compound 4h in** $CDCl_3$

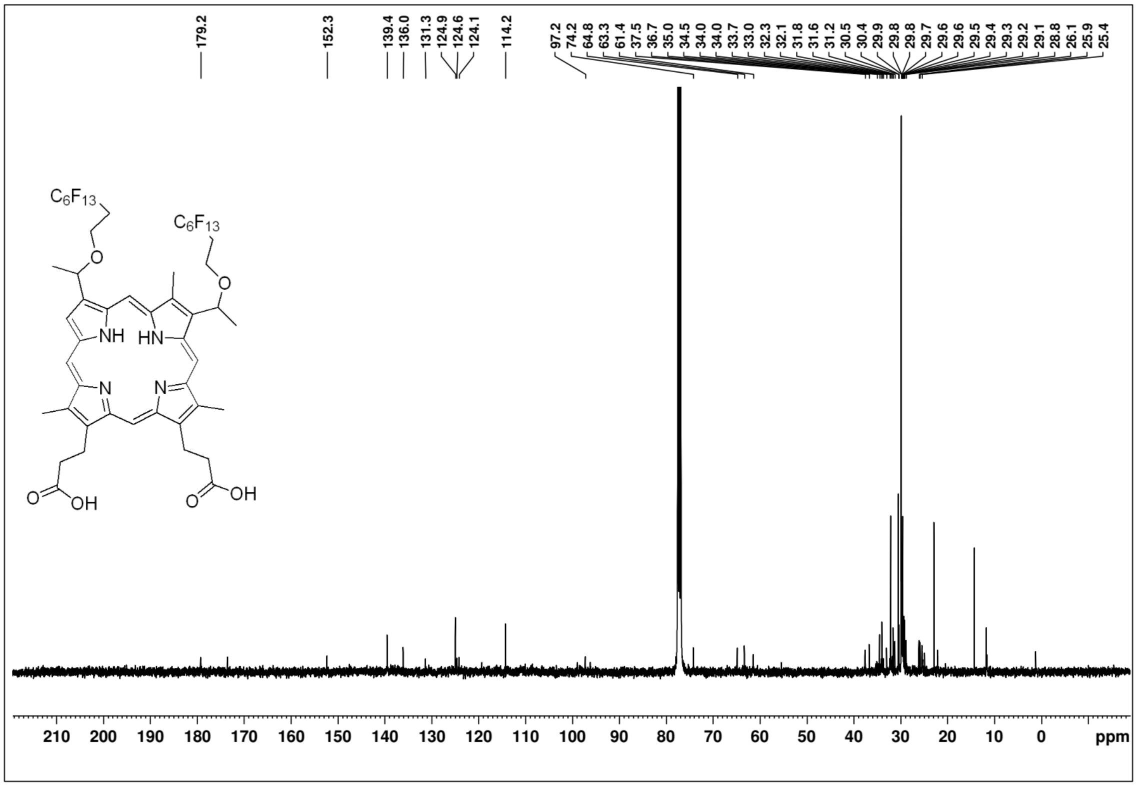

Figure S48: ^{13}C NMR spectrum of compound 4h in $CDCl_3$

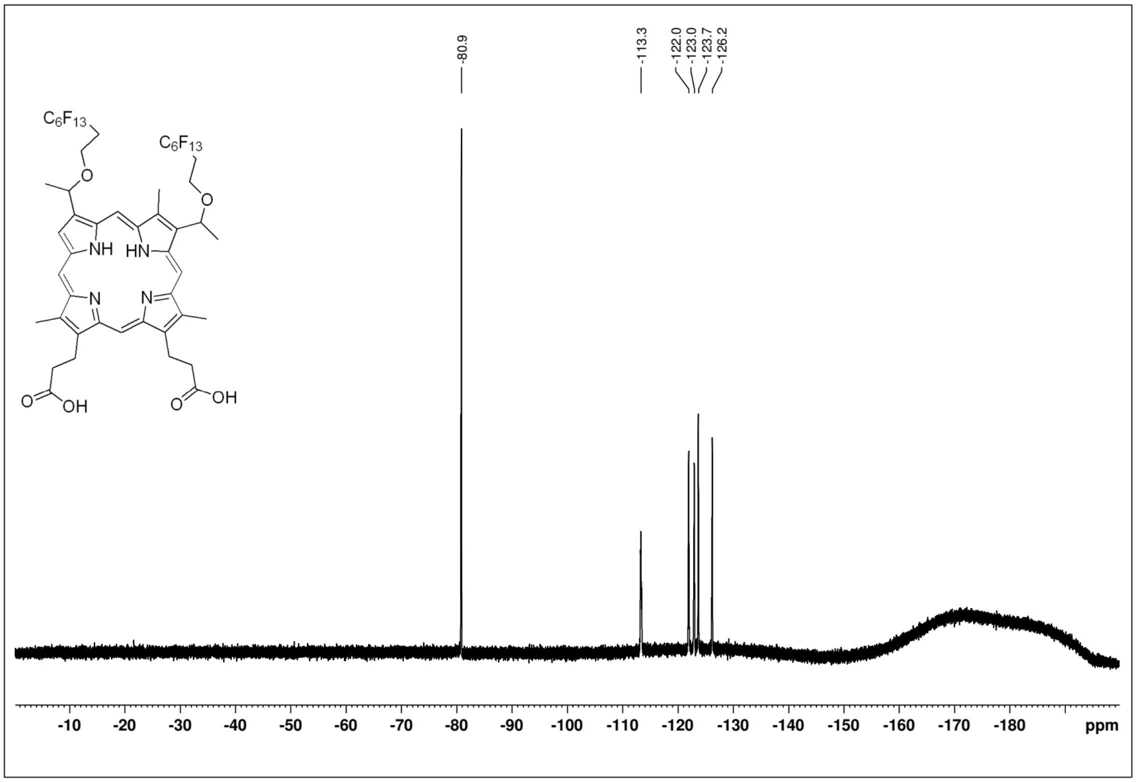

Figure S49: ^{13}C NMR spectrum of compound 4h in $CDCl_3$

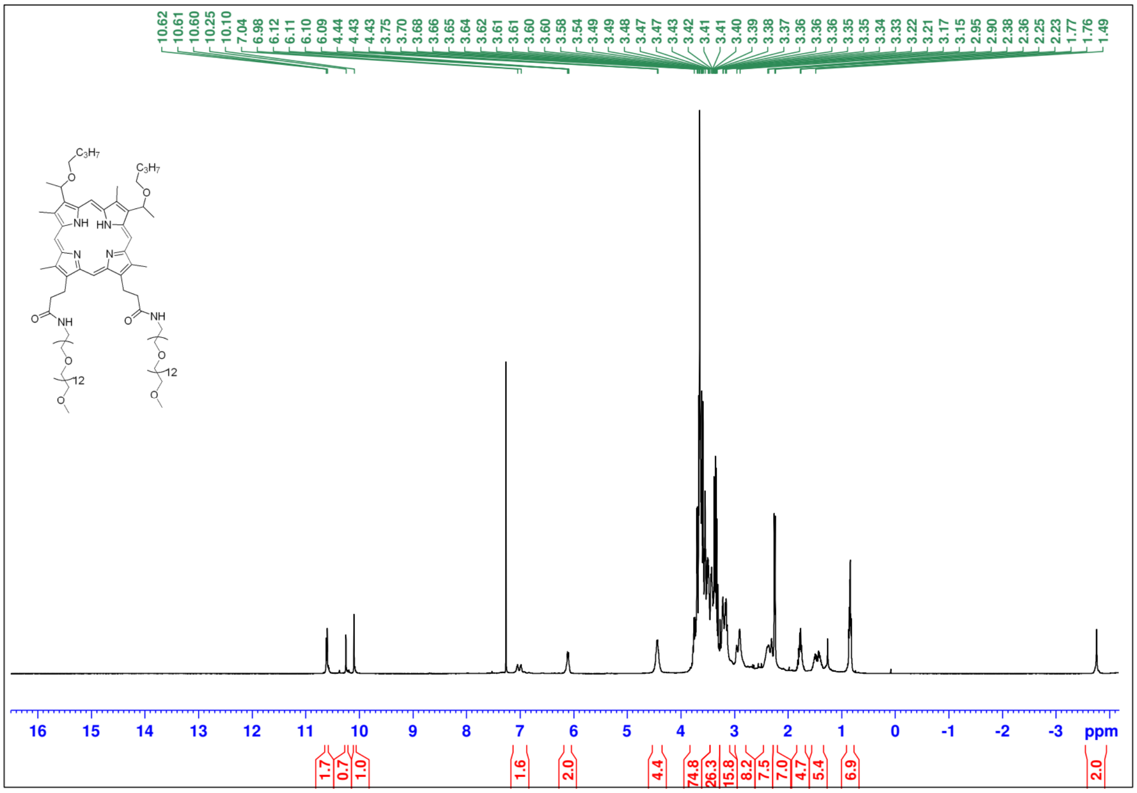

Figure S50: ^{1}H NMR spectrum of compound 5a in $CDCl_3$

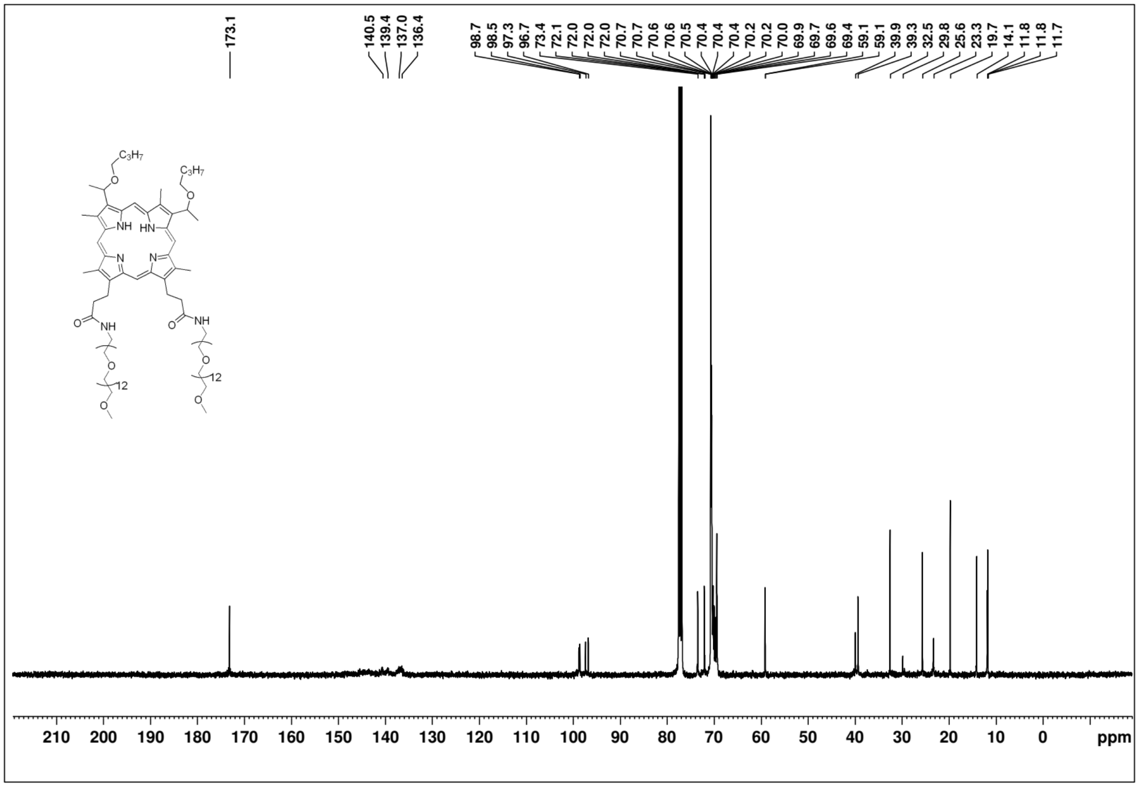

Figure S51: ^{13}C NMR spectrum of compound 5a in $CDCl_3$

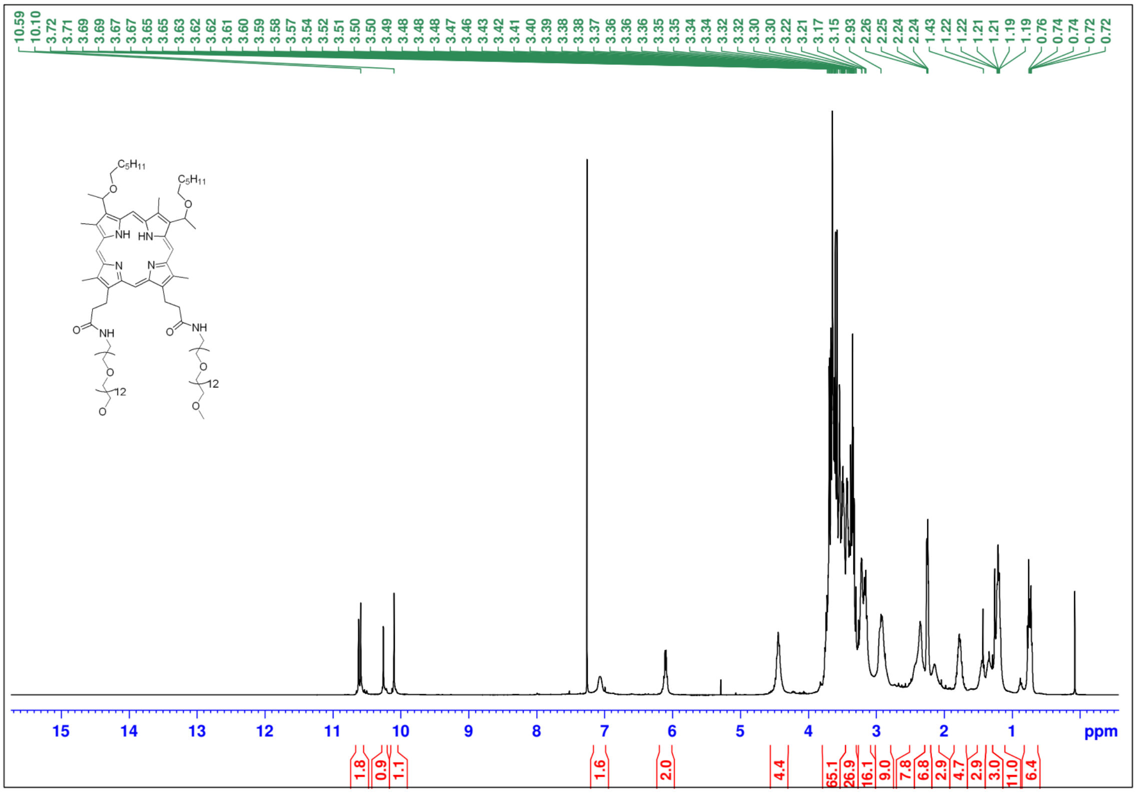

Figure S52: ^{1}H **NMR spectrum of compound 5b in** $CDCl_3$

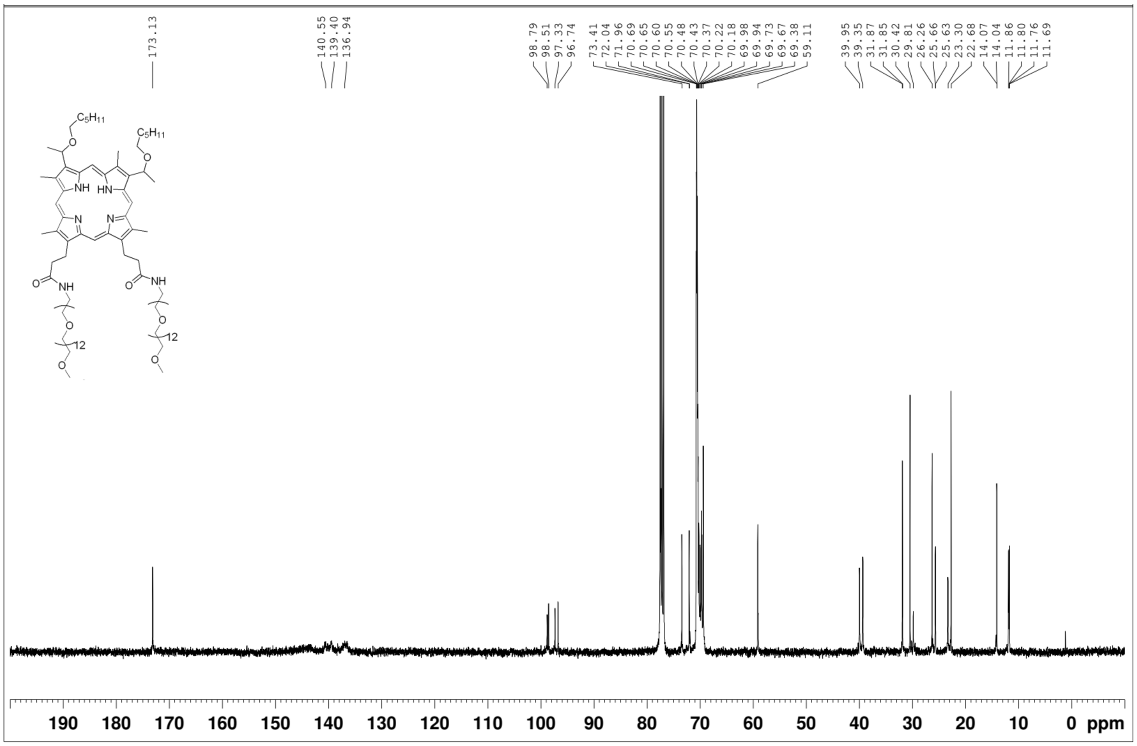

Figure S53: ^{13}C **NMR spectrum of compound 5b in** $CDCl_3$

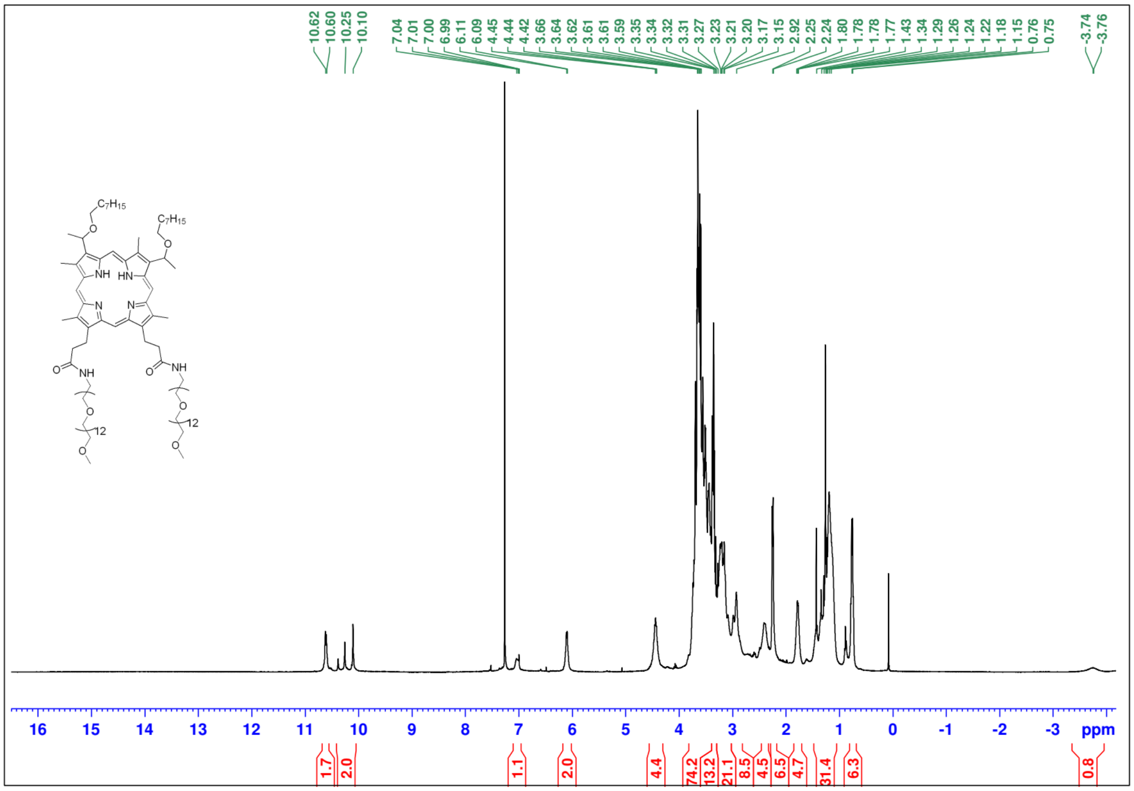

Figure S54: ^{1}H NMR spectrum of compound 5c in $CDCl_3$

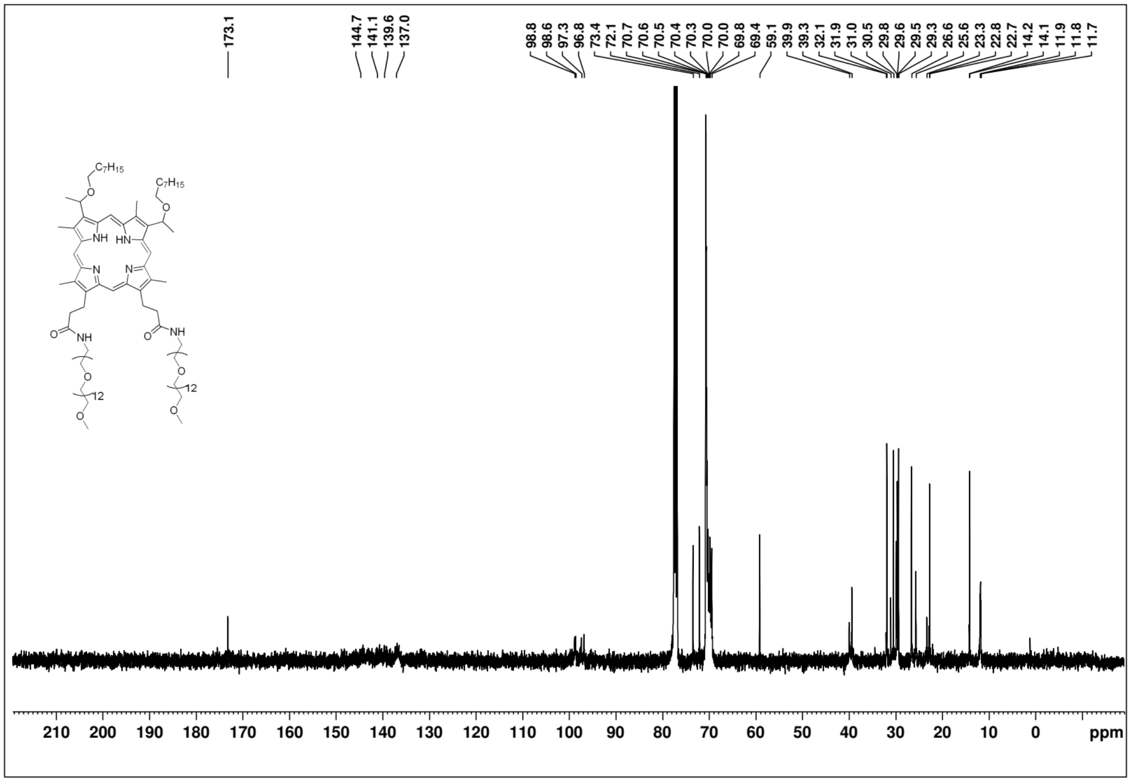

Figure S55: ^{13}C NMR spectrum of compound 5c in $CDCl_3$

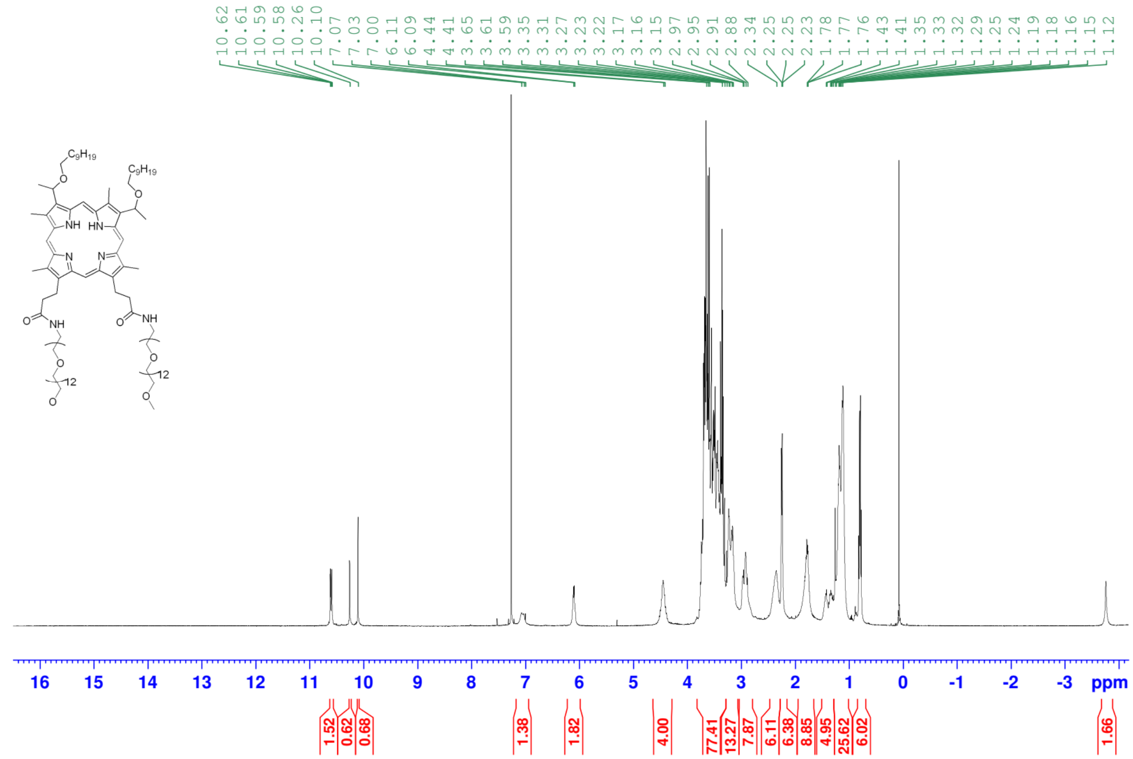

Figure S56: ^{1}H **NMR spectrum of compound 5d in** $CDCl_3$

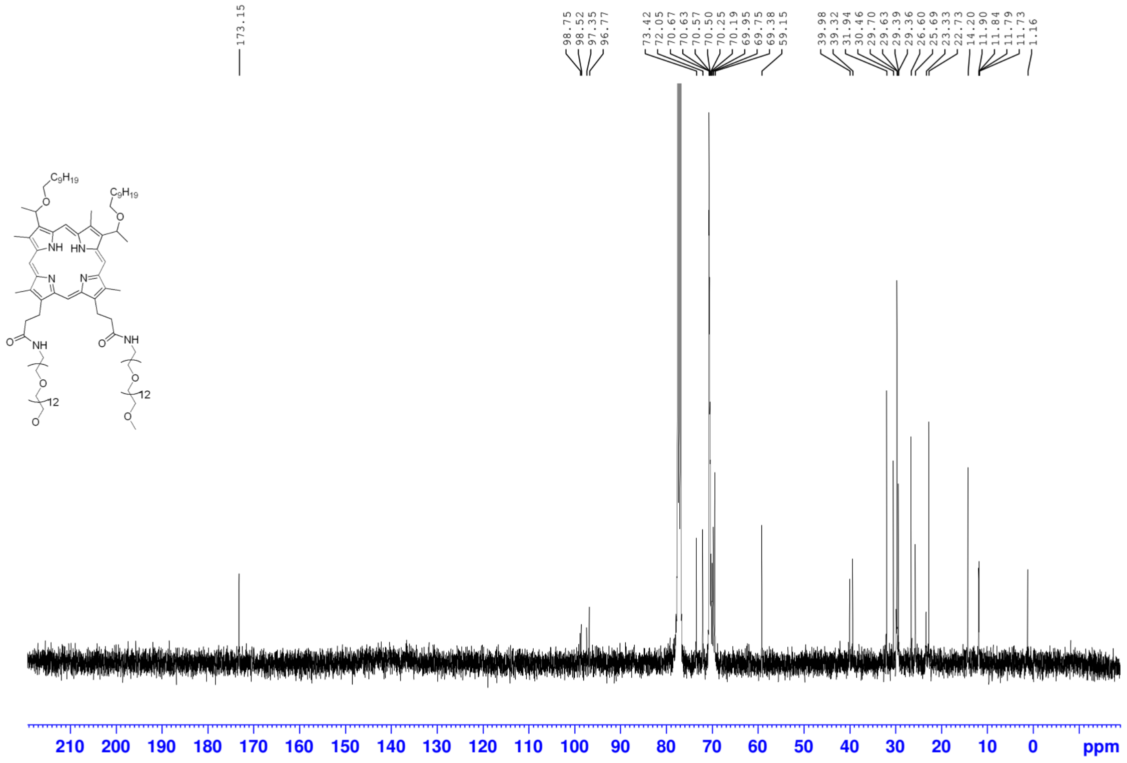

Figure S57: ^{13}C **NMR spectrum of compound 5d in** $CDCl_3$

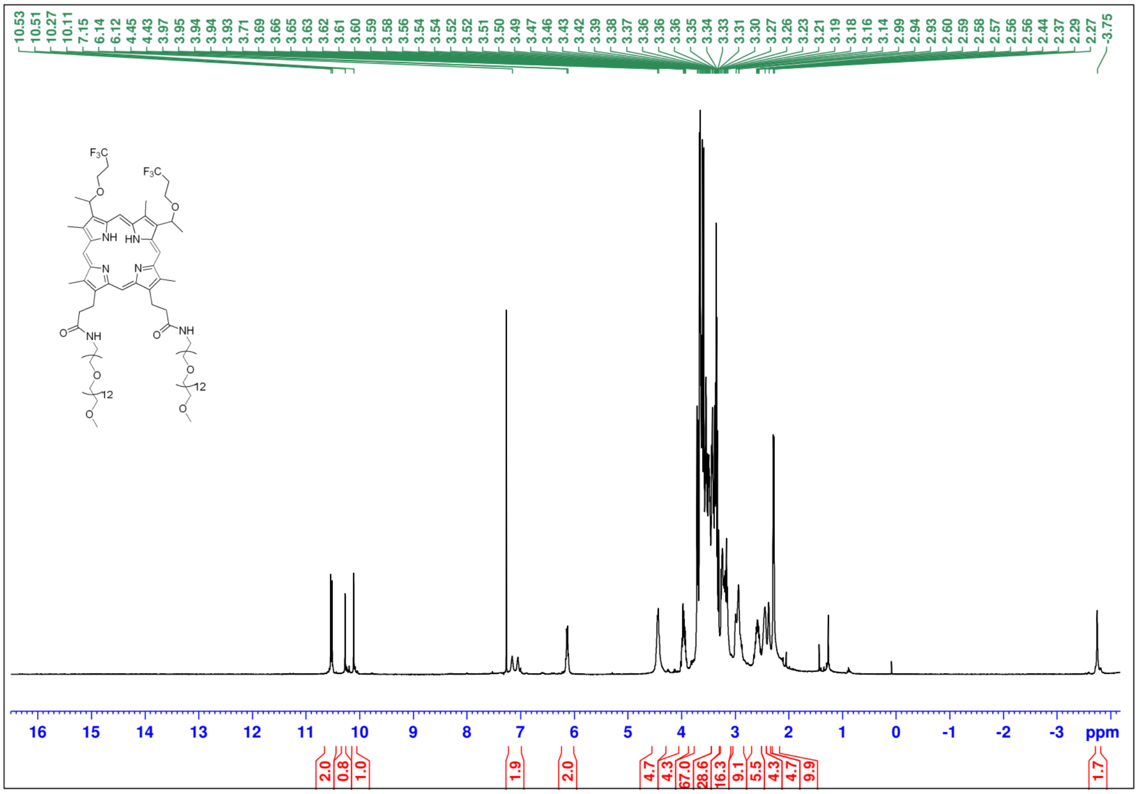

Figure S58: ^{1}H **NMR spectrum of compound 5e in** $CDCl_3$

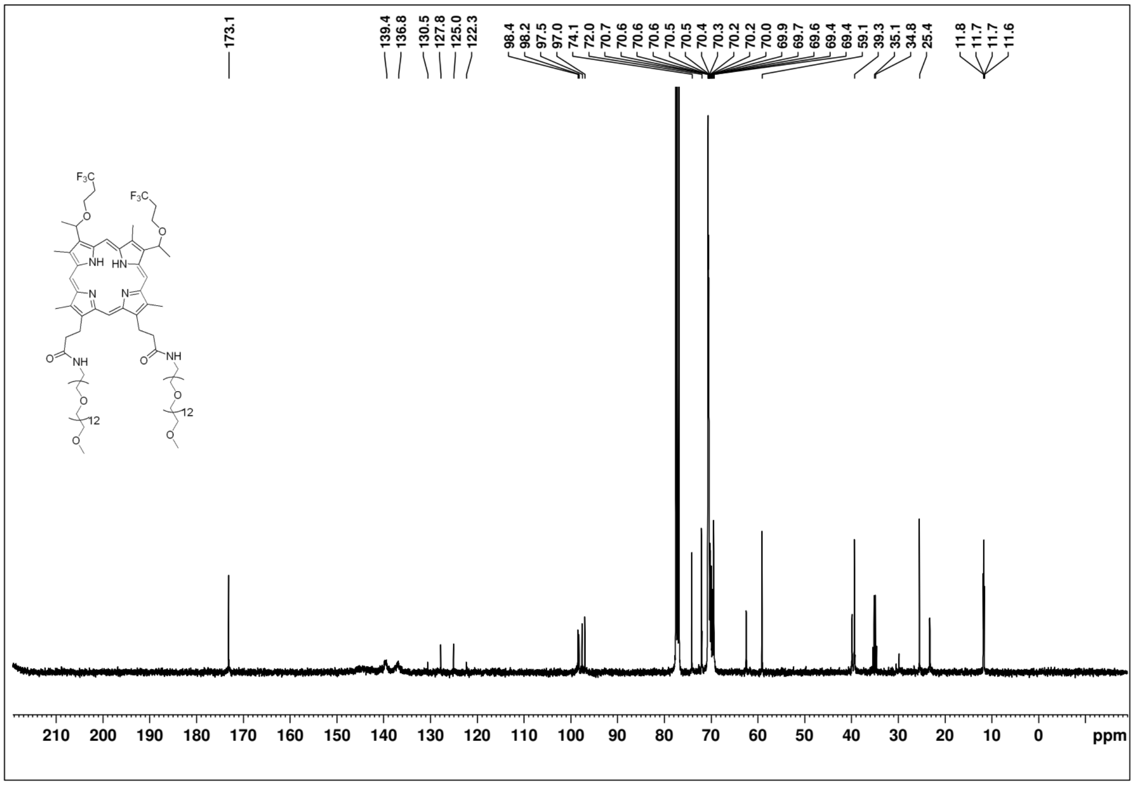

Figure S59: ^{13}C **NMR spectrum of compound 5e in** $CDCl_3$

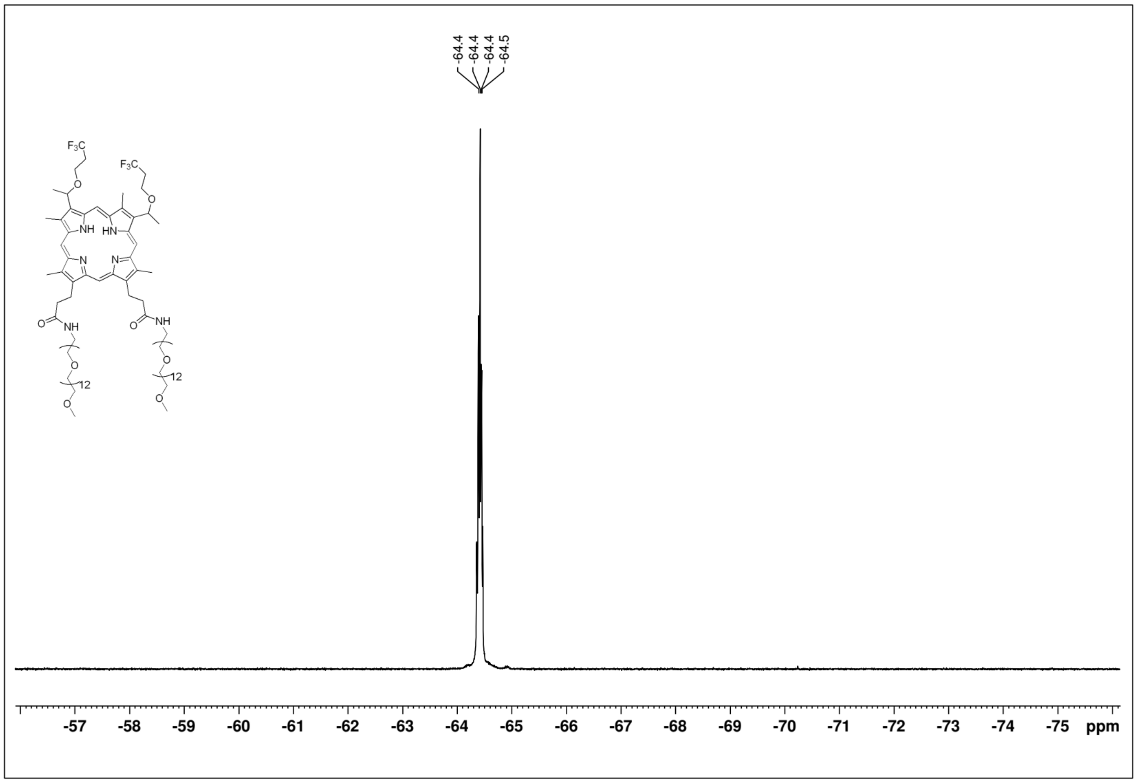

Figure S60: ^{19}F NMR spectrum of compound 5e in $CDCl_3$

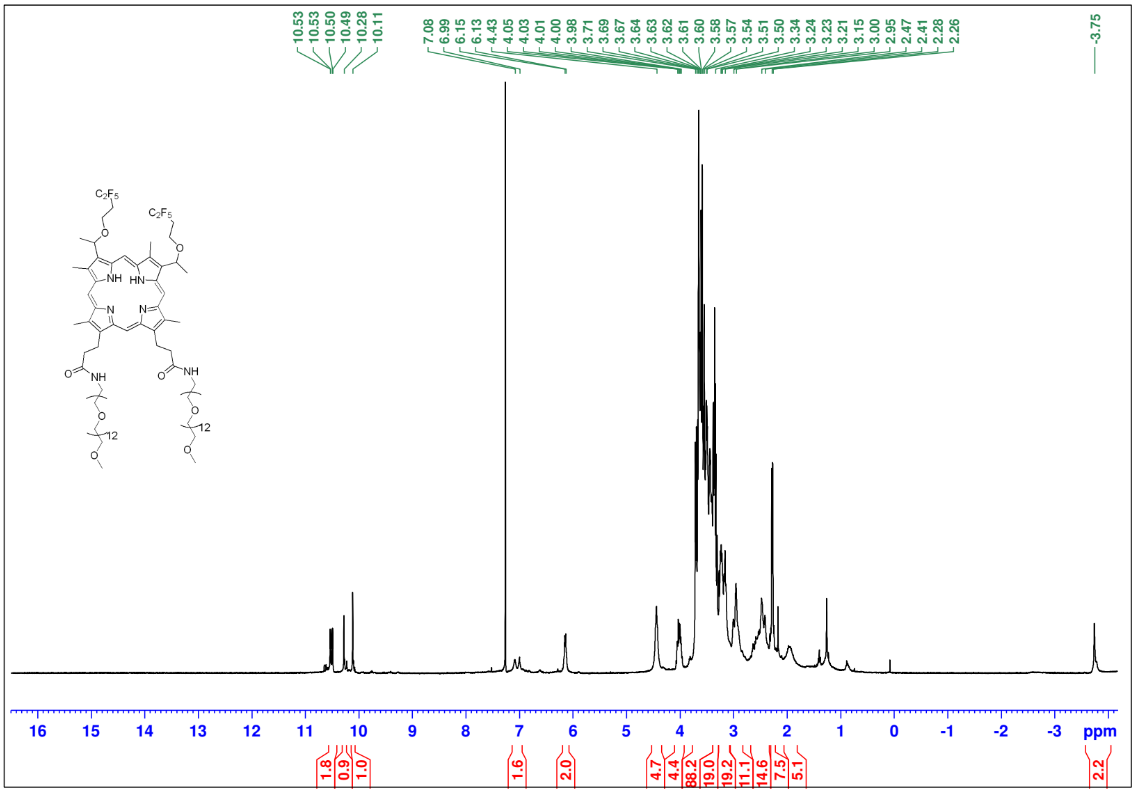

Figure S61: ^{1}H NMR spectrum of compound 5f in $CDCl_3$

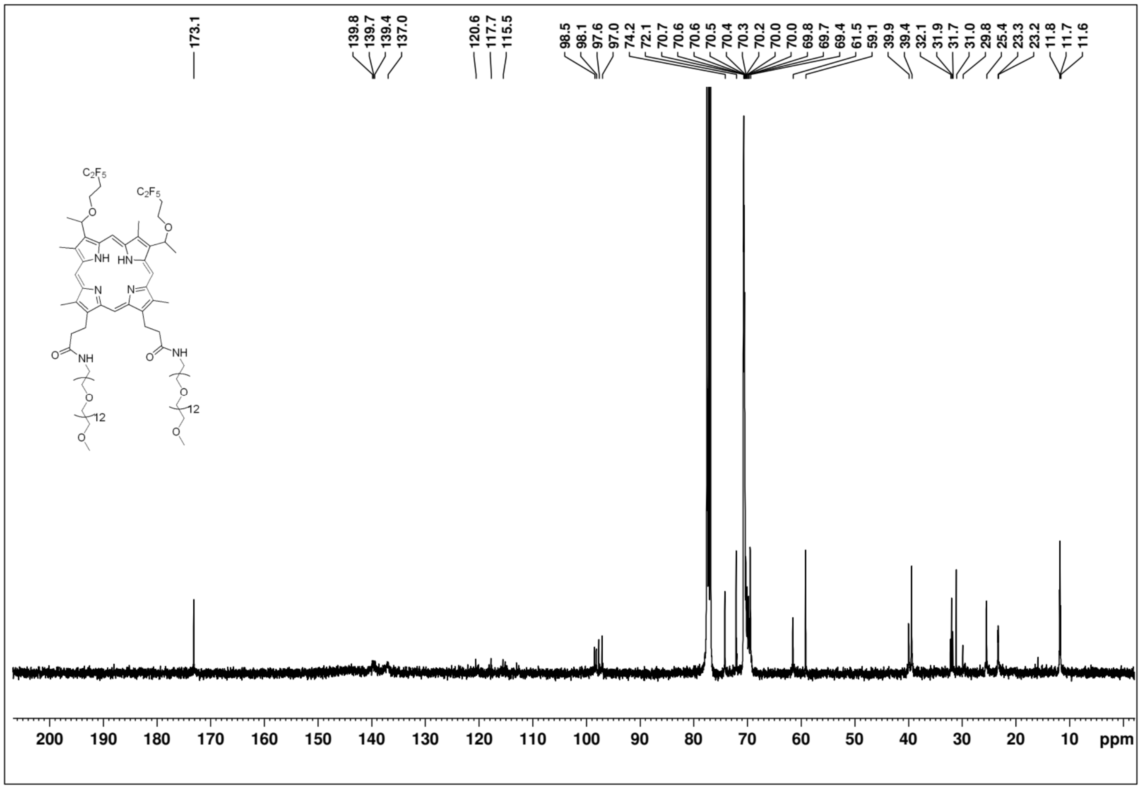

Figure S62: ^{13}C **NMR spectrum of compound 5f in** $CDCl_3$

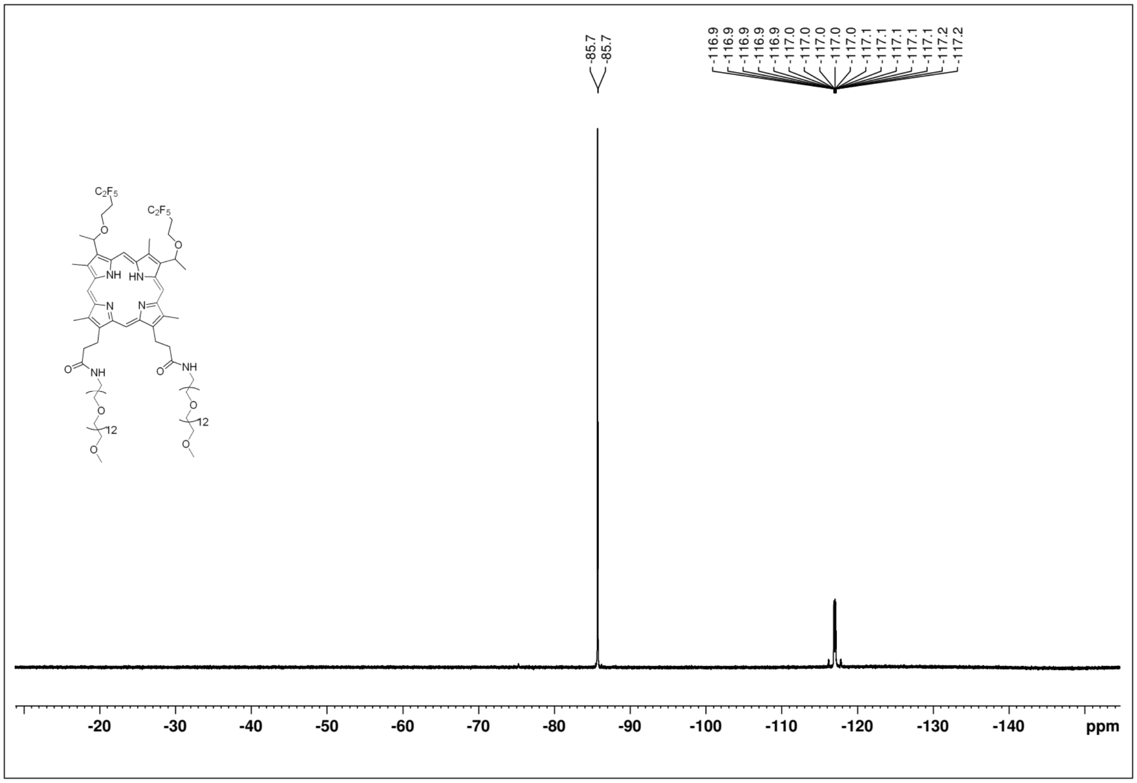

Figure S63: ^{19}F **NMR spectrum of compound 5f in** $CDCl_3$

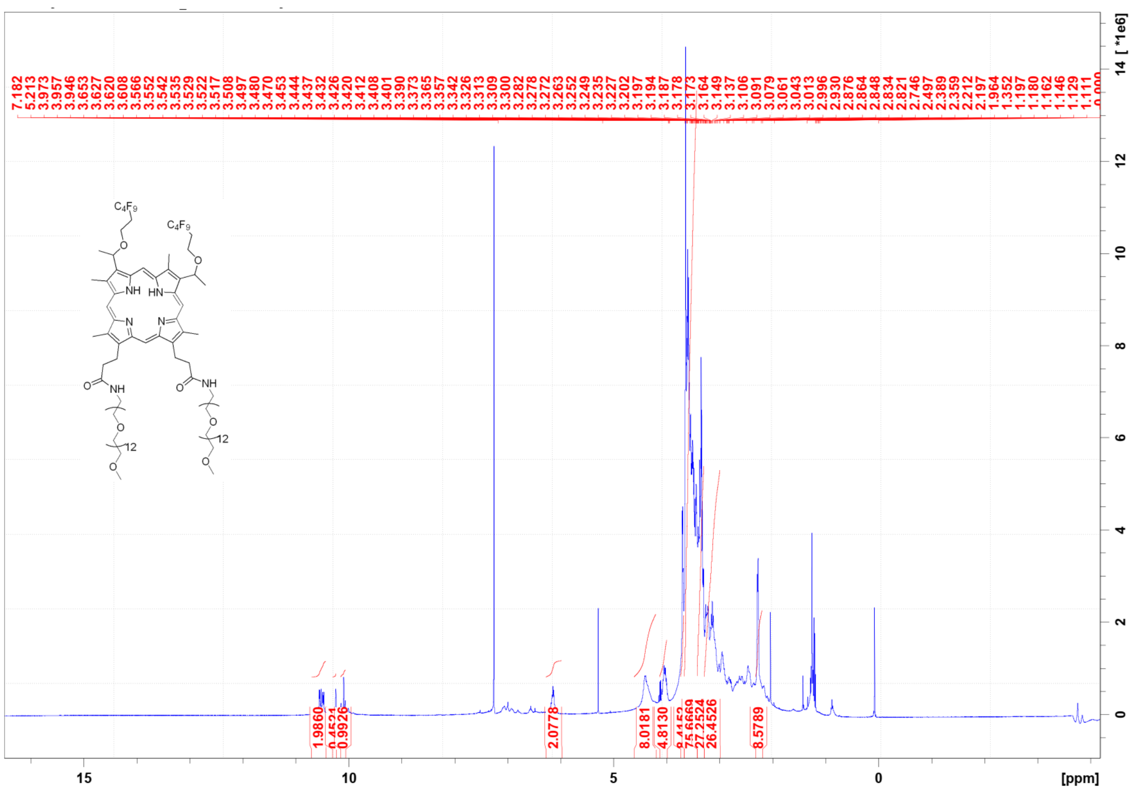

Figure S64: ^{1}**H NMR spectrum of compound 5g in $CDCl_3$**

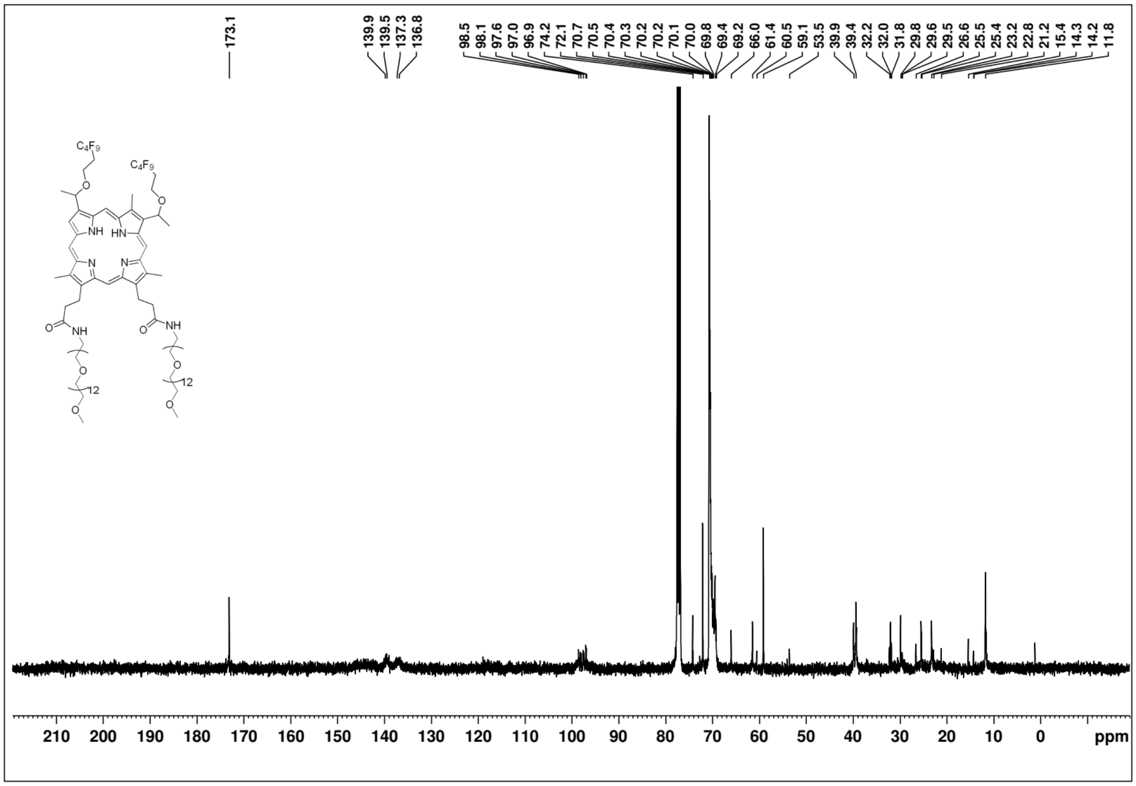

Figure S65: ^{13}**C NMR spectrum of compound 5g in $CDCl_3$**

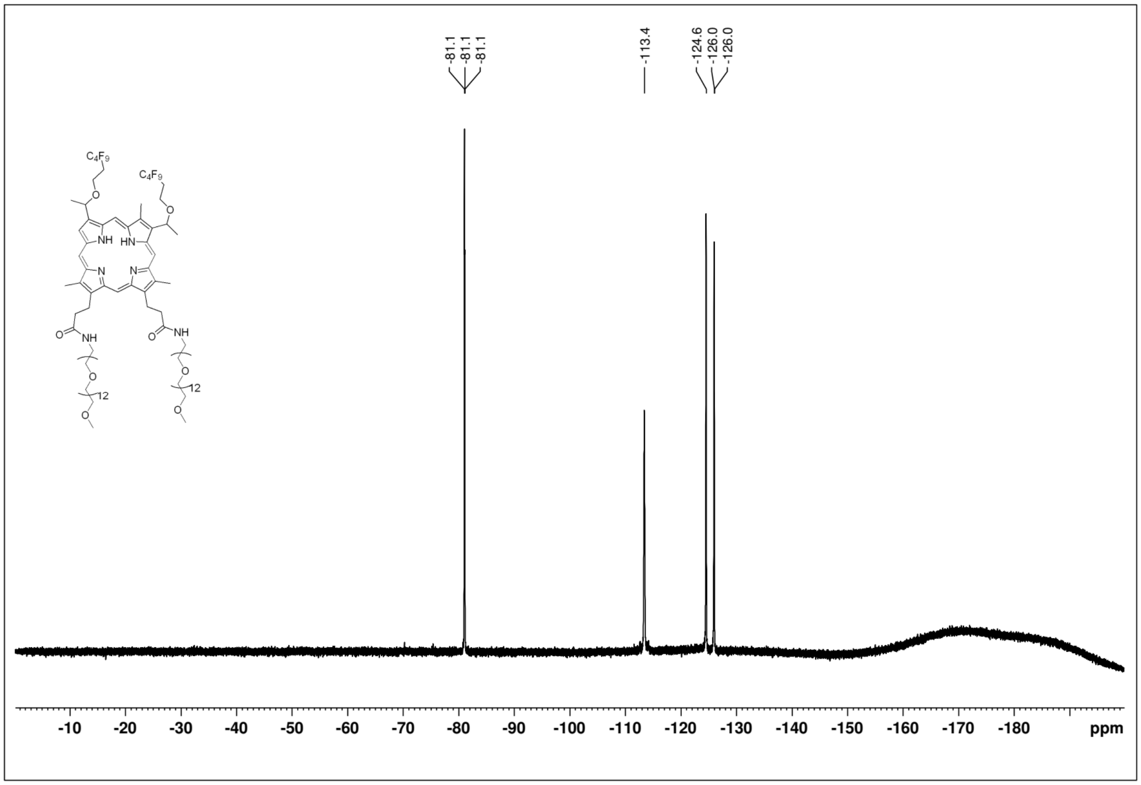

Figure S66: ^{19}F NMR spectrum of compound 5g in $CDCl_3$

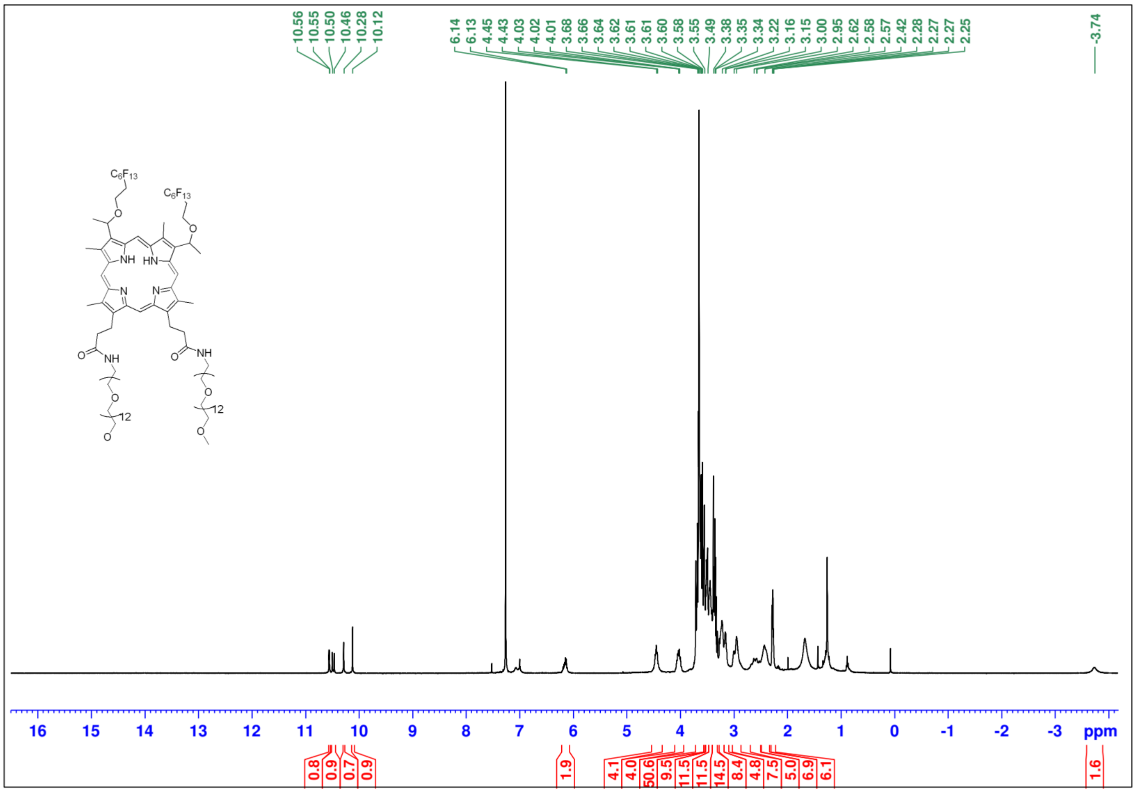

Figure S67: ^{1}H NMR spectrum of compound 5h in $CDCl_3$

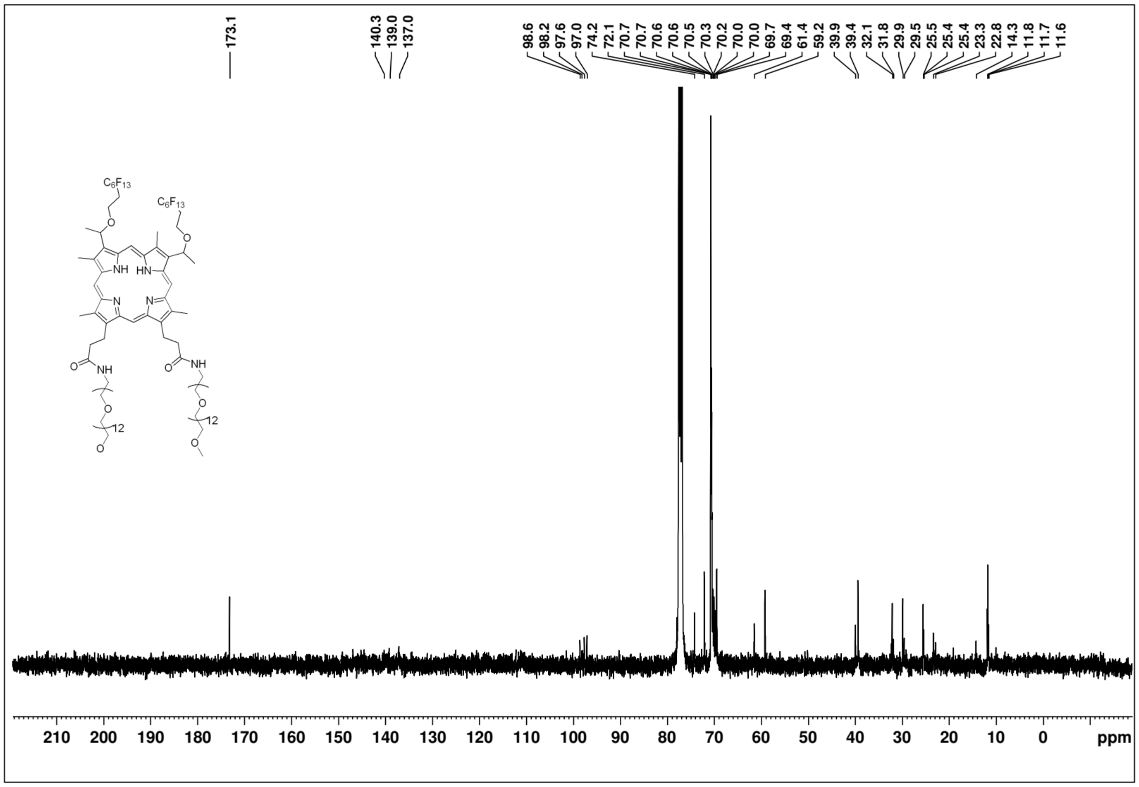

Figure S68: ^{13}C NMR spectrum of compound 5h in $CDCl_3$

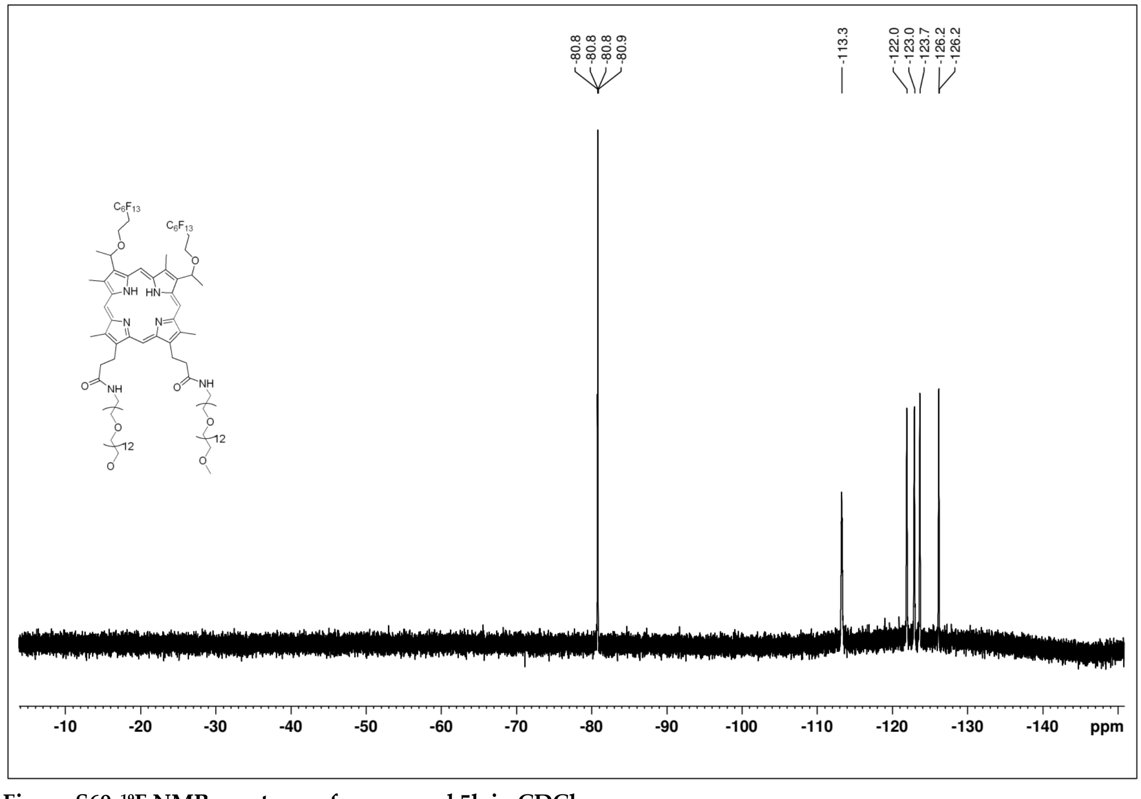

Figure S69: ^{19}F NMR spectrum of compound 5h in $CDCl_3$

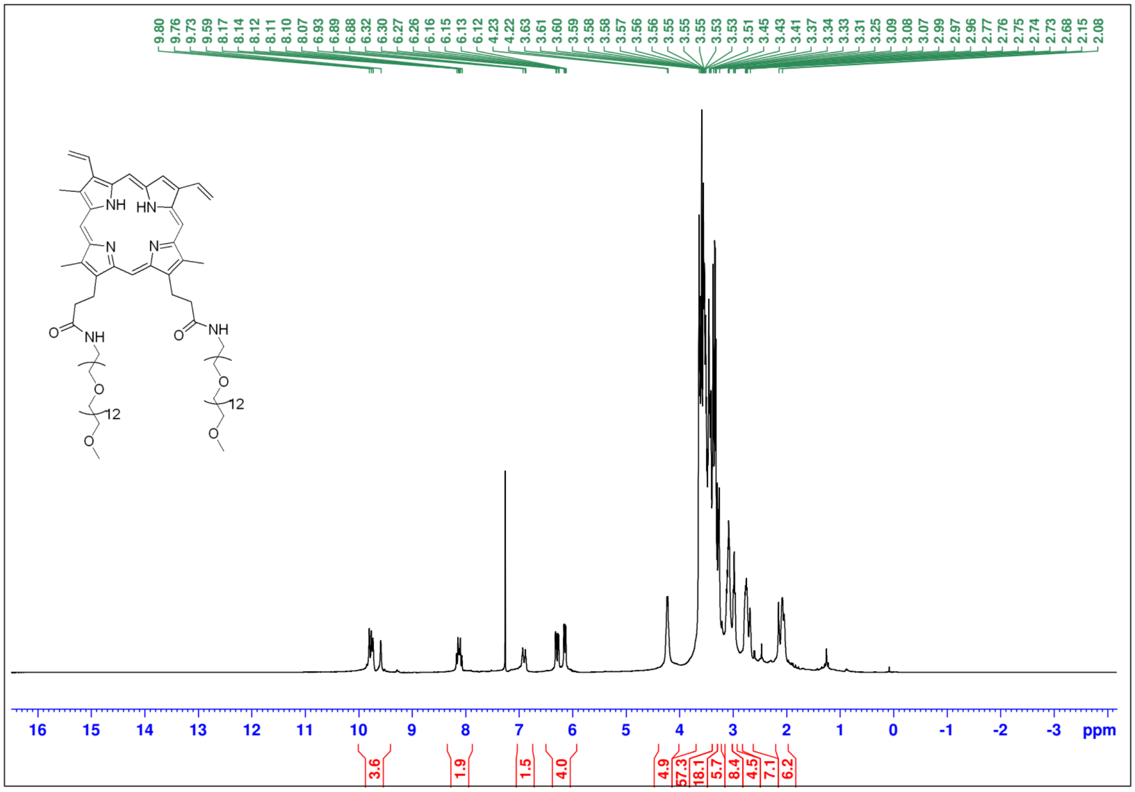

Figure S70: ^{1}H **NMR spectrum of compound 6 in** $CDCl_3$

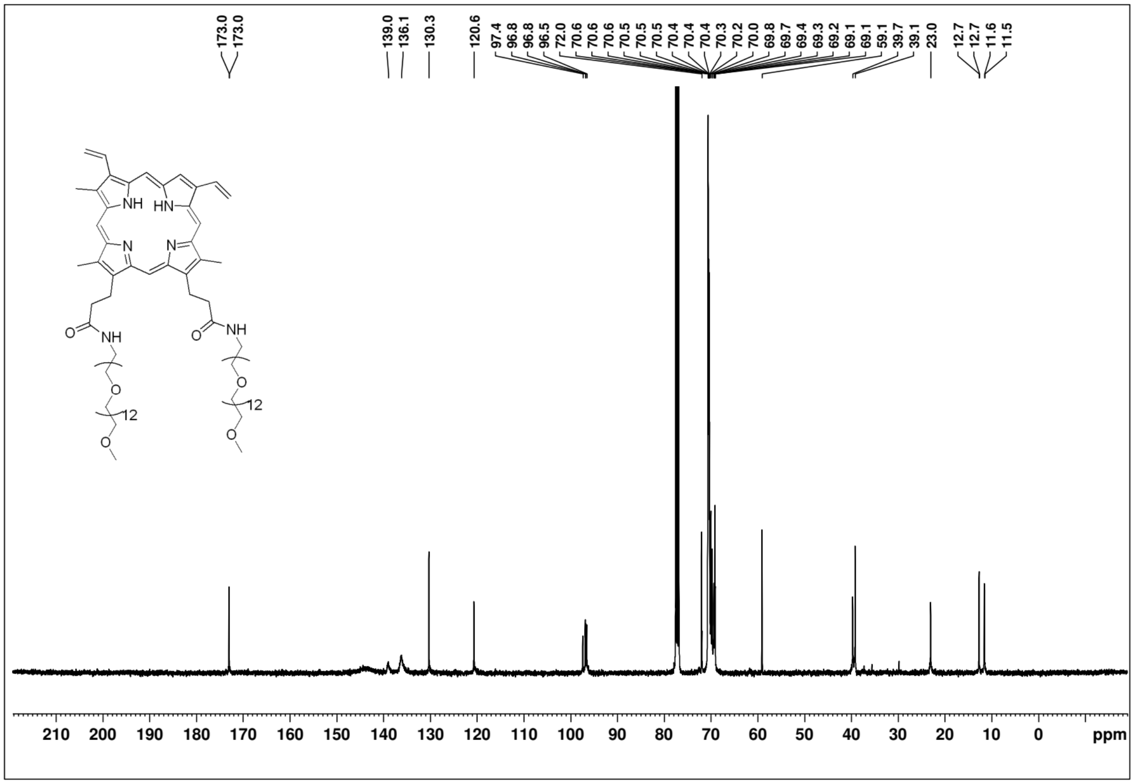

Figure S71: ^{13}C **NMR spectrum of compound 6 in** $CDCl_3$

1. Glimsdal, E.; Dragland, I.; Carlsson, M.; Eliasson, B.; Melø, T.B.; Lindgren, M. Triplet Excited States of Some Thiophene and Triazole Substituted Platinum(II) Acetylide Chromophores. *The Journal of Physical Chemistry A* **2009**, *113*, 3311-3320.
